# What ‘case definition’ for respiratory syncytial virus infection? Results of a systematic literature review to improve surveillance among the adults

**DOI:** 10.1093/pubmed/fdae066

**Published:** 2024-05-05

**Authors:** Emanuele Amodio, Miriam Belluzzo, Dario Genovese, Martina Palermo, Vincenzo Pisciotta, Francesco Vitale

**Affiliations:** Department of Health Promotion, Mother and Child Care, Internal Medicine and Medical Specialties ‘G. D’Alessandro’, University of Palermo, Via del Vespro 133, 90127 Palermo, Italy; Department of Health Promotion, Mother and Child Care, Internal Medicine and Medical Specialties ‘G. D’Alessandro’, University of Palermo, Via del Vespro 133, 90127 Palermo, Italy; Department of Health Promotion, Mother and Child Care, Internal Medicine and Medical Specialties ‘G. D’Alessandro’, University of Palermo, Via del Vespro 133, 90127 Palermo, Italy; Department of Health Promotion, Mother and Child Care, Internal Medicine and Medical Specialties ‘G. D’Alessandro’, University of Palermo, Via del Vespro 133, 90127 Palermo, Italy; Department of Health Promotion, Mother and Child Care, Internal Medicine and Medical Specialties ‘G. D’Alessandro’, University of Palermo, Via del Vespro 133, 90127 Palermo, Italy; Department of Health Promotion, Mother and Child Care, Internal Medicine and Medical Specialties ‘G. D’Alessandro’, University of Palermo, Via del Vespro 133, 90127 Palermo, Italy

**Keywords:** adult, case definition, elderly, hRSV, surveillance

## Abstract

**Background:**

Human respiratory syncytial virus (hRSV) is a leading cause of acute lower respiratory tract infection in frail individuals, including children, the elderly and immunocompromised people, with mild to severe symptoms. World Health Organization claims hRSV causes most elderly influenza-like illnesses (ILI) and severe acute respiratory infections (SARI). In this study, different case definitions for hRSV surveillance were examined for accuracy.

**Methods:**

The following search query (‘Respiratory Syncytial Virus’ OR ‘RSV’ OR ‘hRSV’ AND ‘case definition’) was used on PubMed/MEDLINE and Scopus with a 15-year-old baseline age restriction to conduct a systematic literature review.

**Results:**

Of 12 records, 58% employed the SARI definition, 50% the ILI definition and 42% the acute respiratory infection (ARI) definition, with some overlap. In young adults (18–64 years old), most studies show RSV prevalence between 6.25 and 72.54 cases per 1000 per year, and 19.23 to 98.5 in older adults. The outpatient ARI and hospitalized SARI criteria are particularly sensitive and specific.

**Conclusions:**

Disease burden measurement requires a clear case definition; however, current literature is questionable. Currently, hRSV surveillance uses numerous case definitions with debatable accuracy. The epidemiology, clinical characteristics, and disease burden of hRSV are difficult to characterize without a standard surveillance case definition.

## Background

Human respiratory syncytial virus (hRSV) poses a substantial burden on public health as it is a major cause of acute lower respiratory tract infection in children, the elderly and immunocompromised individuals.^1–4^ Human respiratory syncytial virus clinical manifestations more commonly involve the upper respiratory tract and include rhinitis, rhinorrhoea, cough and occasionally fever, developing—especially in infants, high-risk children, individuals with comorbidities and the elderly—in bronchiolitis, tracheobronchitis and pneumonia.^5–8^

However, if in infants bronchiolitis accounts for the most prevalent presentation of the hRSV-related disease, in older people hRSV infections are an increasing cause of parainfluenza illness, thus constituting one of the most common etiologies of influenza-like illness (ILI) and severe respiratory infections (SARI).^7–11^ A four-year cohort study revealed that 3%–7% of healthy elderly adults suffered from hRSV infection, with these percentages increasing up to 4%–10% among high-risk adults.^8^ However, a real burden of disease on a national basis is difficult to evaluate because hRSV diagnostic testing is not consistently done and detection of hRSV infections is further complicated by the absence of a uniform clinical case definition for hRSV infection and its non-specific symptoms.^12^^,^^13^

In this sense, across the decades, various case definitions of ILI have been employed worldwide for respiratory surveillance programs’ purposes, with the aim of minimizing the impact of the disease by planning appropriate control and intervention measures.^14^ To date, surveillance of hRSV circulation is based on the use of different case definitions whose accuracy may not be optimal and the WHO has recently underlined the importance of a no specific case-definition for hRSV.^15^ The fundamental challenge in looking for a more sensible case definition stems from the fact that symptoms are heterogeneous and not always clearly associated with a hRSV infection, thus determining a possible underreporting and underdiagnosis of the disease.

Due to the high morbidity and mortality associated with hRSV and the possible future availability of new preventive strategies, such as vaccinations and other preventative therapies, effective surveillance becomes crucial to curbing this significant public health threat. Thus, the present study aims to conduct a systematic literature review to determine the use of different case definitions, their accuracy, alongside the estimation of the burden of disease associated with each case definition. By elucidating the landscape of case definitions and their implications, this research endeavors to inform clinical management and public health surveillance strategies, ultimately resulting in the determination of the most suitable case definition(s) for the detection of hRSV cases.

## Methods

### Research strategy and selection criteria

Preferred reporting items for systematic reviews and meta-analyses (PRISMA) guidelines were used to report the process and results.

A systematic literature review was performed to define the use of different case definitions and their performance. The latter was conducted using a search string developed by the research group of the Laboratory of Epidemiology applied to Biomedical and Environmental Sciences of the Department of Health Promotion, Mother and Child Care, Internal Medicine and Medical Specialties of the University of Palermo. The research groups includes medical doctors who have carried several studies on the epidemiology of respiratory-driven infections, including hRSV.^16^ The resulting search string was the following: (‘Respiratory Syncytial Virus’ OR ‘RSV’ OR ‘hRSV’ AND ‘case definition’) on PubMed/MEDLINE and Scopus during the period of December 2022. All studies published in English, conducted on humans, and on non-pediatric individuals (>14 years) were included in this systematic review. There were no restrictions on study design, sample characteristics, geographic area and publication date. Each article that did not match the aforementioned inclusion criteria was consequently excluded. Initially there were 125 articles selected, all in English; of these, 45 were duplicates and 68 were excluded after subsequent evaluation.

### Data collection

Two authors independently reviewed the titles and abstracts of the studies by assessing methodological quality and extracting full-text data; the latter were also checked by a third author. Issues were resolved by consensus of the entire team. Data were extracted for: citation, study period, study type, publication year, geographic location, study population, sample size and age group case definitions used. Sensitivity and specificity of case definitions were assessed if present, and hRSV prevalence in percent in articles in which it was absent was calculated.

### Data analysis

A descriptive analysis was performed to identify the prevalence of the most appropriate case definition. Sensitivity, specificity, and prevalence were reported as relative frequencies (%). R for Statistical Computing (version 4.2.2, Vienna, Austria) within the RStudio interface (RStudio, PBC, Boston, Massachusetts) and Microsoft Excel (version 2305, Redmond, Washington) software were used for statistical analysis of the data and for drawing figures.^17^^,^^18^

## Results

### Literature search

**Fig. 1:**
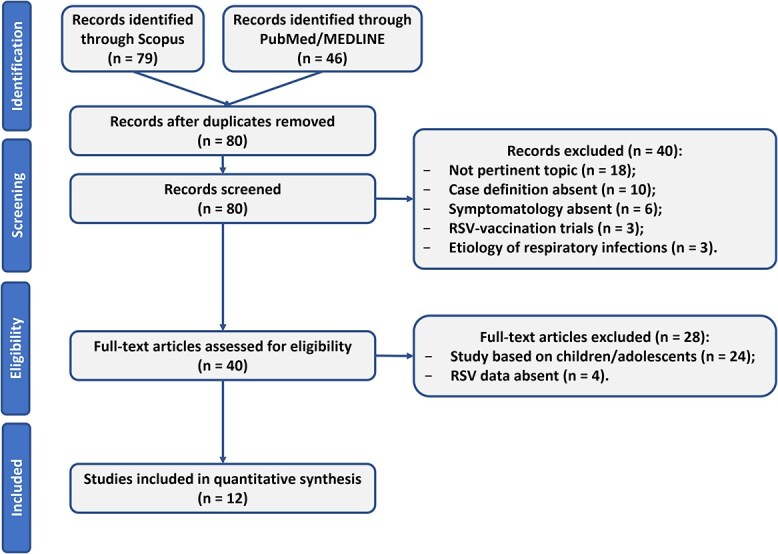
Flowchart representing PRISMA flow diagram of studies’ screening and selection.

Figure 1 shows the record selection process. Overall, 125 records were identified through the PubMed/MEDLINE (46) and Scopus (79) search platforms; of these, 45 were duplicates and have, therefore, been removed.

Out of the 80 articles, 40 were excluded during the screening phase for the following reasons: 18 records were related to other topics without mention of hRSV (45%); 10 articles did not report the case definition (25%); 6 articles did not mention symptomatology (15%); the remaining articles referred to hRSV vaccine trials (3; 7.5%) and the etiology of respiratory infections (3; 7.5%).

The selection brought to 40 articles assessable for the eligibility phase. Of these, 28 were excluded because they analyzed exclusively the pediatric population (24; 86%) or because they did not analyse hRSV (4; 14%).

At the end of the eligibility phase, 12 studies were included in the systematic review.^13^^,^^15^^,^^19–28^

### Characteristics of the included studies

Table 1 summarizes the characteristics of the twelve articles included by the group of researchers. Overall, 9/12 reports (75%) adopted a prospective study design,^13^^,^^19^^,^^20^^,^^22^^,^^23^^,^^25–28^ while the remaining articles were constituted of two clinical trials (17%) and one retrospective study (8%).^15^^,^^21^^,^^24^

**Table 1:** Characteristics of the 12 articles included by the group of researchers

Reference article [reference no]	Title	Publication year	Country	Study design	Study period	No of participants	Age range(s)	Adopted case definition
Bellei et al.^18^	Acute respiratory infection and influenza-like illness viral etiologies in Brazilian adults	2008	Brazil	Prospective	2001–2003	420	18+	ILI and ARI
Feikin et al.^19^	Etiology and incidence of viral and bacterial acute respiratory illness among older children and adults in rural western Kenya, 2007–2010	2012	Kenya	Prospective	2007–2010	500	18–49; 50+	ARI
Radin et al.^20^	Epidemiology of pathogen-specific respiratory infections among three US populations	2014	United States of America	Clinical trial	2011–2013	314	25–49; 50+	ILI and SARI
Campe et al.^21^	Clinical symptoms cannot predict influenza infection during the 2013 influenza season in Bavaria, Germany	2015	Germany	Prospective	2013	323	15–59; 60+	ILI and ARI
Wansaula et al.^22^	Surveillance for severe acute respiratory infections in Southern Arizona, 2010–2014	2016	United States of America	Prospective	2010–2014	303	25–64; 65+	SARI
Cui et al.^23^	Clinical and epidemiologic characteristics of hospitalized patients with laboratory-confirmed respiratory syncytial virus infection in Eastern China between 2009 and 2013: a retrospective study	2016	China	Retrospective	2009–2013	12	60+	SARI
Hirve et al.^24^	Clinical characteristics, predictors, and performance of case definition-Interim results from the WHO global respiratory syncytial virus surveillance pilot	2019	Argentina, Brazil, Chile, Côte d'Ivoire, Egypt, India, Mongolia, Mozambique, Russian Federation, South Africa, Thailand, United Kingdom	Prospective	2017–2018	6340	18–64; 65+	ILI; SARI
Sáez-López et al.^13^	Performance of surveillance case definitions for respiratory syncytial virus infections through the sentinel influenza surveillance system, Portugal, 2010 to 2018	2019	Portugal	Prospective	2010–2018	1134	15–64; 65+	ILI and ARI
Hatem et al.^25^	Clinical characteristics and outcomes of patients with severe acute respiratory infections (SARI): results from the Egyptian surveillance study 2010–2014	2019	Egypt	Prospective	2010–2014	94	18+	SARI
Subissi et al.^26^	Capturing respiratory syncytial virus season in Belgium using the influenza severe acute respiratory infection surveillance network, season 2018/19	2020	Belgium	Prospective	2018–2019	128	65+	SARI
Davis et al.^27^	Sensitivity and specificity of surveillance case definitions in detection of influenza and respiratory syncytial virus among hospitalized patients, New Zealand, 2012–2016	2021	New Zealand	Prospective	2012–2016	2900	18–64; 65+	SARI
Korsten et al.^15^	World Health Organization influenza-like illness underestimates the burden of respiratory syncytial virus infection in community-dwelling older adults	2022	Netherlands, Belgium, United Kingdom	Clinical trial	2017–2019	750	60+	ILI and ARI

With respect to the country, 4/12 articles (33%) were carried out in European countries, 3/12 reports (25%) were conducted in America, 2/12 records (17%) took place in Africa, one report was carried out in China,^24^ one article was conducted in New Zealand,^28^ and a further study was conducted in several countries worldwide.^25^ All the articles were published after 2007 and 75% of the studies after 2015.

Looking more in-depth, the selected studies adopted one or more of the following case definitions:

(a) ILI: Any person who presents sudden and rapid onset of at least one of the following general symptoms: fever or feverishness, malaise/exhaustion, headache, muscle pain and, at least one of the following respiratory symptoms: cough, sore throat or wheezing.
(b) ARI: Acute respiratory infection arising within the last 10 days with at least one symptom among cough, sore throat, difficulty breathing or rhinorrhoea.
(c) SARI: The World Health Organization (WHO) defines SARI as a severe acute respiratory infection with fever ≥38°C, onset (or re-exacerbation in the case of a patient with chronic respiratory disease) of cough or other respiratory symptoms, with onset of symptoms within 10 days prior to triage (acute) and requiring hospitalization (severe).

In particular, 7/12 articles (58%) decided to include SARI cases; 6/12 reports (50%) adopted ILI as case definition; 5/12 reports (42%) adopted ARI as case definition; 4/12 reports (33%) adopted indistinctly ILI and ARI as case definition; and 2 articles (17%) decided to use both ILI and SARI definitions.

Considering age, 3/12 reports (25%) included the elderly population; 2/12 articles (17%) considered the adult population without discerning the young adults from the old ones; and the remaining 7/12 reports (58%) considered both the adult population and the elderly, distinguishing the two cohorts.

The number of participants was less than 100 patients in 2/12 articles (17%), between 100 and 500 participants in 6/12 (50%) manuscripts, and more than 500 patients in the remaining 4 articles (33%).

### Prevalence of hRSV

**Fig. 2:**
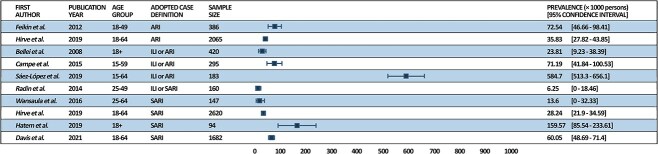
Prevalence of hRSV cases in the young adult population.

As reported in Fig. 2, among young adult population (18 to 64 years old) 7/10 studies observed a prevalence ranging from 23 to 72.54 cases per 1000, 2/10 less than 20 cases per 1000 whereas Hatem et al. found a prevalence of 159.6 cases per 1000 SARI cases.

**Fig. 3:**
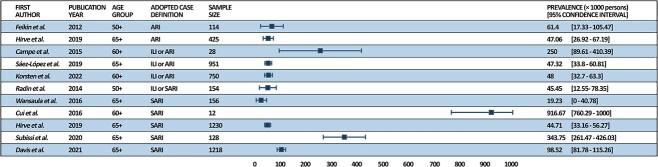
Prevalence of hRSV cases among the over 50 years old population.

As reported in Fig. 3, among older adults 7/11 studies reported a prevalence ranging between 47.06 and 98.5 per 1000 subjects per year, 1/11 less than 20 cases per 1000 while Cui et al. found the highest prevalence of 916.7 positive patients per 1000 persons (although only 12 patients were included in this study). Subissi et al. identified a prevalence of 343.75 hRSV-positive patients per 1000 SARI patients; similarly, Campe et al. found a prevalence of 250 cases each 1000 ILI or ARI patients.

At first glance, comparing our findings to those of the systematic review with meta-analyses conducted by Savic et al.,^29^ it appears that the papers we included have a higher prevalence. The systematic review by Savic et al.^29^ aimed to estimate the RSV disease burden in 2019 in high-income countries among adults aged 60 or more, considering attack rates, hospitalization rates and in-hospital case fatality rates. The meta-analysis revealed a pooled estimate of 16.2 RSV-ARI attack rates per 1000 persons. It should be highlighted that only studies that adopted the ARI case definition were included, and only those from high-income countries. Savic et al.^29^ further selected only one age group, those over 60, whereas we aimed to conduct a systematic review that would provide an overview of non-pediatric individuals. These discrepancies may have influenced the differences in results.

### Sensitivity, specificity and accuracy of case definitions

**Fig. 4:**
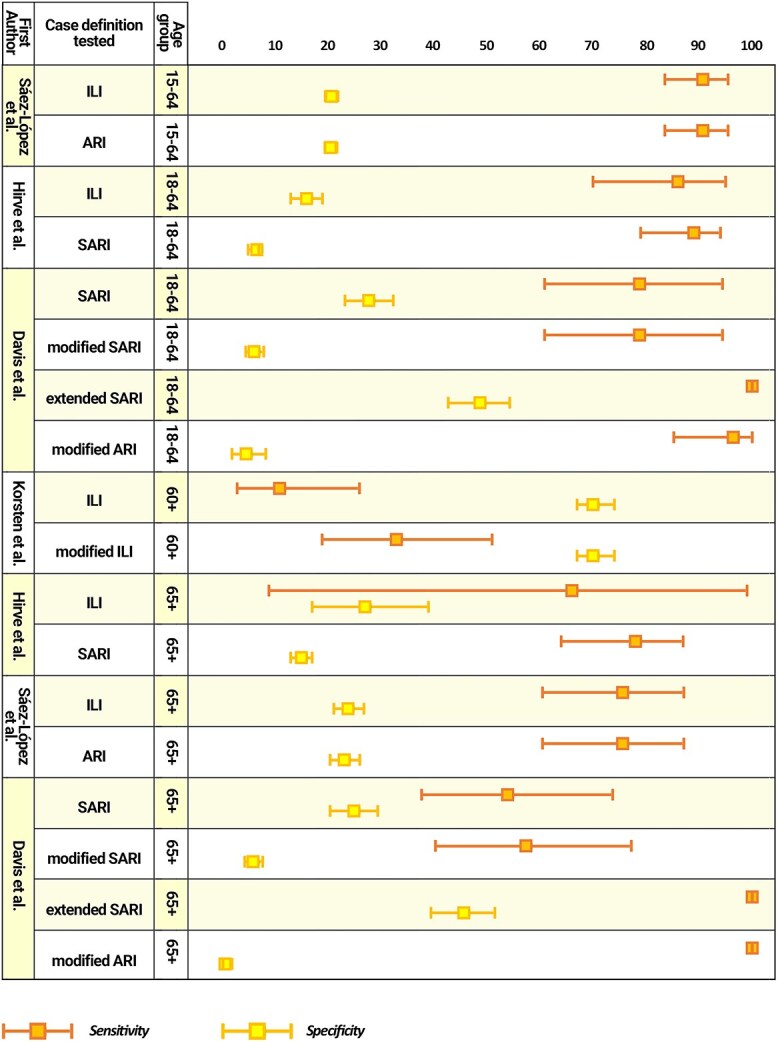
Sensitivity, specificity and accuracy of case definitions.

As reported in Fig. 4, only 4/12 articles (33%) assessed the sensitivity, specificity and accuracy of the case definitions adopted in their surveillance programs.

Hirve et al.^25^ based their surveillance on the extended SARI and ILI case definitions for the in-patients and the out-patients, respectively. Taking as reference the adopted definitions, the authors found out that, among the adult inpatients aged between 18 and 64 years, the SARI definition had a sensitivity of 89% (79–94), a specificity of 6.6% (5–7) and an area under the curve (AUC) of 0.47. Similarly, among the non–hospitalized counterpart, the ILI definition had a sensitivity of 86% (70–95), a specificity of 16% (13–19) and a AUC of 0.51. Considering the elderly, among the hospitalized patients the SARI case definition showed a sensitivity of 78% (64–87), a specificity of 15% (13–17) and an AUC of 0.46; at the same time, between the outpatients the ILI case definition had a sensitivity of 66% (9–99), a specificity of 27% (17–39) and a AUC of 0.46.

Sáez–López et al.^13^ analyzed the results of the sentinel Portuguese surveillance system. It was shown that both the EU ILI definition and the modified EU ARI definition had a sensitivity of 90.7% (83.5–95.4) among the adults between 15 and 64, and of 75.6% (60.5–87.1) in the elderly population. The AUC was around 0.5 for each subgroup analyzed.

Davis et al.^28^ evaluated the sensitivity and specificity of several case definitions in New Zealand. Among the patients aged 18–64 years, SARI and modified SARI definitions had lower sensitivity than extended SARI and modified ARI definitions [78.8% (60.9–94.4) versus 100% (100–100)] and, at the same time, higher specificity [27.7% (23.2–32.3) and 24.9% (20.4–29.4) versus 6.1% (4.5–7.9) and 5.9% (4.3–7.7)]. Similarly, when considering the elderly, SARI and modified SARI definitions had lower sensitivity than extended SARI and modified ARI definitions (53.9% (37.7–73.7) and 57.4% (40.3–77.2) versus 96.4% (85.2–100) and 100% (100–100)) and higher specificity (48.7% (42.7–54.3) and 45.6% (39.4–51.5) versus 4.6% (1.9–8.3) and 0.6% (0.2–1.9)).

Finally, Korsten et al.^15^ tested the accuracy of WHO-ILI and a modified version of the previously cited definition (modified ILI, which considers as case each subject feeling feverish, without evidence of fever) in the identification of RSV cases among the older adults, finding that the sensitivity of both the case definitions was low: 11% (3–26) for WHO-ILI, and 33% (19–51) for modified ILI. Specificity of WHO-ILI was 93% (91–95), while that of modified ILI was 70% (67–74); AUC was in both cases 0.52.

## Discussion

### Main findings of this study

This systematic review aimed to identify the role that case definition could have had in the understanding of the epidemiology of hRSV in the adult population, as well as to provide information that could enhance future research for hRSV epidemiological surveillance.

The key message of this review is that the investigated studies used several case definitions and, in some case, overlapped different case definitions in the effort to be more sensitive. For instance, 58% of the studies used the SARI-WHO definition and 50% adopted ILI-WHO definition, whereas 42% used the definition of acute respiratory infections (ARI) with some cases of overlap between the case definitions. This considerable heterogeneity prevented us from calculating the overall hRSV prevalence and it also resulted in a large variability of the measures of sensitivity, specificity and accuracy calculated for the different case definitions*.* In the absence of an unambiguous case definition, there is a significant risk that different surveillance systems could measure the burden of disease in radically different ways.

Overall, the review seems to suggest that, for hRSV case detection, the modified ARI and extended SARI case definitions (in which fever is not included) are more sensitive than SARI and modified SARI definitions and, similarly, the ILI definition has low sensitivity but higher specificity.

### What is already known on this topic

According to the International Emerging Infections Program and the Influenza Program of the Global Disease Detection network of the US Centers for Disease Control and Prevention, Center for Global Health there is a pressing need to develop global strategies for public health and hRSV prevention.^30^ As it is possible to argue, it is critical to implement surveillance programs because they allow us to estimate the hRSV disease burden, also evaluating the possible under-notification in the real-life and, in future, these programs may allow us to provide data on the real-world vaccine effectiveness.

### What this study adds

This study demonstrates that the presence of fever has a major impact on the surveillance data used to estimate the burden of hRSV disease. Hirve et al.^25^ demonstrated that the presence of fever can considerably affect surveillance data used to estimate the burden of hRSV disease. In this regard, the authors observed that the inclusion of fever reduced sensitivity by 27% in non-hospitalized elderly aged > 65 years.^25^ Some authors have described cases of hRSV infection related to mild fever or even its absence, especially among older patients.^21^^,^^30^ A study conducted in Portugal revealed that the presence of fever was negatively associated with hRSV-positive cases among individuals aged 15–64 and ≥65*.*^13^ Davis et al.^28^ discovered that the SARI case definition has a lower sensitivity and a higher specificity in the over-65 age group for both influenza and hRSV. Korsten et al.^15^ study discovered that the WHO syndromic case definition for ILI dramatically underestimates the confirmed presence of hRSV in community-dwelling elderly by a factor of 9, resulting in a 10-point loss in average sensitivity and specificity when examining older persons. This underestimation is especially troubling given consequences such as death and hospitalization, underscoring the importance of extremely sensitive surveillance in this age range.

Furthermore, the sensitivity of case definitions plays a major role in determining hRSV prevalence, explaining substantial differences among the included studies.

A large part of the articles has a prevalence ranging from 6.25 to 72.54 per 1000 subjects, but it was not possible to establish the mean prevalence across the included studies due to the various case definitions adopted. Moreover, Cui et al.^24^ found observed a remarkably high prevalence of 916.7 positive patients per 1000 persons that, however, was based on a small sample (12 patients, 11 of whom were hRSV positive).

In accordance with the objectives of this review, the findings highlight the importance of adopting a case definition with higher sensitivity, particularly in a territorial setting. Our results indicate that the ARI case definition has higher sensitivity, particularly among non-hospitalized individuals, although the extended SARI definition is more sensitive than both the SARI and modified SARI definitions for hospitalized patients. We recommend for the use of these more detailed case definitions in surveillance programs. Furthermore, we urge a more thorough investigation of hRSV-related symptoms in adults to reduce underreporting of cases and with the aim to find a consistent case definition for hRSV surveillance.^29^

### Limitations of this study

A major problem in generalizing all the abovementioned suggestions is that, to date, hRSV surveillance is marred by the small number of studies in developing countries, difficulties in detecting hRSV cases in the community, and lack of sufficient data on laboratory-confirmed hRSV-associated deaths*.*^30^

A further limitation of this review is the presence of some overlapping case definitions; in fact, several definitions were used simultaneously, with 33% of studies using both ILI and ARI case definitions, and two articles (17%) using both ILI and SARI case definitions. Other limitations to mention are the heterogeneity of patient characteristics, including different age groups, comorbidities, disease severity, as well as the inability to calculate sensitivity, accuracy and specificity in a considerable number of articles. Finally, this review highlights that the use of an accurate case definition is a must in assessing the burden of disease and that the current literature does not, to date, appear to be unambiguously directed.

## Conclusions

This systematic review is intended to provide the scientific community with guidance for the most appropriate future case definition of hRSV; arguably, a different case definition may be needed because of the clinical characteristics that vary across age groups.

In addition, our study shows that there is heterogeneity in the use of different case definitions and that, in the territorial context, the ARI case definition showed higher sensitivity, whereas in hospitalized patients, the extended SARI definition is more sensitive than the SARI and the modified SARI definitions. In conclusion, it is crucial the need for global governing agencies to address this issue urgently in order to implement a case definition that can ensure maximum sensitivity for hRSV surveillance systems, preventing underestimation of the burden of the hRSV and allowing us an accurate estimation of vaccine efficacy in the real world.

## Funding

No funds, grants or other support was received during the preparation of this manuscript.

## Conflict of interest

None declared.

## Ethical approval

None sought.

## Data availability

No new data were created or analyzed in this study.

## Supplementary Material

hRSV-PubMed_fdae066

hRSV-Scopus_fdae066

## Notes

**Emanuele Amodio**, Professor, Medical Doctor, Philosophiæ Doctor

**Miriam Belluzzo**, Medical Doctor

**Dario Genovese**, Medical Doctor

**Martina Palermo**, Medical Doctor

**Vincenzo Pisciotta**, Medical Doctor

**Francesco Vitale**, Professor, Medical Doctor
