# Supplementary material for: What ‘case definition’ for respiratory syncytial virus infection? Results of a systematic literature review to improve surveillance among the adults
Source: J Public Health (Oxf). 2024 May 5;46(3):326–34. doi: 10.1093/pubmed/fdae066 (PMC11914876; doi:10.1093/pubmed/fdae066)
Supplement: hRSV-PubMed_fdae066 [file hrsv-pubmed_fdae066.pdf]

Publication Year	Author	Title	Publication Title	DOI	Abstract Note	Language	Library Catalog
2022	Li, You; Wang, Xin; Blau, Dianna M.; Caballero, Mauricio T.; Feikin, Daniel R.; Gill, Christopher J.; Madhi, Shabir A.; Omer, Saad B.; Simões, Eric A. F.; Campbell, Harry; Pariente, Ana Bermejo; Bardach, Darmaa; Bassat, Quique; Casalegno, Jean-Sebastien; Chakhunashvili, Giorgi; Crawford, Nigel; Danilenko, Daria; Do, Lien Anh Ha; Echavarria, Marcela; Gentile, Angela; Gordon, Aubree; Heikkinen, Terho; Huang, Q. Sue; Jullien, Sophie; Krishnan, Anand; Lopez, Eduardo Luis; Markić, Joško; Mira-Iglesias, Ainara; Moore, Hannah C.; Moyes, Jocelyn; Mwananyanda, Lawrence; Nokes, D. James; Noordeen, Faseeha; Obodai, Evangeline; Palani, Nandhini; Romero, Candice; Salimi, Vahid; Satav, Ashish; Seo, Euri; Shchomak, Zakhar; Singleton, Rosalyn; Stolyarov, Kirill; Stoszek, Sonia K.; von Gottberg, Anne; Wurzel, Danielle; Yoshida, Lay-Myint; Yung, Chee Fu; Zar, Heather J.; Respiratory Virus Global Epidemiology Network; Nair, Harish; RESCEU investigators	Global, regional, and national disease burden estimates of acute lower respiratory infections due to respiratory syncytial virus in children younger than 5 years in 2019: a systematic analysis	Lancet (London, England)	10.1016/S0140-6736(22)00478-0	BACKGROUND: Respiratory syncytial virus (RSV) is the most common cause of acute lower respiratory infection in young children. We previously estimated that in 2015, 33·1 million episodes of RSV-associated acute lower respiratory infection occurred in children aged 0-60 months, resulting in a total of 118 200 deaths worldwide. Since then, several community surveillance studies have been done to obtain a more precise estimation of RSV associated community deaths. We aimed to update RSV-associated acute lower respiratory infection morbidity and mortality at global, regional, and national levels in children aged 0-60 months for 2019, with focus on overall mortality and narrower infant age groups that are targeted by RSV prophylactics in development. METHODS: In this systematic analysis, we expanded our global RSV disease burden dataset by obtaining new data from an updated search for papers published between Jan 1, 2017, and Dec 31, 2020, from MEDLINE, Embase, Global Health, CINAHL, Web of Science, LILACS, OpenGrey, CNKI, Wanfang, and ChongqingVIP. We also included unpublished data from RSV GEN collaborators. Eligible studies reported data for children aged 0-60 months with RSV as primary infection with acute lower respiratory infection in community settings, or acute lower respiratory infection necessitating hospital admission; reported data for at least 12 consecutive months, except for in-hospital case fatality ratio (CFR) or for where RSV seasonality is well-defined; and reported incidence rate, hospital admission rate, RSV positive proportion in acute lower respiratory infection hospital admission, or in-hospital CFR. Studies were excluded if case definition was not clearly defined or not consistently applied, RSV infection was not laboratory confirmed or based on serology alone, or if the report included fewer than 50 cases of acute lower respiratory infection. We applied a generalised linear mixed-effects model (GLMM) to estimate RSV-associated acute lower respiratory infection incidence, hospital admission, and in-hospital mortality both globally and regionally (by country development status and by World Bank Income Classification) in 2019. We estimated country-level RSV-associated acute lower respiratory infection incidence through a risk-factor based model. We developed new models (through GLMM) that incorporated the latest RSV community mortality data for estimating overall RSV mortality. This review was registered in PROSPERO (CRD42021252400). FINDINGS: In addition to 317 studies included in our previous review, we identified and included 113 new eligible studies and unpublished data from 51 studies, for a total of 481 studies. We estimated that globally in 2019, there were 33·0 million RSV-associated acute lower respiratory infection episodes (uncertainty range [UR] 25·4-44·6 million), 3·6 million RSV-associated acute lower respiratory infection hospital admissions (2·9-4·6 million), 26 300 RSV-associated acute lower respiratory infection in-hospital deaths (15 100-49 100), and 101 400 RSV-attributable overall deaths (84 500-125 200) in children aged 0-60 months. In infants aged 0-6 months, we estimated that there were 6·6 million RSV-associated acute lower respiratory infection episodes (4·6-9·7 million), 1·4 million RSV-associated acute lower respiratory infection hospital admissions (1·0-2·0 million), 13 300 RSV-associated acute lower respiratory infection in-hospital deaths (6800-28 100), and 45 700 RSV-attributable overall deaths (38 400-55 900). 2·0% of deaths in children aged 0-60 months (UR 1·6-2·4) and 3·6% of deaths in children aged 28 days to 6 months (3·0-4·4) were attributable to RSV. More than 95% of RSV-associated acute lower respiratory infection episodes and more than 97% of RSV-attributable deaths across all age bands were in low-income and middle-income countries (LMICs). INTERPRETATION: RSV contributes substantially to morbidity and mortality burden globally in children aged 0-60 months, especially during the first 6 months of life and in LMICs. We highlight the striking overall mortality burden of RSV disease worldwide, with one in every 50 deaths in children aged 0-60 months and one in every 28 deaths in children aged 28 days to 6 months attributable to RSV. For every RSV-associated acute lower respiratory infection in-hospital death, we estimate approximately three more deaths attributable to RSV in the community. RSV passive immunisation programmes targeting protection during the first 6 months of life could have a substantial effect on reducing RSV disease burden, although more data are needed to understand the implications of the potential age-shifts in peak RSV burden to older age when these are implemented. FUNDING: EU Innovative Medicines Initiative Respiratory Syncytial Virus Consortium in Europe (RESCEU).	eng	PubMed
2022	Oskarsson, Ymir; Haraldsson, Asgeir; Oddsdottir, Bryndis Hogna I.; Asgeirsdottir, Tinna Laufey; Thors, Valtyr	Clinical and Socioeconomic Burden of Respiratory Syncytial Virus in Iceland	The Pediatric Infectious Disease Journal	10.1097/INF.0000000000003640	INTRODUCTION: Respiratory syncytial virus (RSV), a very common pathogen, causes variable disease severity. In addition to considerable clinical burden on children, their families and healthcare facilities, RSV infections in children also carry significant direct and indirect socioeconomic burden. METHODS: We analyzed data from 5 consecutive RSV seasons (2015-2020) and used virologically confirmed RSV infections and age <5 years as case definition. Clinical information was retrieved from electronic patient records. Costs were estimated by assuming an annual 30% attack rate and a combination of direct medical costs and calculations of societal costs of lost productivity. RESULTS: A total of 716 children younger than 5 years of age had confirmed RSV infection of which 254 needed hospitalizations, most of whom were previously healthy. The median length of admission was 3.6 days and 13 patients needed intensive care. The hospital admission incidence rate was 2.5/1000 children/year, but 9.1 for children younger than 1 years of age. The total annual cost of RSV was estimated at €4.3 million, of which 10% was direct healthcare costs. DISCUSSION: The clinical and socioeconomic disease burden of RSV in Iceland is substantial despite slightly lower hospital admission rates than other high-income countries. The prevention of RSV in young children, either through maternal or infant vaccination, has the potential to decrease both clinical and financial impact of the annual epidemics.	eng	PubMed
2020	Hirve, Siddhivinayak; Crawford, Nigel; Palekar, Rakhee; Zhang, Wenqing; WHO RSV surveillance Group	Clinical characteristics, predictors, and performance of case definition-Interim results from the WHO global respiratory syncytial virus surveillance pilot	Influenza and Other Respiratory Viruses	10.1111/irv.12688	BACKGROUND: The lack of a uniform surveillance case definition poses a challenge to characterize the epidemiology, clinical features, and disease burden of the respiratory syncytial virus (RSV). Global standards for RSV surveillance will inform immunization policy when RSV vaccines become available. METHODS: The WHO RSV surveillance pilot leverages the capacities of the Global Influenza Surveillance and Response System (GISRS). Hospitalized and non-hospitalized medically attended patients of any age were tested for RSV using standardized molecular diagnostics throughout the year in fourteen countries. An extended severe acute respiratory infection (extended SARI) or an acute respiratory infection (ARI) case definition was used that did not require fever as a criterion. RESULTS: Amongst 21 221 patients tested for RSV between January 2017 and September 2018, 15 428 (73%) were hospital admissions. Amongst hospitalized RSV-positive patients, 50% were aged <6 months and 88% <2 years. The percentage of patients testing positive for RSV was 37% in children <6 months and 25% in those aged 6 months to 2 years. Patients with fever were less likely to be RSV positive compared to those without fever (OR 0.74; 95% CI: 0.63-0.86). For infants <6 months, 29% of RSV ARI cases did not have fever. CONCLUSION: Requiring fever in a case definition for RSV lowers the sensitivity to detect cases in young children. Countries should consider ways to leverage the GISRS platform to implement RSV surveillance with an augmented case definition amongst the young pediatric population.	eng	PubMed
2019	Lalani, Karim; Yildirim, Inci; Phadke, Varun K.; Bednarczyk, Robert A.; Omer, Saad B.	Assessment and Validation of Syndromic Case Definitions for Respiratory Syncytial Virus Infections in Young Infants: A Latent Class Analysis	The Pediatric Infectious Disease Journal	10.1097/INF.0000000000002468	BACKGROUND: Respiratory syncytial virus (RSV) is a major cause of pediatric morbidity and mortality worldwide. Standardized case definitions that are applicable to variety of populations are critical for robust surveillance systems to guide decision-making regarding RSV control strategies including vaccine evaluation. Limited data exist on performance of RSV syndromic case definitions among young infants or in high-resource settings. OBJECTIVE: The purpose of this study was to evaluate existing and potential syndromic case definitions for RSV among young infants in an urban, high-income setting using latent class analyses (LCA). METHODS: We used data collected on infants <6 months of age tested for RSV as part of routine clinical care at Children's Healthcare of Atlanta between January 2010 and December 2015. We computed the sensitivity, specificity, positive and negative predictive values of clinical features, existing syndromic case definitions used by the World Health Organization (WHO) and alternative definitions we constructed using LCA to detect RSV infection. RESULTS: Among 565 infants tested for RSV, 161 (28.5%) had laboratory-confirmed RSV infection. Among all case definitions evaluated, the definition developed through LCA (cough plus shortness of breath plus coryza plus wheeze plus poor feeding plus chest in-drawing) was the most specific (95.8%, 95% CI 93.8-97.8) and had the highest positive predictive value (51.4%, 95% CI, 34.9-68.0). WHO-acute respiratory infection (cough or sore throat or shortness of breath or coryza, plus a clinician's judgment that illness is due to infection) was the most sensitive (98.1%, 95% CI, 96.1-100.0; negative predictive value 96.3%, 95% CI 92.2-100.0). CONCLUSIONS: The WHO acute respiratory infection definition could be useful for initial screening for RSV among infants <6 months, whereas our alternative syndromic case definition may serve as the strongest confirmatory case definition in the same population. Appropriate case definitions will vary depending on the content and setting in which they are utilized.	eng	PubMed

2022	Davis, William; Duque, Jazmin; Huang, Q. Sue; Olson, Natalie; Grant, Cameron C.; Newbern, E. Claire; Thompson, Mark; Waite, Ben; Prasad, Namrata; Trenholme, Adrian; Azziz-Baumgartner, Eduardo	Sensitivity and specificity of surveillance case definitions in detection of influenza and respiratory syncytial virus among hospitalized patients, New Zealand, 2012-2016	The Journal of Infection	10.1016/j.jinf.2021.12.012	BACKGROUND: The WHO is exploring the value of adding RSV testing to existing influenza surveillance systems to inform RSV control programs. We evaluate the usefulness of four commonly used influenza surveillance case-definitions for influenza and RSV surveillance. METHODS: SHIVERS, a multi-institutional collaboration, conducted surveillance for influenza and RSV in four New Zealand hospitals. Nurses reviewed admission logs, enrolled patients with suspected acute respiratory infections (ARI), and obtained nasopharyngeal swabs for RT-PCR. We compared the performance characteristics for identifying laboratory-confirmed influenza and RSV severe acute respiratory infection (SARI), defined as persons admitted with measured or reported fever and cough within 10 days of illness, to three other case definitions: 1. reported fever and cough or shortness of breath, 2. cough and shortness of breath, or 3. cough. RESULTS: During April-September 2012-2016, SHIVERS identified 16,055 admissions with ARI; of 6374 cases consented and tested for influenza or RSV, 5437 (85%) had SARI and 937 (15%) did not. SARI had the highest specificity in detecting influenza (40.6%) and RSV (40.8%) but the lowest sensitivity (influenza 78.8%, RSV 60.3%) among patients of all ages. Cough or shortness of breath had the highest sensitivity (influenza 99.3%, RSV 99.9%) but the lowest specificity (influenza 1.6%, RSV 1.9%). SARI sensitivity among children aged <3 months was 60.8% for influenza and 43.6% for RSV-both lower than in other age groups. CONCLUSIONS: While SARI had the highest specificity, its sensitivity was limited, especially among children aged <3 months. Cough or shortness of breath was the most sensitive.	eng	PubMed
2020	Chaw, Pa Saidou; Hua, Lei; Cunningham, Steve; Campbell, Harry; Mikolajczyk, Rafael; Nair, Harish; RESCEU Investigators	Respiratory Syncytial Virus-Associated Acute Lower Respiratory Infections in Children With Bronchopulmonary Dysplasia: Systematic Review and Meta-Analysis	The Journal of Infectious Diseases	10.1093/infdis/jiz492	BACKGROUND: Respiratory syncytial virus (RSV) is among the most important causes of acute lower respiratory tract infection (ALRI) in young children. We assessed the severity of RSV-ALRI in children less than 5 years old with bronchopulmonary dysplasia (BPD). METHODS: We searched for studies using EMBASE, Global Health, and MEDLINE. We assessed hospitalization risk, intensive care unit (ICU) admission, need for oxygen supplementation and mechanical ventilation, and in-hospital case fatality (hCFR) among children with BPD compared with those without (non-BPD). We compared the (1) length of hospital stay (LOS) and (2) duration of oxygen supplementation and mechanical ventilation between the groups. RESULTS: Twenty-nine studies fulfilled our inclusion criteria. The case definition for BPD varied substantially in the included studies. Risks were higher among children with BPD compared with non-BPD: RSV hospitalization (odds ratio [OR], 2.6; 95% confidence interval [CI], 1.7-4.2; P < .001), ICU admission (OR, 2.9; 95% CI, 2.3-3.5; P < .001), need for oxygen supplementation (OR, 4.2; 95% CI, .5-33.7; P = .175) and mechanical ventilation (OR, 8.2; 95% CI, 7.6-8.9; P < .001), and hCFR (OR, 12.8; 95% CI, 9.4-17.3; P < .001). Median LOS (range) was 7.2 days (4-23) (BPD) compared with 2.5 days (1-30) (non-BPD). Median duration of oxygen supplementation (range) was 5.5 days (0-21) (BPD) compared with 2.0 days (0-26) (non-BPD). The duration of mechanical ventilation was more often longer (>6 days) in those with BPD compared with non-BPD (OR, 11.9; 95% CI, 1.4-100; P = .02). CONCLUSIONS: The risk of severe RSV disease is considerably higher among children with BPD. There is an urgent need to establish standardized BPD case definitions, review the RSV prophylaxis guidelines, and encourage more specific studies on RSV infection in BPD patients, including vaccine development and RSV-specific treatment.	eng	PubMed
2019	Omer, Saad B.; Bednarczyk, Robert; Kazi, Momin; Guterman, L. Beryl; Aziz, Fatima; Allen, Kristen E.; Yildirim, Inci; Ali, S. Asad	Assessment and Validation of Syndromic Case Definitions for Respiratory Syncytial Virus Testing in a Low Resource Population	The Pediatric Infectious Disease Journal	10.1097/INF.0000000000002159	Standardized case definitions are needed in decision-making regarding respiratory syncytial virus control strategies, including vaccine evaluation. A syndromic case definition comprising of "wheeze or apnea or cyanosis" could be useful for community-based surveillance of moderate respiratory syncytial virus infection among young infants particularly in resource-limited settings. However, this definition showed modest specificity (29.2%-49.6%), indicating that community-based surveillance may need augmentation with other data.	eng	PubMed
2019	Chavez, Dabeyva; Gonzales-Armayo, Vicente; Mendoza, Elvis; Palekar, Rakhee; Rivera, Rosario; Rodriguez, Angel; Salazar, Claudia; Veizaga, Angel; Añez, Arletta	Estimation of influenza and respiratory syncytial virus hospitalizations using sentinel surveillance data-La Paz, Bolivia. 2012-2017	Influenza and Other Respiratory Viruses	10.1111/irv.12663	OBJECTIVE: The objective was to estimate the number of hospitalizations associated with influenza and RSV using data from severe acute respiratory infection (SARI) sentinel surveillance from El Alto-La Paz. Bolivia. METHODS: All persons who met the case definition for SARI at one sentinel hospital had a clinical sample collected and analyzed by rRT-PCR for influenza and by indirect immunofluorescence for RSV. The SARI-influenza and SARI-RSV case counts were stratified by six age groups. The proportion of cases captured in the sentinel hospital in relation to the non-sentinel hospitals of area was multiplied by the age-specific census population, to build the denominators. The annual incidence and a 95% confidence interval (CI) were estimated. RESULTS: During 2012-2017, n = 2606 SARI cases were reported (average incidence 120/100 000 inhabitants [95% CI: 116-124]); the average incidence of influenza-associated SARI hospitalization was 15.3/100 000 (95% CI: 14.1-16.7), and the average incidence of RSV-associated SARI hospitalization was 9/100 000 inhabitants (95% CI: 8.1-10.1). The highest incidence of influenza was among those less than one year of age (average 174.7/100 000 [range: 89.1-299.5]), followed by those one to four years of age (average 51.8/100 000 [range: 19.8-115.4]) and then those 65 years of age and older (average 47.7/100 000 [range: 18.8-117]). For RSV, the highest incidence was highest among those less than one year of age (231/100 000 [range: 119.9-322.9]). CONCLUSION: Influenza and RSV represent major causes of hospitalization in La Paz, Bolivia-with the highest burden among children under one year of age. Our estimates support current prevention strategies in this age group.	eng	PubMed
2020	Bourgeois, Marc; Ausselet, Nathalie; Gerard, Veronique; de Canniere, Louis; Scius, Nathan; Michaux, Isabelle; Huang, Te-Din; Bogaerts, Pierre; Vandamme, Charlotte; Bihin, Benoît; Delaere, Benedicte	Severe influenza/respiratory syncytial virus infections and hospital antimicrobial stewardship opportunities: impact of a 4-year surveillance including molecular diagnosis	Infection Control and Hospital Epidemiology	10.1017/ice.2020.260	OBJECTIVE: To assess the prevalence of influenza and respiratory syncytial virus (RSV) in adults hospitalized for a respiratory infection in the winter months and to evaluate the impact of a viral diagnosis on empirical antimicrobial management (antibiotics and antivirals). DESIGN: Observational cohort study. SETTING: Acute-care university hospital. PATIENTS: The study included 963 adult patients hospitalized over a 4-year surveillance period. METHODS: Annual surveillance timelines were defined according to epidemiological criteria related to the circulation of RSV and influenza viruses in the general population. Patients were screened following a severe acute respiratory infection (SARI) case definition at the emergency department and were enrolled for molecular assay targeting influenza/RSV viruses after oral informed consent. Epidemiological and clinical data were recorded prospectively, microbiological investigations, antimicrobial management, and outcome data were reviewed retrospectively. RESULTS: An influenza or RSV virus was documented in 316 of 963 patients (33%). Optimization of antimicrobial management (AM) was achieved in 162 of 265 patients (61%) with a positive viral diagnosis and no bacterial infection at admission (AM treatment not initiated, n = 111; discontinued, n = 51). In contrast, only 128 of 462 patients (28%) with negative microbiological investigations did not have AM treatment initiated (n = 116) or had such treatment discontinued (n = 12). Early, targeted antiviral treatment was prescribed in 235 of 253 patients (93%) confirmed with influenza. Epidemiological, clinical, and outcome data were similar in both groups. CONCLUSION: Epidemiological surveillance associated with influenza/RSV molecular diagnosis in adults hospitalized for severe winter respiratory infections dramatically enhanced antimicrobial management.	eng	PubMed

2022	Koul, Parvaiz A.; Saha, Siddhartha; Kaul, Kaisar A.; Mir, Hyder; Potdar, Varsha; Chadha, Mandeep; Iuliano, Danielle; Lafond, Kathryn E.; Lal, Renu B.; Krishnan, Anand	Respiratory syncytial virus among children hospitalized with severe acute respiratory infection in Kashmir, a temperate region in northern India	Journal of Global Health	10.7189/jogh.12.04050	BACKGROUND: Severe acute respiratory infections (SARI) are a leading cause of hospitalizations in children, especially due to viral pathogens. We studied the prevalence of respiratory viruses among children aged <5 years hospitalized with severe acute respiratory infections (SARI) in Kashmir, India. METHODS: We conducted a prospective observational study in two tertiary care hospitals from October 2013 to September 2014, systematically enrolling two children aged <5 years with SARI per day. We defined SARI as history of fever or measured fever (≥38°C) and cough with onset in the last 7 days requiring hospitalization for children aged 3-59 months and as physician-diagnosed acute lower respiratory infection for children aged <3 months. Trained study staff screened children within 24 hours of hospitalization for SARI and collected clinical data and nasopharyngeal swabs from enrolled participants. We tested for respiratory syncytial virus (RSV) A and B, influenza viruses, rhinoviruses (HRV)/enteroviruses, adenovirus (AdV), bocavirus (BoV), human metapneumovirus (hMPV) A and B, coronaviruses (OC43, NL65, C229E), and parainfluenza viruses (PIV) 1, 2, 3 and 4 using standardized duplex real-time polymerase chain reaction. RESULTS: Among 4548 respiratory illness admissions screened from October 2013 to September 2014, 1026 met the SARI case definition, and 412 were enrolled (ages = 5 days to 58 months; median = 12 months). Among enrolees, 256 (62%) were positive for any virus; RSV was the most commonly detected (n = 118, 29%) followed by HRV/enteroviruses (n = 88, 21%), PIVs (n = 31, 8%), influenza viruses (n = 18, 4%), BoV (n = 15, 4%), coronaviruses (n = 16, 4%), AdV (n = 14, 3%), and hMPV (n = 9, 2%). Fifty-four children had evidence of virus co-detection. Influenza-associated SARI was more common among children aged 1-5 years (14/18, 78%) while most RSV detections occurred in children <12 months (83/118, 70%). Of the RSV viruses typed (n = 116), the majority were type B (94, 80%). Phylogenetic analysis of G gene of RSV showed circulation of the BA9 genotype with 60bp nucleotide duplication. CONCLUSIONS: Respiratory viruses, especially RSV, contributed to a substantial proportion of SARI hospitalizations among children <5 years in north India. These data can help guide clinicians on appropriate treatment and prevention strategies.	eng	PubMed
2020	Resch, Bernhard; Puchas, Claudia; Resch, Elisabeth; Urlesberger, Berndt	Epidemiology of Respiratory Syncytial Virus-related Hospitalizations and the Influence of Viral Coinfections in Southern Austria in a 7-year Period	The Pediatric Infectious Disease Journal	10.1097/INF.0000000000002494	OBJECTIVE: The aim of this study was to determine the respiratory syncytial virus (RSV) epidemiology and to analyze the influence of risk factors and coinfections over the last years. METHODS: Retrospectively all infants, children and adolescents hospitalized due to respiratory disease with positive RSV test [hospitalized for RSV infection (RSV-H)] between January 1, 2009, and December 31, 2015, at a tertiary care center in the southern part of Austria were included for analysis. Patients were all identified by a search via International Classification of Diseases and Related Health Problems, 10th Edition codes, and all medical data were collected from the local electronic databases called openMedocs. RSV tests had to prove true infection case definition. RESULTS: During a 7-year study period, 745 infants, children and adolescents exhibited RSV-H. Main diagnosis was bronchiolitis (70%). Nearly half of all cases (44%) were born during the first half of the RSV season (November-January), and seasonal peak of RSV-H was in January. Predominant underlying condition was history of prematurity in 15% followed by neurologic impairment (3.5%) and hemodynamically significant congenital heart disease (2.95%). Age ≤2 months and underlying conditions/morbidities were associated with more severe disease. The majority of cases (96%) had an age below 24 months, and 91% below 12 months. Viral coinfection (most common influenza virus, adenovirus and rhinovirus) was diagnosed in 37 cases (5%) resulting in a more severe course of disease. Main risk factors of coinfection were siblings and crowding. Mortality was 0.27% (2/745). Both children had coinfection with influenza A virus and were multihandicapped (15 and 20 years of age, respectively). CONCLUSIONS: Prematurity and underlying morbidities play a marked role in RSV-H. Viral coinfections aggravated disease with death in 2 multihandicapped adolescents.	eng	PubMed
2022	Korsten, Koos; Adriaenssens, Niels; Coenen, Samuel; Butler, Chris C.; Verheij, Theo J. M.; Bont, Louis J.; Wildenbeest, Joanne G.; RESCEU Investigators	World Health Organization Influenza-Like Illness Underestimates the Burden of Respiratory Syncytial Virus Infection in Community-Dwelling Older Adults	The Journal of Infectious Diseases	10.1093/infdis/jiab452	BACKGROUND: Respiratory syncytial virus (RSV) surveillance is heavily dependent on the influenza-like illness (ILI) case definition from the World Health Organization (WHO). Because ILI includes fever in its syndromic case definition, its ability to accurately identify acute respiratory tract infections (ARTI) caused by RSV in older adults is uncertain. METHODS: The accuracy of the WHO ILI and a modified ILI (requiring only self-reported fever) case definitions in identifying patients with PCR-confirmed RSV-ARTI was evaluated in community-dwelling older adults (≥60 years) from the prospective European RESCEU cohort study. RESULTS: Among 1040 participants, 750 ARTI episodes were analyzed including 36 confirmed RSV-ARTI. Due to a general lack of fever, sensitivity for RSV-ARTI was 33% for modified ILI and 11% for ILI. The area under the curve for both ILI definitions was 0.52 indicating poor discrimination for RSV. RSV-ARTI could not be distinguished from all other ARTI based on clinical symptoms. CONCLUSIONS: The use of ILI underestimated the occurrence of RSV-ARTI in community-dwelling older adults up to 9-fold (11% sensitivity). Because worldwide RSV surveillance depends largely on ILI, there is an urgent need for a better approach to measure the occurrence of RSV disease and the impact of future RSV vaccine introduction. Clinical Trials Registration. NCT03621930.	eng	PubMed
2020	Subissi, Lorenzo; Bossuyt, Nathalie; Reynders, Marijke; Gérard, Michèle; Dauby, Nicolas; Bourgeois, Marc; Delaere, Bénédicte; Quoilin, Sophie; Van Gucht, Steven; Thomas, Isabelle; Barbezange, Cyril	Capturing respiratory syncytial virus season in Belgium using the influenza severe acute respiratory infection surveillance network, season 2018/19	Euro Surveillance: Bulletin Europeen Sur Les Maladies Transmissibles = European Communicable Disease Bulletin	10.2807/1560-7917.ES.2020.25.39.1900627	BackgroundRespiratory syncytial virus (RSV) is a common cause of severe respiratory illness in young children (< 5 years old) and older adults (≥ 65 years old) leading the World Health Organization (WHO) to recommend the implementation of a dedicated surveillance in countries.AimWe tested the capacity of the severe acute respiratory infection (SARI) hospital network to contribute to RSV surveillance in Belgium.MethodsDuring the 2018/19 influenza season, we started the SARI surveillance for influenza in Belgium in week 40, earlier than in the past, to follow RSV activity, which usually precedes influenza virus circulation. While the WHO SARI case definition for influenza normally used by the SARI hospital network was employed, flexibility over the fever criterion was allowed, so patients without fever but meeting the other case definition criteria could be included in the surveillance.ResultsBetween weeks 40 2018 and 2 2019, we received 508 samples from SARI patients. We found an overall RSV detection rate of 62.4% (317/508), with rates varying depending on the age group: 77.6% in children aged < 5 years (253/326) and 34.4% in adults aged ≥ 65 years (44/128). Over 90% of the RSV-positive samples also positive for another tested respiratory virus (80/85) were from children aged < 5 years. Differences were also noted between age groups for symptoms, comorbidities and complications.ConclusionWith only marginal modifications in the case definition and the period of surveillance, the Belgian SARI network would be able to substantially contribute to RSV surveillance and burden evaluation in children and older adults, the two groups of particular interest for WHO.	eng	PubMed
2019	Sáez-López, Emma; Pechirra, Pedro; Costa, Inês; Cristóvão, Paula; Conde, Patrícia; Machado, Ausenda; Rodrigues, Ana Paula; Guiomar, Raquel	Performance of surveillance case definitions for respiratory syncytial virus infections through the sentinel influenza surveillance system, Portugal, 2010 to 2018	Euro Surveillance: Bulletin Europeen Sur Les Maladies Transmissibles = European Communicable Disease Bulletin	10.2807/1560-7917.ES.2019.24.45.1900140	BackgroundWell-established influenza surveillance systems (ISS) can be used for respiratory syncytial virus (RSV) surveillance. In Portugal, RSV cases are detected through the ISS using the European Union (EU) influenza-like illness (ILI) case definition.AimTo investigate clinical predictors for RSV infection and how three case definitions (EU ILI, a modified EU acute respiratory infection, and one respiratory symptom) performed in detecting RSV infections in Portugal.MethodsThis observational retrospective study used epidemiological and laboratory surveillance data (October 2010-May 2018). Associations between clinical characteristics and RSV detection were analysed using logistic regression. Accuracy of case definitions was assessed through sensitivity, specificity, and area under the receiver operating characteristic curve (AUC). A 0.05 significance level was accepted.ResultsThe study involved 6,523 persons, including 190 (2.9%) RSV cases. Among 183 cases with age information, RSV infection was significantly more frequent among individuals < 5 years (n = 23; 12.6%) and ≥ 65 years (n = 45; 24.6%) compared with other age groups (p < 0.0001). Cough (odds ratio (OR): 2.4; 95% confidence interval (CI): 1.2-6.5) was the best RSV-infection predictor considering all age groups, while shortness of breath was particularly associated with RSV-positivity among ≤ 14 year olds (OR: 6.7; 95% CI: 2.6-17.4 for 0-4 year olds and OR: 6.7; 95% CI: 1.5-28.8 for 5-14 year olds). Systemic symptoms were significantly associated with RSV-negative and influenza-positive cases. None of the case definitions were suitable to detect RSV infections (AUC = 0.51).ConclusionTo avoid underestimating the RSV disease burden, RSV surveillance within the Portuguese sentinel ISS would require a more sensitive case definition than ILI and, even a different case definition according to age.	eng	PubMed
2020	Wilmont, Sibyl; Neu, Natalie; Hill-Ricciuti, Alexandra; Alba, Luis; Prill, Mila M.; Whitaker, Brett; Garg, Shikha; Stone, Nimalie D.; Lu, Xiaoyan; Kim, Lindsay; Gerber, Susan I.; Larson, Elaine; Saiman, Lisa	Active surveillance for acute respiratory infections among pediatric long-term care facility staff	American Journal of Infection Control	10.1016/j.ajic.2020.06.190	BACKGROUND: Transmission of respiratory viruses between staff and residents of pediatric long-term care facilities (pLTCFs) can occur. We assessed the feasibility of using text or email messages to perform surveillance for acute respiratory infections (ARIs) among staff. METHODS: From December 7, 2016 to May 7, 2017, 50 staff participants from 2 pLTCFs received weekly text or email requests to report the presence or absence of ARI symptoms. Those who fulfilled the ARI case definition (≥2 symptoms) had respiratory specimens collected to detect viruses by reverse transcriptase polymerase chain reaction assays. Pre- and postsurveillance respiratory specimens were collected to assess subclinical viral shedding. RESULTS: The response rate to weekly electronic messages was 93%. Twenty-one ARIs reported from 20 (40%) participants fulfilled the case definition. Respiratory viruses were detected in 29% (5/17) of specimens collected at symptom onset (influenza B, respiratory syncytial virus, coronavirus [CoV] 229E, rhinovirus [RV], and dual detection of CoV OC43 and bocavirus). Four participants had positive presurveillance (4 RV), and 6 had positive postsurveillance specimens (3 RV, 2 CoV NL63, and 1 adenovirus). CONCLUSIONS: Electronic messaging to conduct ARI surveillance among pLTCF staff was feasible.	eng	PubMed

2019	Rha, Brian; Dahl, Rebecca M.; Moyes, Jocelyn; Binder, Alison M.; Tempia, Stefano; Walaza, Sibongile; Bi, Daoling; Groome, Michelle J.; Variava, Ebrahim; Naby, Fathima; Kahn, Kathleen; Treurnicht, Florette; Cohen, Adam L.; Gerber, Susan I.; Madhi, Shabir A.; Cohen, Cheryl	Performance of Surveillance Case Definitions in Detecting Respiratory Syncytial Virus Infection Among Young Children Hospitalized With Severe Respiratory Illness-South Africa, 2009-2014	Journal of the Pediatric Infectious Diseases Society	10.1093/jpids/piy055	BACKGROUND: Respiratory syncytial virus (RSV) is a leading cause of acute lower respiratory tract infection (ALRTI) in young children, but data on surveillance case definition performance in estimating burdens have been limited. METHODS: We enrolled children aged <5 years hospitalized for ALRTI (or neonatal sepsis in young infants) through active prospective surveillance at 5 sentinel hospitals in South Africa and collected nasopharyngeal aspirates from them for RSV molecular diagnostic testing between 2009 and 2014. Clinical data were used to characterize RSV disease and retrospectively evaluate the performance of respiratory illness case definitions (including the World Health Organization definition for severe acute respiratory infection [SARI]) in identifying hospitalized children with laboratory-confirmed RSV according to age group (<3, 3-5, 6-11, 12-23, and 24-59 months). RESULTS: Of 9969 hospitalized children, 2723 (27%) tested positive for RSV. Signs and symptoms in RSV-positive children varied according to age; fever was less likely to occur in children aged <3 months (57%; odds ratio [OR], 0.8 [95% CI, 0.7-0.9]) but more likely in those aged ≥12 months (82%; OR, 1.7-1.9) than RSV-negative children. The sensitivity (range, 55%-81%) and specificity (range, 27%-54%) of the SARI case definition to identify hospitalized RSV-positive children varied according to age; the lowest sensitivity was for infants aged <6 months. Using SARI as the case definition would have missed 36% of RSV-positive children aged <5 years and 49% of those aged <3 months; removing the fever requirement from the definition recovered most missed cases. CONCLUSION: Including fever in the SARI case definition lowers the sensitivity for RSV case detection among young children hospitalized with an ALRTI and likely underestimates its burden.	eng	PubMed
2022	Ramay, Brooke M.; Jara, Jorge; Moreno, Maria Purificación; Lupo, Patrizia; Serrano, Carlos; Alvis, Juan P.; Arriola, C. Sofia; Veguilla, Vic; Kaydos-Daniels, S. Cornelia	Self-medication and ILI etiologies among individuals presenting at pharmacies with influenza-like illness: Guatemala City, 2018 influenza season	BMC public health	10.1186/s12889-022-13962-8	OBJECTIVES: We aimed to characterize the proportion of clients presenting to community pharmacies with influenza-like illness (ILI) and the severity of their illness; the proportion with detectable influenza A, influenza B, and other pathogens (i.e., parainfluenza I, II, and III, adenovirus, respiratory syncytial virus, human metapneumovirus); and to describe their self-medication practices. METHODS: A cross-sectional study was conducted in six pharmacies in Guatemala City. Study personnel collected nasopharyngeal and oropharyngeal swabs from participants who met the ILI case definition and who were self-medicating for the current episode. Participants were tested for influenza A and B and other pathogens using real-time RT-PCR. Participants' ILI-associated self-medication practices were documented using a questionnaire. RESULTS: Of all patients entering the pharmacy during peak hours who responded to a screening survey (n = 18,016) 6% (n = 1029) self-reported ILI symptoms, of which 45% (n = 470/1029) met the study case definition of ILI. Thirty-one percent (148/470) met inclusion criteria, of which 87% (130/148) accepted participation and were enrolled in the study. Among 130 participants, nearly half tested positive for viral infection (n = 55, 42.3%) and belonged to groups at low risk for complications from influenza. The prevalence of influenza A was 29% (n = 35). Thirteen percent of the study population (n = 17) tested positive for a respiratory virus other than influenza. Sixty-four percent of participants (n = 83) reported interest in receiving influenza vaccination if it were to become available in the pharmacy. Medications purchased included symptom-relieving multi-ingredient cold medications (n = 43/100, 43%), nonsteroidal anti-inflammatory drugs (n = 23, 23%), and antibiotics (n = 16, 16%). Antibiotic use was essentially equal among antibiotic users regardless of viral status. The broad-spectrum antibiotics ceftriaxone and azithromycin were the most common antibiotics purchased. CONCLUSIONS: During a typical influenza season, a relatively low proportion of all pharmacy visitors were experiencing influenza symptoms. A high proportion of clients presenting to pharmacies with ILI tested positive for a respiratory virus. Programs that guide appropriate use of antibiotics in this population are needed and become increasingly important during pandemics caused by respiratory viral pathogens.	eng	PubMed
2022	von Mollendorf, Claire; Berger, Daria; Gwee, Amanda; Duke, Trevor; Graham, Stephen M.; Russell, Fiona M.; Mulholland, E. Kim; ARI review group	Aetiology of childhood pneumonia in low- and middle-income countries in the era of vaccination: a systematic review	Journal of Global Health	10.7189/jogh.12.10009	BACKGROUND: This systematic review aimed to describe common aetiologies of severe and non-severe community acquired pneumonia among children aged 1 month to 9 years in low- and middle-income countries. METHODS: We searched the MEDLINE, EMBASE, and PubMed online databases for studies published from January 2010 to August 30, 2020. We included studies on acute community-acquired pneumonia or acute lower respiratory tract infection with ≥1 year of continuous data collection; clear consistent case definition for pneumonia; >1 specimen type (except empyema studies where only pleural fluid was required); testing for >1 pathogen including both viruses and bacteria. Two researchers reviewed the studies independently. Results were presented as a narrative summary. Quality of evidence was assessed with the Quality Assessment Tool for Quantitative Studies. The study was registered on PROSPERO [CRD42020206830]. RESULTS: We screened 5184 records; 1305 duplicates were removed. The remaining 3879 titles and abstracts were screened. Of these, 557 articles were identified for full-text review, and 55 met the inclusion criteria - 10 case-control studies, three post-mortem studies, 11 surveillance studies, eight cohort studies, five cross-sectional studies, 12 studies with another design and six studies that included patients with pleural effusions or empyema. Studies which described disease by severity showed higher bacterial detection (Streptococcus pneumoniae, Staphylococcus aureus) in severe vs non-severe cases. The most common virus causing severe disease was respiratory syncytial virus (RSV). Pathogens varied by age, with RSV and adenovirus more common in younger children. Influenza and atypical bacteria were more common in children 5-14 years than younger children. Malnourished and HIV-infected children had higher rates of pneumonia due to bacteria or tuberculosis. CONCLUSIONS: Several viral and bacterial pathogens were identified as important targets for prevention and treatment. Bacterial pathogens remain an important cause of moderate to severe disease, particularly in children with comorbidities despite widespread PCV and Hib vaccination.	eng	PubMed
2022	Bimouhen, Abderrahman; Regragui, Zakia; El Falaki, Fatima; Ihazmade, Hassan; Benkerroum, Samira; Cherkaoui, Imad; Rguig, Ahmed; Ezzine, Hind; Benamar, Touria; Triki, Soumia; Bakri, Youssef; Oumzil, Hicham	Viral aetiology of influenza-like illnesses and severe acute respiratory illnesses in Morocco, September 2014 to December 2016	Journal of Global Health	10.7189/jogh.12.04062	BACKGROUND: There is a scarcity of information on the viral aetiology of influenza-like illness (ILI) and severe acute respiratory infection (SARI) among patients in Morocco. METHODS: From September 2014 to December 2016, we prospectively enrolled inpatients and outpatients from all age groups meeting the World Health Organization (WHO) case definition for ILI and SARI from 59 sentinel sites. The specimens were tested using real-time monoplex reverse-transcription polymerase chain reaction method for detecting 16 relevant respiratory viruses. RESULTS: At least one respiratory virus was detected in 1423 (70.8%) of 2009 specimens. Influenza viruses were the most common, detected in 612 (30.4%) of processed samples, followed by respiratory syncytial virus (RSV) in 359 (17.9%), human rhinovirus (HRV) in 263 (13.1%), adenovirus (HAdV) in 124 (6.2%), parainfluenza viruses (HPIV) in 107 (5.3%), coronaviruses (HCoV) in 94 (4.7%), human bocavirus (HBoV) in 92 (4.6%), and human metapneumovirus (HMPV) in 74 (3.7%). From 770 samples from children under 5 years old, RSV (288, 36.6%), influenza viruses (106, 13.8%), HRV (96, 12.5%) and HAdV (91, 11.8%) were most prevalent. Among 955 samples from adults, Influenza viruses (506, 53.0%), and HRV (167, 17.5%) were most often detected. Co-infections were found in 268 (18.8%) of 1423 positive specimens, and most (60.4%) were in children under 5 years of age. While influenza viruses, RSV, and HMPV had a defined period of circulation, the other viruses did not display clear seasonal patterns. CONCLUSIONS: We found that RSV was predominant among SARI cases in Morocco, particularly in children under 5 years of age. Our results are in line with reported data from other parts of the world, stating that RSV is the leading cause of lower respiratory tract infections in infants and young children.	eng	PubMed
2016	Nyawanda, Bryan O.; Mott, Joshua A.; Njuguna, Henry N.; Mayieka, Lilian; Khagayi, Sammy; Onkoba, Reuben; Makokha, Caroline; Otieno, Nancy A.; Bigogo, Godfrey M.; Katz, Mark A.; Feikin, Daniel R.; Verani, Jennifer R.	Evaluation of case definitions to detect respiratory syncytial virus infection in hospitalized children below 5 years in Rural Western Kenya, 2009-2013	BMC infectious diseases	10.1186/s12879-016-1532-0	BACKGROUND: In order to better understand respiratory syncytial virus (RSV) epidemiology and burden in tropical Africa, optimal case definitions for detection of RSV cases need to be identified. METHODS: We used data collected between September 2009 - August 2013 from children aged <5 years hospitalized with acute respiratory Illness at Siaya County Referral Hospital. We evaluated the sensitivity, specificity, positive predictive value (PPV) and negative predictive value (NPV) of individual signs, symptoms and standard respiratory disease case definitions (severe acute respiratory illness [SARI]; hospitalized influenza-like illness [hILI]; integrated management of childhood illness [IMCI] pneumonia) to detect laboratory-confirmed RSV infection. We also evaluated an alternative case definition of cough or difficulty breathing plus hypoxia, in-drawing, or wheeze. RESULTS: Among 4714 children hospitalized with ARI, 3810 (81 %) were tested for RSV; and 470 (12 %) were positive. Among individual signs and symptoms, cough alone had the highest sensitivity to detect laboratory-confirmed RSV [96 %, 95 % CI (95-98)]. Hypoxia, wheezing, stridor, nasal flaring and chest wall in-drawing had sensitivities ranging from 8 to 31 %, but had specificities >75 %. Of the standard respiratory case definitions, SARI had the highest sensitivity [83 %, 95 % CI (79-86)] whereas IMCI severe pneumonia had the highest specificity [91 %, 95 % CI (90-92)]. The alternative case definition (cough or difficulty breathing plus hypoxia, in-drawing, or wheeze) had a sensitivity of [55 %, 95 % CI (50-59)] and a specificity of [60 %, 95 % CI (59-62)]. The PPV for all case definitions and individual signs/symptoms ranged from 11 to 20 % while the negative predictive values were >87 %. When we stratified by age <1 year and 1- < 5 years, difficulty breathing, severe pneumonia and the alternative case definition were more sensitive in children aged <1 year [70 % vs. 54 %, p < 0.01], [19 % vs. 11 %, p = 0.01] and [66 % vs. 43 %, p < 0.01] respectively, while non-severe pneumonia was more sensitive [14 % vs. 26 %, p < 0.01] among children aged 1- < 5 years. CONCLUSION: The sensitivity and specificity of different commonly used case definitions for detecting laboratory-confirmed RSV cases varied widely, while the positive predictive value was consistently low. Optimal choice of case definition will depend upon study context and research objectives.	eng	PubMed

2020	Vanderburg, Sky; Wijayaratne, Gaya; Danthanarayana, Nayomi; Jayamaha, Jude; Piyasiri, Bhagya; Halloluwa, Chathurangi; Sheng, Tianchen; Amarasena, Sujeewa; Kurukulasooriya, Ruvini; Nicholson, Bradly P.; Peiris, Joseph S. M.; Gray, Gregory C.; Gunasena, Sunethra; Nagahawatte, Ajith; Bodinayake, Champica K.; Woods, Christopher W.; Devasiri, Vasantha; Tillekeratne, L. Gayani	Outbreak of severe acute respiratory infection in Southern Province, Sri Lanka in 2018: a cross-sectional study	BMJ open	10.1136/bmjopen-2020-040612	OBJECTIVES: To determine aetiology of illness among children and adults presenting during outbreak of severe respiratory illness in Southern Province, Sri Lanka, in 2018. DESIGN: Prospective, cross-sectional study. SETTING: 1600-bed, public, tertiary care hospital in Southern Province, Sri Lanka. PARTICIPANTS: 410 consecutive patients, including 371 children and 39 adults, who were admitted with suspected viral pneumonia (passive surveillance) or who met case definition for acute respiratory illness (active surveillance) in May to June 2018. RESULTS: We found that cocirculation of influenza A (22.6% of cases), respiratory syncytial virus (27.8%) and adenovirus (AdV) (30.7%; type B3) was responsible for the outbreak. Mortality was noted in 4.5% of paediatric cases identified during active surveillance. Virus type and viral coinfection were not significantly associated with mortality. CONCLUSIONS: This is the first report of intense cocirculation of multiple respiratory viruses as a cause of an outbreak of severe acute respiratory illness in Sri Lanka, and the first time that AdV has been documented as a cause of a respiratory outbreak in the country. Our results emphasise the need for continued vigilance in surveying for known and emerging respiratory viruses in the tropics.	eng	PubMed
2017	Chughtai, A. A.; Wang, Q.; Dung, T. C.; Macintyre, C. R.	The presence of fever in adults with influenza and other viral respiratory infections	Epidemiology and Infection	10.1017/S0950268816002181	We compared the rates of fever in adult subjects with laboratory-confirmed influenza and other respiratory viruses and examined the factors that predict fever in adults. Symptom data on 158 healthcare workers (HCWs) with a laboratory-confirmed respiratory virus infection were collected using standardized data collection forms from three separate studies. Overall, the rate of fever in confirmed viral respiratory infections in adult HCWs was 23·4% (37/158). Rates varied by virus: human rhinovirus (25·3%, 19/75), influenza A virus (30%, 3/10), coronavirus (28·6%, 2/7), human metapneumovirus (28·6%, 2/7), respiratory syncytial virus (14·3%, 4/28) and parainfluenza virus (8·3%, 1/12). Smoking [relative risk (RR) 4·65, 95% confidence interval (CI) 1·33-16·25] and co-infection with two or more viruses (RR 4·19, 95% CI 1·21-14·52) were significant predictors of fever. Fever is less common in adults with confirmed viral respiratory infections, including influenza, than described in children. More than 75% of adults with a viral respiratory infection do not have fever, which is an important finding for clinical triage of adult patients with respiratory infections. The accepted definition of 'influenza-like illness' includes fever and may be insensitive for surveillance when high case-finding is required. A more sensitive case definition could be used to identify adult cases, particularly in event of an emerging viral infection.	eng	PubMed
2015	Saha, Siddhartha; Pandey, Bharti Gaur; Choudekar, Avinash; Krishnan, Anand; Gerber, Susan I.; Rai, Sanjay K.; Singh, Pratibha; Chadha, Mandeep; Lal, Renu B.; Broor, Shobha	Evaluation of case definitions for estimation of respiratory syncytial virus associated hospitalizations among children in a rural community of northern India	Journal of Global Health	10.7189/jogh.05.020419	BACKGROUND: The burden estimation studies for respiratory syncytial virus (RSV) have been based on varied case definitions, including case-definitions designed for influenza surveillance systems. We used all medical admissions among children aged 0-59 months to study the effect of case definitions on estimation of RSV-associated hospitalizations rates. METHODS: The hospital-based daily surveillance enrolled children aged 0-59 months admitted with acute medical conditions from July 2009-December 2012, from a well-defined rural population in Ballabgarh in northern India. All study participants were examined and nasal and throat swabs taken for testing by real-time polymerase chain reaction (RT-PCR) for RSV and influenza virus. Clinical data were used to retrospectively evaluate World Health Organization (WHO) case definitions (2011) commonly used for surveillance of respiratory pathogens, ie, acute respiratory illness (WHO-ARI), severe ARI (SARI) and influenza-like illness (ILI), for determination of RSV-associated hospitalization. RSV-associated hospitalization rates adjusted for admissions at non-study hospitals were calculated. FINDINGS: Out of 505 children enrolled, 82 (16.2%) tested positive for RSV. Annual incidence rates of RSV-associated hospitalization per 1000 children were highest among infants aged 0-5 months (15.2; 95% confidence interval (CI) 8.3-26.8), followed by ages 6-23 months (5.3, 95% CI 3.2-8.7) and lowest among children 24-59 months (0.5, 95% CI 0.1-1.5). The RSV positive children were more likely to have signs of respiratory distress like wheeze, chest in-drawing, tachypnea, and crepitation compared to RSV-negative based on bivariate comparisons. Other less commonly seen signs of respiratory distress, ie, nasal flaring, grunting, accessory muscle usage were also significantly associated with being RSV positive. Compared to the estimated RSV hospitalization rate based on all medical hospitalizations, the WHO-ARI case definition captured 86% of the total incidence, while case definitions requiring fever like ILI and SARI underestimated the incidence by 50-80%. CONCLUSIONS: Our study suggests that RSV is a substantial cause of hospitalization among children aged <24months especially those aged <6 months. The WHO-ARI case definition appeared to be the most suitable screening definition for RSV surveillance because of its high sensitivity.	eng	PubMed
2021	Kohns Vasconcelos, Malte; Loens, Katherine; Sigfrid, Louise; Iosifidis, Elias; Epalza, Cristina; Donà, Daniele; Matheeussen, Veerle; Papachristou, Savvas; Roilides, Emmanuel; Gijon, Manuel; Rojo, Pablo; Minotti, Chiara; Da Dalt, Liviana; Islam, Samsul; Jarvis, Jessica; Syggelou, Aggeliki; Tsolia, Maria; Nyirenda Nyang'wa, Maggie; Keers, Sophie; Renk, Hanna; Gemmel, Anna-Lena; D'Amore, Carmen; Ciofi Degli Atti, Marta; Rodríguez-Tenreiro Sánchez, Carmen; Martinón-Torres, Federico; Burokienė, Sigita; Goetghebuer, Tessa; Spoulou, Vana; Riordan, Andrew; Calvo, Cristina; Gkentzi, Despoina; Hufnagel, Markus; Openshaw, Peter J.; de Jong, Menno D.; Koopmans, Marion; Goossens, Herman; Ieven, Margareta; Fraaij, Pieter L. A.; Giaquinto, Carlo; Bielicki, Julia A.; Horby, Peter; Sharland, Michael	Aetiology of acute respiratory infection in preschool children requiring hospitalisation in Europe-results from the PED-MERMAIDS multicentre case-control study	BMJ open respiratory research	10.1136/bmjresp-2021-000887	BACKGROUND: Both pathogenic bacteria and viruses are frequently detected in the nasopharynx (NP) of children in the absence of acute respiratory infection (ARI) symptoms. The aim of this study was to estimate the aetiological fractions for ARI hospitalisation in children for respiratory syncytial virus (RSV) and influenza virus and to determine whether detection of specific respiratory pathogens on NP samples was associated with ARI hospitalisation. METHODS: 349 children up to 5 years of age hospitalised for ARI (following a symptom-based case definition) and 306 hospital controls were prospectively enrolled in 16 centres across seven European Union countries between 2016 and 2019. Admission day NP swabs were analysed by multiplex PCR for 25 targets. RESULTS: RSV was the leading single cause of ARI hospitalisations, with an overall population attributable fraction (PAF) of 33.4% and high seasonality as well as preponderance in younger children. Detection of RSV on NP swabs was strongly associated with ARI hospitalisation (OR adjusted for age and season: 20.6, 95% CI: 9.4 to 45.3). Detection of three other viral pathogens showed strong associations with ARI hospitalisation: influenza viruses had an adjusted OR of 6.1 (95% CI: 2.5 to 14.9), parainfluenza viruses (PIVs) an adjusted OR of 4.6 (95% CI: 1.8 to 11.3) and metapneumoviruses an adjusted OR of 4.5 (95% CI: 1.3 to 16.1). Influenza viruses had a PAF of 7.9%, PIVs of 6.5% and metapneumoviruses of 3.0%. In contrast, most other pathogens were found in similar proportions in cases and controls, including Streptococcus pneumoniae, which was weakly associated with case status, and endemic coronaviruses. CONCLUSION: RSV is the predominant cause of ARI hospitalisations in young children in Europe and its detection, as well as detection of influenza virus, PIV or metapneumovirus, on NP swabs can establish aetiology with high probability. PAFs for RSV and influenza virus are highly seasonal and age dependent.	eng	PubMed
2016	Wansaula, Zimy; Olsen, Sonja J.; Casal, Mariana G.; Golenko, Catherine; Erhart, Laura M.; Kammerer, Peter; Whitfield, Natalie; McCotter, Orion Z.	Surveillance for severe acute respiratory infections in Southern Arizona, 2010-2014	Influenza and Other Respiratory Viruses	10.1111/irv.12360	BACKGROUND: The Binational Border Infectious Disease Surveillance program began surveillance for severe acute respiratory infections (SARI) on the US-Mexico border in 2009. Here, we describe patients in Southern Arizona. METHODS: Patients admitted to five acute care hospitals that met the SARI case definition (temperature ≥37·8°C or reported fever or chills with history of cough, sore throat, or shortness of breath in a hospitalized person) were enrolled. Staff completed a standard form and collected a nasopharyngeal swab which was tested for selected respiratory viruses by reverse transcription polymerase chain reaction. RESULTS: From October 2010-September 2014, we enrolled 332 SARI patients. Fifty-two percent were male and 48% were white non-Hispanic. The median age was 63 years (47% ≥65 years and 5·2% <5 years). During hospitalization, 51 of 230 (22%) patients required intubation, 120 of 297 (40%) were admitted to intensive care unit, and 28 of 278 (10%) died. Influenza vaccination was 56%. Of 309 cases tested, 49 (16%) were positive for influenza viruses, 25 (8·1%) for human metapneumovirus, 20 (6·5%) for parainfluenza viruses, 16 (5·2%) for coronavirus, 11 (3·6%) for respiratory syncytial virus, 10 (3·2%) for rhinovirus, 4 (1·3%) for rhinovirus/enterovirus, 3 (1·0%) for enteroviruses, and 3 (1·0%) for adenovirus. Among the 49 influenza-positive specimens, 76% were influenza A (19 H3N2, 17 H1N1pdm09, and 1 not subtyped), and 24% were influenza B. CONCLUSION: Influenza viruses were a frequent cause of SARI in hospitalized patients in Southern Arizona. Monitoring respiratory illness in border populations will help better understand the etiologies. Improving influenza vaccination coverage may help prevent some SARI cases.	eng	PubMed

2014	Radin, Jennifer M.; Hawksworth, Anthony W.; Kammerer, Peter E.; Balansay, Melinda; Raman, Rema; Lindsay, Suzanne P.; Brice, Gary T.	Epidemiology of pathogen-specific respiratory infections among three US populations	PloS One	10.1371/journal.pone.0114871	BACKGROUND: Diagnostic tests for respiratory infections can be costly and time-consuming. Improved characterization of specific respiratory pathogens by identifying frequent signs, symptoms and demographic characteristics, along with improving our understanding of coinfection rates and seasonality, may improve treatment and prevention measures. METHODS: Febrile respiratory illness (FRI) and severe acute respiratory infection (SARI) surveillance was conducted from October 2011 through March 2013 among three US populations: civilians near the US-Mexico border, Department of Defense (DoD) beneficiaries, and military recruits. Clinical and demographic questionnaire data and respiratory swabs were collected from participants, tested by PCR for nine different respiratory pathogens and summarized. Age stratified characteristics of civilians positive for influenza and recruits positive for rhinovirus were compared to other and no/unknown pathogen. Seasonality and coinfection rates were also described. RESULTS: A total of 1444 patients met the FRI or SARI case definition and were enrolled in this study. Influenza signs and symptoms varied across age groups of civilians. Recruits with rhinovirus had higher percentages of pneumonia, cough, shortness of breath, congestion, cough, less fever and longer time to seeking care and were more likely to be male compared to those in the no/unknown pathogen group. Coinfections were found in 6% of all FRI/SARI cases tested and were most frequently seen among children and with rhinovirus infections. Clear seasonal trends were identified for influenza, rhinovirus, and respiratory syncytial virus. CONCLUSIONS: The age-stratified clinical characteristics associated with influenza suggest that age-specific case definitions may improve influenza surveillance and identification. Improving identification of rhinoviruses, the most frequent respiratory infection among recruits, may be useful for separating out contagious individuals, especially when larger outbreaks occur. Overall, describing the epidemiology of pathogen specific respiratory diseases can help improve clinical diagnoses, establish baselines of infection, identify outbreaks, and help prioritize the development of new vaccines and treatments.	eng	PubMed
2016	Cui, Dawei; Feng, Luzhao; Chen, Yu; Lai, Shengjie; Zhang, Zike; Yu, Fei; Zheng, Shufa; Li, Zhongjie; Yu, Hongjie	Clinical and Epidemiologic Characteristics of Hospitalized Patients with Laboratory-Confirmed Respiratory Syncytial Virus Infection in Eastern China between 2009 and 2013: A Retrospective Study	PloS One	10.1371/journal.pone.0165437	Respiratory syncytial virus (RSV) is a leading cause of morbidity and mortality worldwide in children aged <5 years and older adults with acute lower respiratory infections (ALRIs). However, few studies regarding the epidemiology of hospitalizations for RSV infection have been performed previously in China. Here, we aimed to describe the clinical and epidemiologic characteristics of hospitalized patients with laboratory-confirmed RSV infection in eastern China. Active surveillance for hospitalized ALRI patients using a broad case definition based on symptoms was performed from 2009-2013 in 12 sentinel hospitals in eastern China. Clinical and epidemiologic data pertaining to hospitalized patients of all ages with laboratory-confirmed RSV infection by PCR assay were collected and analyzed in this study. From 2009 to 2013, 1046 hospitalized patients with laboratory-confirmed RSV infection were enrolled in this study, and 14.7% of patients had subtype A, 24.2% of patients had subtype B, 23.8% of patients with subtype not performed, and 37.3% of patients had RSV coinfections with other viruses. RSV and influenza coinfections (33.3%) were the most common coinfections noted in this study. Moreover, young children aged <5 years (89.1%, 932/1046), particularly young infants aged <1 year (43.3%, 453/1046), represented the highest proportion of patients with RSV infections. In contrast, older adults aged ≥60 years (1.1%, 12/1046) represented the lowest proportion of patients with RSV infections among enrolled patients. The peak RSV infection period occurred mainly during autumn and winter, and 57% and 66% of patients exhibited symptoms such as fever (body temperature ≥38°C) and cough separately. Additionally, only a small number of patients were treated with broad-spectrum antiviral drugs, and most of patients were treated with antimicrobial drugs that were not appropriate for RSV infection. RSV is a leading viral pathogen and a common cause of viral infection in young children aged <5 years with ALRIs in eastern China. Effective vaccines and antiviral agents targeting RSV are needed to mitigate its large public health impact.	eng	PubMed
2012	Feikin, Daniel R.; Njenga, M. Kariuki; Bigogo, Godfrey; Aura, Barrack; Aol, George; Audi, Allan; Jagero, Geoffrey; Muluare, Peter Ochieng; Gikunju, Stella; Nderitu, Leonard; Balish, Amanda; Winchell, Jonas; Schneider, Eileen; Erdman, Dean; Oberste, M. Steven; Katz, Mark A.; Breiman, Robert F.	Etiology and Incidence of viral and bacterial acute respiratory illness among older children and adults in rural western Kenya, 2007-2010	PloS One	10.1371/journal.pone.0043656	BACKGROUND: Few comprehensive data exist on disease incidence for specific etiologies of acute respiratory illness (ARI) in older children and adults in Africa. METHODOLOGY/PRINCIPAL FINDINGS: From March 1, 2007, to February 28, 2010, among a surveillance population of 21,420 persons >5 years old in rural western Kenya, we collected blood for culture and malaria smears, nasopharyngeal and oropharyngeal swabs for quantitative real-time PCR for ten viruses and three atypical bacteria, and urine for pneumococcal antigen testing on outpatients and inpatients meeting a ARI case definition (cough or difficulty breathing or chest pain and temperature >38.0 °C or oxygen saturation <90% or hospitalization). We also collected swabs from asymptomatic controls, from which we calculated pathogen-attributable fractions, adjusting for age, season, and HIV-status, in logistic regression. We calculated incidence by pathogen, adjusting for health-seeking for ARI and pathogen-attributable fractions. Among 3,406 ARI patients >5 years old (adjusted annual incidence 12.0 per 100 person-years), influenza A virus was the most common virus (22% overall; 11% inpatients, 27% outpatients) and Streptococcus pneumoniae was the most common bacteria (16% overall; 23% inpatients, 14% outpatients), yielding annual incidences of 2.6 and 1.7 episodes per 100 person-years, respectively. Influenza A virus, influenza B virus, respiratory syncytial virus (RSV) and human metapneumovirus were more prevalent in swabs among cases (22%, 6%, 8% and 5%, respectively) than controls. Adenovirus, parainfluenza viruses, rhinovirus/enterovirus, parechovirus, and Mycoplasma pneumoniae were not more prevalent among cases than controls. Pneumococcus and non-typhi Salmonella were more prevalent among HIV-infected adults, but prevalence of viruses was similar among HIV-infected and HIV-negative individuals. ARI incidence was highest during peak malaria season. CONCLUSIONS/SIGNIFICANCE: Vaccination against influenza and pneumococcus (by potential herd immunity from childhood vaccination or of HIV-infected adults) might prevent much of the substantial ARI incidence among persons >5 years old in similar rural African settings.	eng	PubMed
2012	Kammerer, Peter E.; Montiel, Sonia; Kriner, Paula; Bojorquez, Ietza; Bejarano Ramirez, Veronica; Vazquez-Erlbeck, Martha; Azziz-Baumgartner, Eduardo; Blair, Patrick J.; Hawksworth, A. W.; Faix, D. J.; Nava, M. L.; Lopez, L. Wong; Palacios, E.; Flores, R.; Fonseca-Ford, M.; Phippard, A.; Lopez, K.; Johnson, J.; Bustamante Moreno, J. G.; Russell, K. L.; Waterman, S. H.	Influenza-like illness surveillance on the California-Mexico border, 2004-2009	Influenza and Other Respiratory Viruses	10.1111/j.1750-2659.2011.00316.x	BACKGROUND: Since 2004, the Naval Health Research Center, with San Diego and Imperial counties, has collaborated with the US Centers for Disease Control and Prevention to conduct respiratory disease surveillance in the US-Mexico border region. In 2007, the Secretariat of Health, Mexico and the Institute of Public Health of Baja California joined the collaboration. OBJECTIVES: The identification of circulating respiratory pathogens in respiratory specimens from patients with influenza-like illness (ILI). METHODS: Demographic, symptom information and respiratory swabs were collected from enrollees who met the case definition for ILI. Specimens underwent PCR testing and culture in virology and bacteriology. RESULTS: From 2004 through 2009, 1855 persons were sampled. Overall, 36% of the participants had a pathogen identified. The most frequent pathogen was influenza (25%), with those aged 6-15 years the most frequently affected. In April 2009, a young female participant from Imperial County, California, was among the first documented cases of 2009 H1N1. Additional pathogens included influenza B, adenovirus, parainfluenza virus, respiratory syncytial virus, enterovirus, herpes simplex virus, Streptococcus pneumoniae, and Streptococcus pyogenes. CONCLUSIONS: The US-Mexico border is one of the busiest in the world, with a large number of daily crossings. Due to its traffic, this area is an ideal location for surveillance sites. We identified a pathogen in 36% of the specimens tested, with influenza A the most common pathogen. A number of other viral and bacterial respiratory pathogens were identified. An understanding of the incidence of respiratory pathogens in border populations is useful for development of regional vaccination and disease prevention responses.	eng	PubMed
2012	Hombrouck, A.; Sabbe, M.; Van Casteren, V.; Wuillaume, F.; Hue, D.; Reynders, M.; Gérard, C.; Brochier, B.; Van Eldere, J.; Van Ranst, M.; Thomas, I.	Viral aetiology of influenza-like illness in Belgium during the influenza A(H1N1)2009 pandemic	European Journal of Clinical Microbiology & Infectious Diseases: Official Publication of the European Society of Clinical Microbiology	10.1007/s10096-011-1398-4	The purpose of this investigation was to determine the proportion of influenza-like illness (ILI) attributable to specific viruses during the influenza A(H1N1)2009 pandemic and to describe the demographic and clinical characteristics of ILI due to respiratory viruses in Belgium. Nasopharyngeal swabs were collected from ILI patients by general practitioners (GPs) and paediatricians (PediSurv) and analysed for viruses. Of 139 samples collected from children <5 years of age by PediSurv, 86 were positive, including 28 influenza (20%), 27 respiratory syncytial virus (RSV) (19%), 21 rhinovirus (17%), 12 human metapneumovirus (hMPV) (9%) and ten parainfluenza virus (PIV) (7%). Of 810 samples received from GPs, 426 were influenza (53%). Of 312 influenza-negative samples, 41 were rhinovirus (13%), 13 RSV (4%), 11 PIV (4%) and three hMPV (1%). Influenza mostly affected the 6-15 years old age group. Other respiratory viruses were commonly detected in the youngest patients. Similar clinical symptoms were associated with different respiratory viruses. Influenza A(H1N1)2009 was the most detected virus in ILI patients during the 2009-2010 winter, suggesting a good correlation between ILI case definition and influenza diagnosis. However, in children under 5 years of age, other respiratory viruses such as RSV were frequently diagnosed. Furthermore, our findings do not suggest that the early occurrence of the influenza A(H1N1)2009 epidemic impacted the RSV epidemic in Belgium.	eng	PubMed

2021	van Summeren, J. J. G. T.; Rizzo, C.; Hooiveld, M.; Korevaar, J. C.; Hendriksen, J. M. T.; Dückers, M. L. A.; Loconsole, D.; Chironna, M.; Bangert, M.; Demont, C.; Meijer, A.; Caini, S.; Pandolfi, E.; Paget, J.	Evaluation of a standardised protocol to measure the disease burden of respiratory syncytial virus infection in young children in primary care	BMC infectious diseases	10.1186/s12879-021-06397-w	BACKGROUND: A better understanding of the burden of respiratory syncytial virus (RSV) infections in primary care is needed for policymakers to make informed decisions regarding new preventive measures and treatments. The aim of this study was to develop and evaluate a protocol for the standardised measurement of the disease burden of RSV infection in primary care in children aged < 5 years. METHODS: The standardised protocol was evaluated in Italy and the Netherlands during the 2019/20 winter. Children aged < 5 years who consulted their primary care physician, met the WHO acute respiratory infections (ARI) case definition, and had a laboratory confirmed positive test for RSV (RT-PCR) were included. RSV symptoms were collected at the time of swabbing. Health care use, duration of symptoms and socio-economic impact was measured 14 days after swabbing. Health related Quality of life (HRQoL) was measured using the parent-proxy report of the PedsQL™4.0 generic core scales (2-4 years) and PedsQL™4.0 infant scales (0-2 years) 30 days after swabbing. The standardised protocol was evaluated in terms of the feasibility of patient recruitment, data collection procedures and whether parents understood the questions. RESULTS: Children were recruited via a network of paediatricians in Italy and a sentinel influenza surveillance network of general practitioners in the Netherlands. In Italy and the Netherlands, 293 and 152 children were swabbed respectively, 119 and 32 tested RSV positive; for 119 and 12 children the Day-14 questionnaire was completed and for 116 and 11 the Day-30 questionnaire. In Italy, 33% of the children had persistent symptoms after 14 days and in the Netherlands this figure was 67%. Parents had no problems completing questions concerning health care use, duration of symptoms and socio-economic impact, however, they had some difficulties scoring the HRQoL of their young children. CONCLUSION: RSV symptoms are common after 14 days, and therefore, measuring disease burden outcomes like health care use, duration of symptoms, and socio-economic impact is also recommended at Day-30. The standardised protocol is suitable to measure the clinical and socio-economic disease burden of RSV in young children in primary care.	eng	PubMed
2021	Löwensteyn, Yvette N.; Mazur, Natalie I.; Nair, Harish; Willemsen, Joukje E.; van Thiel, Ghislaine; Bont, Louis; RSV GOLD III—ICU Network study group	Describing global pediatric RSV disease at intensive care units in GAVI-eligible countries using molecular point-of-care diagnostics: the RSV GOLD-III study protocol	BMC infectious diseases	10.1186/s12879-021-06544-3	BACKGROUND: Respiratory syncytial virus (RSV) infection is an important cause of hospitalization and death in young children. The majority of deaths (99%) occur in low- and lower-middle-income countries (LMICs). Vaccines against RSV infection are underway. To obtain access to RSV interventions, LMICs depend on support from Gavi, the Vaccine Alliance. To identify future vaccine target populations, information on children with severe RSV infection is required. However, there is a lack of individual patient-level clinical data on instances of life-threatening RSV infection in LMICs. The RSV GOLD III-ICU Network study aims to describe clinical, demographic and socioeconomic characteristics of children with life-threatening RSV infection in Gavi-eligible countries. METHODS: The RSV GOLD-III-ICU Network study is an international, prospective, observational multicenter study and will be conducted in 10 Gavi-eligible countries at pediatric intensive care units and high-dependency units (PICUs/HDUs) during local viral respiratory seasons for 2 years. Children younger than 2 years of age with respiratory symptoms fulfilling the World Health Organization (WHO) "extended severe acute respiratory infection (SARI)" case definition will be tested for RSV using a molecular point-of-care (POC) diagnostic device. Patient characteristics will be collected through a questionnaire. Mortality rates of children admitted to the PICU and/or HDU will be calculated. DISCUSSION: This multicenter descriptive study will provide a better understanding of the characteristics and mortality rates of children younger than 2 years with RSV infection admitted to the PICU/HDU in LMICs. These results will contribute to knowledge on global disease burden and awareness of RSV and will directly guide decision makers in their efforts to implement future RSV prevention strategies. TRIAL REGISTRATION NUMBER: NL9519, May 27, 2021.	eng	PubMed
2019	Alchikh, M.; Conrad, T.; Hoppe, C.; Ma, X.; Broberg, E.; Penttinen, P.; Reiche, J.; Biere, B.; Schweiger, B.; Rath, B.	Are we missing respiratory viral infections in infants and children? Comparison of a hospital-based quality management system with standard of care	Clinical Microbiology and Infection: The Official Publication of the European Society of Clinical Microbiology and Infectious Diseases	10.1016/j.cmi.2018.05.023	OBJECTIVES: Hospital-based surveillance of influenza and acute respiratory infections relies on International Classification of Diseases (ICD) codes and hospital laboratory reports (Standard-of-Care). It is unclear how many cases are missed with either method, i.e. remain undiagnosed/coded as influenza and other respiratory virus infections. Various influenza-like illness (ILI) definitions co-exist with little guidance on how to use them. We compared the diagnostic accuracy of standard surveillance methods with a prospective quality management (QM) programme at a Berlin children's hospital with the Robert Koch Institute. METHODS: Independent from routine care, all patients fulfilling pre-defined ILI-criteria (QM-ILI) participated in the QM programme. A separate QM team conducted standardized clinical assessments and collected nasopharyngeal specimens for blinded real-time quantitative PCR for influenza A/B viruses, respiratory syncytial virus, adenovirus, rhinovirus and human metapneumovirus. RESULTS: Among 6073 individuals with ILI qualifying for the QM programme, only 8.7% (528/6073) would have undergone virus diagnostics during Standard-of-Care. Surveillance based on ICD codes would have missed 61% (359/587) of influenza diagnoses. Of baseline ICD codes, 53.2% (2811/5282) were non-specific, most commonly J06 ('acute upper respiratory infection'). Comparison of stakeholder case definitions revealed that QM-ILI and the WHO ILI case definition showed the highest overall sensitivities (84%-97% and 45%-68%, respectively) and the CDC ILI definition had the highest sensitivity for influenza infections (36%, 95% CI 31.4-40.8 for influenza A and 48%, 95% CI 40.5-54.7 for influenza B). CONCLUSIONS: Disease-burden estimates and surveillance should account for the underreporting of cases in routine care. Future studies should explore the effect of ILI screening and surveillance in various age groups and settings. Diagnostic algorithms should be based on the WHO ILI case definition combined with targeted testing.	eng	PubMed
2022	Reisner, Andrew; Blackwell, Laura S.; Sayeed, Iqbal; Myers, Hannah E.; Wali, Bushra; Heilman, Stacy; Figueroa, Janet; Lu, Austin; Hussaini, Laila; Anderson, Evan J.; Shane, Andi L.; Rostad, Christina A.	Osteopontin as a biomarker for COVID-19 severity and multisystem inflammatory syndrome in children: A pilot study	Experimental Biology and Medicine (Maywood, N.J.)	10.1177/15353702211046835	This study sought to evaluate the candidacy of plasma osteopontin (OPN) as a biomarker of COVID-19 severity and multisystem inflammatory condition in children (MIS-C) in children. A retrospective analysis of 26 children (0-21 years of age) admitted to Children's Healthcare of Atlanta with a diagnosis of COVID-19 between March 17 and May 26, 2020 was undertaken. The patients were classified into three categories based on COVID-19 severity levels: asymptomatic or minimally symptomatic (control population, admitted for other non-COVID-19 conditions), mild/moderate, and severe COVID-19. A fourth category of children met the Centers for Disease Control and Prevention's case definition for MIS-C. Residual blood samples were analyzed for OPN, a marker of inflammation using commercial ELISA kits (R&D), and results were correlated with clinical data. This study demonstrates that OPN levels are significantly elevated in children hospitalized with moderate and severe COVID-19 and MIS-C compared to OPN levels in mild/asymptomatic children. Further, OPN differentiated among clinical levels of severity in COVID-19, while other inflammatory markers including maximum erythrocyte sedimentation rate, C-reactive protein and ferritin, minimum lymphocyte and platelet counts, soluble interleukin-2R, and interleukin-6 did not. We conclude OPN is a potential biomarker of COVID-19 severity and MIS-C in children that may have future clinical utility. The specificity and positive predictive value of this marker for COVID-19 and MIS-C are areas for future larger prospective research studies.	eng	PubMed
2020	Klink, Thomas; Rankin, Danielle A.; Piya, Bhinnata; Spieker, Andrew J.; Faouri, Samir; Shehabi, Asem; Williams, John V.; Khuri-Bulos, Najwa; Halasa, Natasha B.	Evaluating the diagnostic accuracy of the WHO Severe Acute Respiratory Infection (SARI) criteria in Middle Eastern children under two years over three respiratory seasons	PloS One	10.1371/journal.pone.0232188	OBJECTIVE: The World Health Organization created the Severe Acute Respiratory Infection (SARI) criteria in 2011 to monitor influenza (flu)-related hospitalization. Many studies have since used the SARI case definition as inclusion criteria for surveillance studies. We sought to determine the sensitivity, specificity, positive predictive value, and negative predictive value of the SARI criteria for detecting ten different respiratory viruses in a Middle Eastern pediatric cohort. MATERIALS AND METHODS: The data for this study comes from a prospective acute respiratory surveillance study of hospitalized children <2 years in Amman, Jordan from March 16, 2010 to March 31, 2013. Participants were recruited if they had a fever and/or respiratory symptoms. Nasal and throat swabs were obtained and tested by real-time RT-PCR for eleven viruses. Subjects meeting SARI criteria were determined post-hoc. Sensitivity, specificity, positive predictive value, and negative predictive value of the SARI case definition for detecting ten different viruses were calculated and results were stratified by age. RESULTS: Of the 3,175 patients enrolled, 3,164 were eligible for this study, with a median age of 3.5 months, 60.4% male, and 82% virus-positive (44% RSV and 3.8% flu). The sensitivity and specificity of the SARI criteria for detecting virus-positive patients were 44% and 77.9%, respectively. Sensitivity of SARI criteria for any virus was lowest in children <3 months at 22.4%. Removing fever as a criterion improved the sensitivity by 65.3% for detecting RSV in children <3 months; whereas when cough was removed, the sensitivity improved by 45.5% for detecting flu in same age group. CONCLUSIONS: The SARI criteria have poor sensitivity for detecting RSV, flu, and other respiratory viruses-particularly in children <3 months. Researchers and policy makers should use caution if using the criteria to estimate burden of disease in children.	eng	PubMed
2005	Akin, Levent; Surlu, Bige; Bozkaya, Emel; Aslan, Seyhan Selvi; Onal, Atilla; Badur, Selim	Influenza and respiratory syncytial virus morbidity among 0-19 aged group in Yunus Emre Health Center	The Turkish Journal of Pediatrics		The objective of the study was to determine the morbidity of influenza and respiratory syncytial virus (RSV) infection in the 0-19 years of age group with influenza-like illness among the outpatient cases. From 20 January to 31 March 2003 a total of 123 subjects with upper respiratory tract infection attended Yunus Emre Health Center. Ninety-one subjects fit the case definition of influenza-like illness, which consisted of acute fever of more than 38 degrees C, cough, and sore throat. After obtaining their consent, nasal swabs were taken for isolation of influenza and RSV. Of these, 10 were influenza A virus, 6 were influenza B virus and 20 were RSV. All of influenza virus A was typed as subtype H3N2. The rates of influenza virus among 5-9 and 1-4 years of age groups and of RSV among 1-4 years of age group were high. The average number of absentee days of schoolchildren with influenza was 3.33 days and of those with RSV infection was 1.43 days; this rate was calculated as 2.25 days for the influenza-like illness. Continuous surveillance and influenza vaccination for target groups are recommended for beneficial effects of reducing influenza morbidity and mortality in the community.	eng	PubMed

2020	Rahman, H.; Carter, I.; Basile, K.; Donovan, L.; Kumar, S.; Tran, T.; Ko, D.; Alderson, S.; Sivaruban, T.; Eden, J.-S.; Rockett, R.; O'Sullivan, M. V.; Sintchenko, V.; Chen, S. C.-A.; Maddocks, S.; Dwyer, D. E.; Kok, J.	Interpret with caution: An evaluation of the commercial AusDiagnostics versus in-house developed assays for the detection of SARS-CoV-2 virus	Journal of Clinical Virology: The Official Publication of the Pan American Society for Clinical Virology	10.1016/j.jcv.2020.104374	INTRODUCTION: There is limited data on the analytical performance of commercial nucleic acid tests (NATs) for laboratory confirmation of COVID-19 infection. METHODS: Nasopharyngeal, combined nose and throat swabs, nasopharyngeal aspirates and sputum was collected from persons with suspected SARS-CoV-2 infection, serial dilutions of SARS-CoV-2 viral cultures and synthetic positive controls (gBlocks, Integrated DNA Technologies) were tested using i) AusDiagnostics assay (AusDiagnostics Pty Ltd); ii) in-house developed assays targeting the E and RdRp genes; iii) multiplex PCR assay targeting endemic respiratory viruses. Discrepant SARS-CoV-2 results were resolved by testing the N, ORF1b, ORF1ab and M genes. RESULTS: Of 52 clinical samples collected from 50 persons tested, respiratory viruses were detected in 22 samples (42 %), including SARS CoV-2 (n = 5), rhinovirus (n = 7), enterovirus (n = 5), influenza B (n = 4), hMPV (n = 5), influenza A (n = 2), PIV-2 (n = 1), RSV (n = 2), CoV-NL63 (n = 1) and CoV-229E (n = 1). SARS-CoV-2 was detected in four additional samples by the AusDiagnostics assay. Using the in-house assays as the "gold standard", the sensitivity, specificity, positive and negative predictive values of the AusDiagnostics assay was 100 %, 92.16 %, 55.56 % and 100 % respectively. The Ct values of the real-time in-house-developed PCR assay targeting the E gene was significantly lower than the corresponding RdRp gene assay when applied to clinical samples, viral culture and positive controls (mean 21.75 vs 28.1, p = 0.0031). CONCLUSIONS: The AusDiagnostics assay is not specific for the detection SARS-CoV-2. Any positive results should be confirmed using another NAT or sequencing. The case definition used to investigate persons with suspected COVID-19 infection is not specific.	eng	PubMed
2012	Neu, Natalie; Plaskett, Theresa; Hutcheon, Gordon; Murray, Meghan; Southwick, Karen L.; Saiman, Lisa	Epidemiology of human metapneumovirus in a pediatric long-term care facility	Infection Control and Hospital Epidemiology	10.1086/665727	BACKGROUND: Viral respiratory pathogens cause outbreaks in pediatric long-term care facilities (LTCFs), but few studies have used viral diagnostic testing to identify the causative pathogens. We describe the use of such testing during a prolonged period of respiratory illness and elucidate the epidemiology of human metapneumovirus (hMPV) at our LTCF. DESIGN: Retrospective study of influenza-like illness (ILI). SETTING: A 136-bed pediatric LTCF from January 1 through April 30, 2010. METHODS: The ILI case definition included fever, cough, change in oropharyngeal secretions, increase in oxygen requirement, and/or wheezing. RESULTS: During the study period, 69 episodes of ILI occurred in 61 (41%) of 150 residents. A viral pathogen was detected in 27 (39%) of the episodes, including respiratory syncytial virus (RSV) (n = 3), influenza A virus (not typed; n = 2), parainfluenza virus (n =2), adenovirus (n = 1), and hMPV (n = 19). Twenty-seven of the residents with ILI (44%) required transfer to acute care hospitals (mean length of hospitalization, 12 days; range, 3-47 days). Residents with tracheostomies were more likely to have ILI (adjusted odds ratio [OR], 3.99 [95% confidence interval {CI}, 1.87-8.53]; P = .0004). The mortality rate for residents with ILI was 1.6%. Residents with hMPV were younger (P = .03), more likely to be transferred to an acute care facility (OR, 3.73 [95% CI, 1.17-11.95]; P = .02), and less likely to have a tracheostomy (adjusted OR, 0.19 [95% CI, 0.047-0.757]; P = .02). DISCUSSION: Diverse pathogens, most notably hMPV, caused ILI in our pediatric LTCF during a prolonged period of time. Viral testing was helpful in characterizing the epidemiology of ILI in this population.	eng	PubMed
2012	Ahmed, Jamal A.; Katz, Mark A.; Auko, Eric; Njenga, M. Kariuki; Weinberg, Michelle; Kapella, Bryan K.; Burke, Heather; Nyoka, Raymond; Gichangi, Anthony; Waiboci, Lilian W.; Mahamud, Abdirahman; Qassim, Mohamed; Swai, Babu; Wagacha, Burton; Mutonga, David; Nguhi, Margaret; Breiman, Robert F.; Eidex, Rachel B.	Epidemiology of respiratory viral infections in two long-term refugee camps in Kenya, 2007-2010	BMC infectious diseases	10.1186/1471-2334-12-7	BACKGROUND: Refugees are at risk for poor outcomes from acute respiratory infections (ARI) because of overcrowding, suboptimal living conditions, and malnutrition. We implemented surveillance for respiratory viruses in Dadaab and Kakuma refugee camps in Kenya to characterize their role in the epidemiology of ARI among refugees. METHODS: From 1 September 2007 through 31 August 2010, we obtained nasopharyngeal (NP) and oropharyngeal (OP) specimens from patients with influenza-like illness (ILI) or severe acute respiratory infections (SARI) and tested them by RT-PCR for adenovirus (AdV), respiratory syncytial virus (RSV), human metapneumovirus (hMPV), parainfluenza viruses (PIV), and influenza A and B viruses. Definitions for ILI and SARI were adapted from those of the World Health Organization. Proportions of cases associated with viral aetiology were calculated by camp and by clinical case definition. In addition, for children < 5 years only, crude estimates of rates due to SARI per 1000 were obtained. RESULTS: We tested specimens from 1815 ILI and 4449 SARI patients (median age = 1 year). Proportion positive for virus were AdV, 21.7%; RSV, 12.5%; hMPV, 5.7%; PIV, 9.4%; influenza A, 9.7%; and influenza B, 2.6%; 49.8% were positive for at least one virus. The annual rate of SARI hospitalisation for 2007-2010 was 57 per 1000 children per year. Virus-positive hospitalisation rates were 14 for AdV; 9 for RSV; 6 for PIV; 4 for hMPV; 5 for influenza A; and 1 for influenza B. The rate of SARI hospitalisation was highest in children < 1 year old (156 per 1000 child-years). The ratio of rates for children < 1 year and 1 to < 5 years old was 3.7:1 for AdV, 5.5:1 for RSV, 4.4:1 for PIV, 5.1:1 for hMPV, 3.2:1 for influenza A, and 2.2:1 for influenza B. While SARI hospitalisation rates peaked from November to February in Dadaab, no distinct seasonality was observed in Kakuma. CONCLUSIONS: Respiratory viral infections, particularly RSV and AdV, were associated with high rates of illness and make up a substantial portion of respiratory infection in these two refugee settings.	eng	PubMed
2007	Bellei, Nancy; Carraro, Emerson; Perosa, Ana; Granato, Celso	Patterns of influenza infections among different risk groups in Brazil	The Brazilian Journal of Infectious Diseases: An Official Publication of the Brazilian Society of Infectious Diseases	10.1590/s1413-86702007000400005	Influenza virus infections are associated with high morbidity and mortality. Influenza activity varies worldwide, and regional detection is influenced by geographic conditions, demographic and patient-risk factors. We assessed influenza activity and patterns of seasonality during three consecutive years (2001-2003) in three risk groups in São Paulo city. Four-hundred-twelve outpatients with acute respiratory infection were subjected to epidemiological, clinical and laboratory investigations; these included community population (N=140), health-care workers (N=203), and renal-transplanted patients (N=69). Nasal wash samples were tested by direct fluorescent assay for influenza, parainfluenza, adenovirus, and respiratory syncytial virus. Overall Influenza positivity was 21%, and a progressive decline was observed in all groups over time. Influenza A and B co-circulated at the same time in 2001 and 2002, but not in 2003. Low influenza-vaccination rates (19%) were reported by health-care workers. Unexpected low levels of etiological agents were detected in renal-transplanted patients, and infected cases were less symptomatic than immunocompetent patients. Based on this study, we conclude that health-care worker-immunization programs should be implemented and the clinical patterns of infected influenza patients should be used as a guide for better case-definition criteria for adequate influenza surveillance, particularly for renal-transplant patients.	eng	PubMed
2014	Kono, Jacinta; Jonduo, Marinjho H.; Omena, Matthew; Siba, Peter M.; Horwood, Paul F.	Viruses associated with influenza-like-illnesses in Papua New Guinea, 2010	Journal of Medical Virology	10.1002/jmv.23786	Influenza-like-illness can be caused by a wide range of respiratory viruses. The etiology of influenza-like-illness in developing countries such as Papua New Guinea is poorly understood. The etiological agents associated with influenza-like-illness were investigated retrospectively for 300 nasopharyngeal swabs received by the Papua New Guinea National Influenza Centre in 2010. Real-time PCR/RT-PCR methods were used for the detection of 13 respiratory viruses. Patients with influenza-like-illness were identified according to the World Health Organization case definition: sudden onset of fever (>38°C), with cough and/or sore throat, in the absence of other diagnoses. At least one viral respiratory pathogen was detected in 66.3% of the samples tested. Rhinoviruses (17.0%), influenza A (16.7%), and influenza B (12.7%) were the pathogens detected most frequently. Children <5 years of age presented with a significantly higher rate of at least one viral pathogen and a significantly higher rate of co-infections with multiple viruses, when compared to all other patients >5 years of age. Influenza B, adenovirus, and respiratory syncytial virus were all detected at significantly higher rates in children <5 years of age. This study confirmed that multiple respiratory viruses are circulating and contributing to the presentation of influenza-like-illness in Papua New Guinea.	eng	PubMed
2019	La Vincente, S. F.; von Mollendorf, C.; Ulziibayar, M.; Satzke, C.; Dashtseren, L.; Fox, K. K.; Dunne, E. M.; Nguyen, C. D.; de Campo, J.; de Campo, M.; Thomson, H.; Surenkhand, G.; Demberelsuren, S.; Bujinlkham, S.; Do, L. a. H.; Narangerel, D.; Cherian, T.; Mungun, T.; Mulholland, E. K.	Evaluation of a phased pneumococcal conjugate vaccine introduction in Mongolia using enhanced pneumonia surveillance and community carriage surveys: a study protocol for a prospective observational study and lessons learned	BMC public health	10.1186/s12889-019-6639-y	BACKGROUND: Streptococcus pneumoniae causes substantial morbidity and mortality among children. The introduction of pneumococcal conjugate vaccines (PCV) has the potential to dramatically reduce disease burden. As with any vaccine, it is important to evaluate PCV impact, to help guide decision-making and resource-allocation. Measuring PCV impact can be complex, particularly to measure impact on one of the most common and significant diseases caused by the pneumococcus, namely pneumonia. Here we outline the protocol developed to evaluate the impact of 13-valent PCV (PCV13) on childhood pneumonia in Mongolia, and a number of lessons learned in implementing the evaluation that may be helpful to other countries seeking to undertake pneumonia surveillance. METHODS: From 2016 PCV13 was introduced in a phased manner into the routine immunisation programme with some catch-up by the Government of Mongolia. We designed an evaluation to measure vaccine impact in children aged 2-59 months with hospitalised radiological pneumonia as a primary outcome, with secondary objectives to measure impact on clinically-defined pneumonia, nasopharyngeal carriage of S. pneumoniae among pneumonia patients and in the community, and severe respiratory infection associated with RSV and/or influenza. We enhanced an existing hospital-based pneumonia surveillance system by incorporating additional study components (nasopharyngeal swabbing using standard methods, C-reactive protein, risk factor assessment) and strengthening clinical practices, such as radiology as well as monitoring and training. We conducted cross-sectional community carriage surveys to provide data on impact on carriage among healthy children. DISCUSSION: Establishing a robust surveillance system is an important component of monitoring the impact of PCV within a country. The enhanced surveillance system in Mongolia will facilitate assessment of PCV13 impact on pneumonia, with radiological confirmed disease as the primary outcome. Key lessons arising from this evaluation have included the importance of establishing a core group of in-country staff to be responsible for surveillance activities and to work closely with this team; to be aware of external factors that could potentially influence disease burden estimates; to be flexible in data collection processes to respond to changing circumstances and lastly to ensure a consistent application of the pneumonia surveillance case definition throughout the study period.	eng	PubMed

2007	Luna, Luciano Kleber de Souza; Panning, Marcus; Grywna, Klaus; Pfefferle, Susanne; Drosten, Christian	Spectrum of viruses and atypical bacteria in intercontinental air travelers with symptoms of acute respiratory infection	The Journal of Infectious Diseases	10.1086/511432	Respiratory infections after air travel are frequent, but epidemiological data are incomplete. Using sensitive polymerase chain reactions, we studied the spectrum of atypical bacteria and respiratory viruses in travelers fulfilling the case definition of severe acute respiratory syndrome. A pathogen was identified in 67 travelers (43.2%). Influenza and parainfluenza viruses were most prevalent, at 14.2% and 15.5%, respectively. Prevalences of adenoviruses, human metapneumovirus, coronaviruses, and rhinoviruses ranged between 2.6% and 4.8%. Human bocavirus, respiratory syncytial virus, and Legionella, Mycoplasma, and Chlamydophila species were absent or appeared at frequencies of <1%. To our knowledge, these are the first specific baseline data for the mentioned agents in the context of air travel.	eng	PubMed
2014	Ratnamohan, Vigneswary Mala; Taylor, Janette; Zeng, Frank; McPhie, Kenneth; Blyth, Christopher C.; Adamson, Sheena; Kok, Jen; Dwyer, Dominic E.	Pandemic clinical case definitions are non-specific: multiple respiratory viruses circulating in the early phases of the 2009 influenza pandemic in New South Wales, Australia	Virology Journal	10.1186/1743-422X-11-113	BACKGROUND: During the early phases of the 2009 pandemic, subjects with influenza-like illness only had laboratory testing specific for the new A(H1N1)pdm09 virus. FINDINGS: Between 25th May and 7th June 2009, during the pandemic CONTAIN phase, A(H1N1)pdm09 virus was detected using nucleic acid tests in only 56 of 1466 (3.8%) samples meeting the clinical case definition required for A(H1N1)pdm09 testing. Two hundred and fifty-five randomly selected A(H1N1)pdm09 virus-negative samples were tested for other respiratory viruses using a real-time multiplex PCR assay. Of the 255 samples tested, 113 (44.3%) had other respiratory viruses detected: rhinoviruses 63.7%, seasonal influenza A 17.6%, respiratory syncytial virus 7.9%, human metapneumovirus 5.3%, parainfluenzaviruses 4.4%, influenza B virus 4.4%, and enteroviruses 0.8%. Viral co-infections were present in 4.3% of samples. CONCLUSIONS: In the very early stages of a new pandemic, limiting testing to only the novel virus will miss other clinically important co-circulating respiratory pathogens.	eng	PubMed
2006	Fuller, Julie; Hanley, Keith; Schultz, Robert; Lewis, Michael; Freed, Nikki E.; Ellis, Michael; Ngauy, Viseth; Stoebner, Richard; Ryan, Margaret; Russell, Kevin	Surveillance for febrile respiratory infections during Cobra Gold 2003	Military Medicine	10.7205/milmed.171.5.357	The Naval Health Research Center conducted laboratory-based surveillance for febrile respiratory infections at the 2003 Cobra Gold Exercise in Thailand. Seventeen individuals met the case definition for febrile respiratory illness, and diagnostic specimens were obtained from 16. Laboratory testing identified influenza A for 44%; sequence analysis demonstrated that these were Fujian-like influenza strains, which represented the predominant strain found globally in 2003/2004. Other pathogens identified included coronavirus OC43, respiratory syncytial virus, and rhinovirus. Logistical challenges were overcome as laboratory-supported febrile respiratory illness surveillance was conducted during a military training exercise. With heightened concern over the potential for another global influenza pandemic, such surveillance could prove critical for the detection of emerging influenza and respiratory pathogen strains with potential for importation to the United States.	eng	PubMed
2006	Ruscoe, Quentin; Hill, Sarah; Blackmore, Timothy; McLean, Margot	An outbreak of Legionella pneumophila suspected to be associated with spa pools on display at a retail store in New Zealand	The New Zealand Medical Journal		AIM: To investigate and characterise a cluster of six cases of severe pneumonia in the Wellington region notified to Regional Public Health in November 2003. And to describe the public health response to an identified subgroup of Legionella infections. METHODS: The case definition was "a person admitted to Wellington or Hutt Hospital between 29 October 2003 and 9 November 2003 with severe pneumonia". The cluster was initially investigated by interviewing patients to obtain histories of activities and exposures, and by reviewing medical notes. Medical teams sent further clinical specimens for testing (sputum for polymerase chain reaction [PCR], convalescent Legionella serology, and urine for Legionella antigen testing). Further investigation of Legionella pneumophila cases involved obtaining detailed exposure histories, environmental investigations at the suspect source of infection, and taking water and biofilm swabs for Legionella detection and serotyping. RESULTS: Three cases from the cluster were confirmed as, or compatible with, Legionella pneumophila serogroup 2. With the other three cases there was evidence of infection with L. longbeachae (two cases) or respiratory syncytial virus (one case). Exposure histories for the L. pneumophila cases revealed that the three cases had visited a Lower Hutt retail outlet with operating spa pools on display. Legionella pneumophila serogroup 1 was cultured from one of three pools. All pools were positive for Legionella on direct fluorescent antibody testing. CONCLUSIONS: Although unproven, the display spa pools were considered to be the most likely source of Legionella infection in the three cases that had visited the retail outlet. Although Legionella isolated from the pools was a different serogroup from that identified in two cases, the pools were considered to be the most likely source of infection in view of inadequate chlorination of the waters. Public health intervention to address the immediate and longer-term health risks from the pools is described. This is the second outbreak of Legionella pneumophila linked to operating display spa pools in New Zealand and it appears to be the fourth recorded outbreak of Legionnaires' disease associated with operating spa pools on display.	eng	PubMed
