# Supplementary material for: What ‘case definition’ for respiratory syncytial virus infection? Results of a systematic literature review to improve surveillance among the adults
Source: J Public Health (Oxf). 2024 May 5;46(3):326–34. doi: 10.1093/pubmed/fdae066 (PMC11914876; doi:10.1093/pubmed/fdae066)
Supplement: hRSV-Scopus_fdae066 [file hrsv-scopus_fdae066.pdf]

Publication Year	Author	Title	Publication Title	DOI	Abstract Note	Language	Archive
2016	Cui, D.; Feng, L.; Chen, Y.; Lai, S.; Zhang, Z.; Yu, F.; Zheng, S.; Li, Z.; Yu, H.	Clinical and epidemiologic characteristics of hospitalized patients with laboratory-confirmed respiratory syncytial virus infection in eastern China between 2009 and 2013: A retrospective study	PLoS ONE	10.1371/journal.pone.0165437	Respiratory syncytial virus (RSV) is a leading cause of morbidity and mortality worldwide in children aged <5 years and older adults with acute lower respiratory infections (ALRIs). However, few studies regarding the epidemiology of hospitalizations for RSV infection have been performed previously in China. Here, we aimed to describe the clinical and epidemiologic characteristics of hospitalized patients with laboratory-confirmed RSV infection in eastern China. Active surveillance for hospitalized ALRI patients using a broad case definition based on symptoms was performed from 2009-2013 in 12 sentinel hospitals in eastern China. Clinical and epidemiologic data pertaining to hospitalized patients of all ages with laboratory-confirmed RSV infection by PCR assay were collected and analyzed in this study. From 2009 to 2013,1046 hospitalized patients with laboratory-confirmed RSV infection were enrolled in this study, and 14.7% of patients had subtype A, 24.2% of patients had subtype B, 23.8% of patients with subtype not performed, and 37.3% of patients had RSV coinfections with other viruses. RSV and influenza coinfections (33.3%) were the most common coinfections noted in this study. Moreover, young children aged <5 years (89.1%, 932/1046), particularly young infants aged <1 year (43.3%, 453/1046), represented the highest proportion of patients with RSV infections. In contrast, older adults aged ≥60 years (1.1%, 12/1046) represented the lowest proportion of patients with RSV infections among enrolled patients. The peak RSV infection period occurred mainly during autumn and winter, and 57% and 66% of patients exhibited symptoms such as fever (body temperature ≥38°C) and cough separately. Additionally, only a small number of patients were treated with broad-spectrum antiviral drugs, and most of patients were treated with antimicrobial drugs that were not appropriate for RSV infection. RSV is a leading viral pathogen and a common cause of viral infection in young children aged <5 years with ALRIs in eastern China. Effective vaccines and antiviral agents targeting RSV are needed to mitigate its large public health impact. © 2016 Cui et al.This is an open access article distributed under the terms of the Creative Commons Attribution License, which permits unrestricted use, distribution, and reproduction in any medium, provided the original author and source are credited.	English	Scopus
2022	Li, Y.; Wang, X.; Blau, D.M.; Caballero, M.T.; Feikin, D.R.; Gill, C.J.; Madhi, S.A.; Omer, S.B.; Simões, E.A.F.; Campbell, H.; Pariente, A.B.; Bardach, D.; Bassat, Q.; Casalegno, J.-S.; Chakhunashvili, G.; Crawford, N.; Danilenko, D.; Do, L.A.H.; Echavarria, M.; Gentile, A.; Gordon, A.; Heikkinen, T.; Huang, Q.S.; Jullien, S.; Krishnan, A.; Lopez, E.L.; Markić, J.; Mira-Iglesias, A.; Moore, H.C.; Moyes, J.; Mwananyanda, L.; Nokes, D.J.; Noordeen, F.; Obodai, E.; Palani, N.; Romero, C.; Salimi, V.; Satav, A.; Seo, E.; Shchomak, Z.; Singleton, R.; Stolyarov, K.; Stoszek, S.K.; von Gottberg, A.; Wurzel, D.; Yoshida, L.-M.; Yung, C.F.; Zar, H.J.; Abram, M.; Aerssens, J.; Alafaci, A.; Balmaseda, A.; Bandeira, T.; Barr, I.; Batinović, E.; Beutels, P.; Bhiman, J.; Blyth, C.C.; Bont, L.; Bressler, S.S.; Cohen, C.; Cohen, R.; Costa, A.-M.; Crow, R.; Daley, A.; Dang, D.-A.; Demont, C.; Desnoyers, C.; Díez-Domingo, J.; Divarathna, M.; du Plessis, M.; Edgoose, M.; Ferolla, F.M.; Fischer, T.K.; Gebremedhin, A.; Giaquinto, C.; Gillet, Y.; Hernandez, R.; Horvat, C.;	Global, regional, and national disease burden estimates of acute lower respiratory infections due to respiratory syncytial virus in children younger than 5 years in 2019: a systematic analysis	The Lancet	10.1016/S0140-6736(22)00478-0	Background: Respiratory syncytial virus (RSV) is the most common cause of acute lower respiratory infection in young children. We previously estimated that in 2015, 33·1 million episodes of RSV-associated acute lower respiratory infection occurred in children aged 0–60 months, resulting in a total of 118 200 deaths worldwide. Since then, several community surveillance studies have been done to obtain a more precise estimation of RSV associated community deaths. We aimed to update RSV-associated acute lower respiratory infection morbidity and mortality at global, regional, and national levels in children aged 0–60 months for 2019, with focus on overall mortality and narrower infant age groups that are targeted by RSV prophylactics in development. Methods: In this systematic analysis, we expanded our global RSV disease burden dataset by obtaining new data from an updated search for papers published between Jan 1, 2017, and Dec 31, 2020, from MEDLINE, Embase, Global Health, CINAHL, Web of Science, LILACS, OpenGrey, CNKI, Wanfang, and ChongqingVIP. We also included unpublished data from RSV GEN collaborators. Eligible studies reported data for children aged 0–60 months with RSV as primary infection with acute lower respiratory infection in community settings, or acute lower respiratory infection necessitating hospital admission; reported data for at least 12 consecutive months, except for in-hospital case fatality ratio (CFR) or for where RSV seasonality is well-defined; and reported incidence rate, hospital admission rate, RSV positive proportion in acute lower respiratory infection hospital admission, or in-hospital CFR. Studies were excluded if case definition was not clearly defined or not consistently applied, RSV infection was not laboratory confirmed or based on serology alone, or if the report included fewer than 50 cases of acute lower respiratory infection. We applied a generalised linear mixed-effects model (GLMM) to estimate RSV-associated acute lower respiratory infection incidence, hospital admission, and in-hospital mortality both globally and regionally (by country development status and by World Bank Income Classification) in 2019. We estimated country-level RSV-associated acute lower respiratory infection incidence through a risk-factor based model. We developed new models (through GLMM) that incorporated the latest RSV community mortality data for estimating overall RSV mortality. This review was registered in PROSPERO (CRD42021252400). Findings: In addition to 317 studies included in our previous review, we identified and included 113 new eligible studies and unpublished data from 51 studies, for a total of 481 studies. We estimated that globally in 2019, there were 33·0 million RSV-associated acute lower respiratory infection episodes (uncertainty range [UR] 25·4–44·6 million), 3·6 million RSV-associated acute lower respiratory infection hospital admissions (2·9–4·6 million), 26 300 RSV-associated acute lower respiratory infection in-hospital deaths (15 100–49 100), and 101 400 RSV-attributable overall deaths (84 500–125 200) in children aged 0–60 months. In infants aged 0–6 months, we estimated that there were 6·6 million RSV-associated acute lower respiratory infection episodes (4·6–9·7 million), 1·4 million RSV-associated acute lower respiratory infection hospital admissions (1·0–2·0 million), 13 300 RSV-associated acute lower respiratory infection in-hospital deaths (6800–28 100), and 45 700 RSV-attributable overall deaths (38 400–55 900). 2·0% of deaths in children aged 0–60 months (UR 1·6–2·4) and 3·6% of deaths in children aged 28 days to 6 months (3·0–4·4) were attributable to RSV. More than 95% of RSV-associated acute lower respiratory infection episodes and more than 97% of RSV-attributable deaths across all age bands were in low-income and middle-income countries (LMICs). Interpretation: RSV contributes substantially to morbidity and mortality burden globally in children aged 0–60 months, especially during the first 6 months of life and in LMICs. We highlight the striking overall mortality burden of RSV disease worldwide, with one in every 50 deaths in children aged 0–60 months and one in every 28 deaths in children aged 28 days to 6 months attributable to RSV. For every RSV-associated acute lower respiratory infection in-hospital death, we estimate approximately three more deaths attributable to RSV in the community. RSV passive immunisation programmes targeting protection during the first 6 months of life could have a substantial effect on reducing RSV disease burden, although more data are needed to understand the implications of the potential age-shifts in peak RSV burden to older age when these are implemented. Funding: EU Innovative Medicines Initiative Respiratory Syncytial Virus Consortium in Europe (RESCEU). © 2022 The Author(s). Published by Elsevier Ltd. This is an Open Access article under the CC BY 4.0 license	English	Scopus
2012	Hombrouck, A.; Sabbe, M.; Van Casteren, V.; Wuillaume, F.; Hue, D.; Reynders, M.; Gérard, C.; Brochier, B.; Van Eldere, J.; Van Ranst, M.; Thomas, I.	Viral aetiology of influenza-like illness in Belgium during the influenza A(H1N1)2009 pandemic	European Journal of Clinical Microbiology and Infectious Diseases	10.1007/s10096-011-1398-4	The purpose of this investigation was to determine the proportion of influenza-like illness (ILI) attributable to specific viruses during the influenza A(H1N1)2009 pandemic and to describe the demographic and clinical characteristics of ILI due to respiratory viruses in Belgium. Nasopharyngeal swabs were collected from ILI patients by general practitioners (GPs) and paediatricians (PediSurv) and analysed for viruses. Of 139 samples collected from children <5 years of age by PediSurv, 86 were positive, including 28 influenza (20%), 27 respiratory syncytial virus (RSV) (19%), 21 rhinovirus (17%), 12 human metapneumovirus (hMPV) (9%) and ten parainfluenza virus (PIV) (7%). Of 810 samples received from GPs, 426 were influenza (53%). Of 312 influenza-negative samples, 41 were rhinovirus (13%), 13 RSV (4%), 11 PIV (4%) and three hMPV (1%). Influenza mostly affected the 6-15 years old age group. Other respiratory viruses were commonly detected in the youngest patients. Similar clinical symptoms were associated with different respiratory viruses. Influenza A(H1N1)2009 was the most detected virus in ILI patients during the 2009-2010 winter, suggesting a good correlation between ILI case definition and influenza diagnosis. However, in children under 5 years of age, other respiratory viruses such as RSV were frequently diagnosed. Furthermore, our findings do not suggest that the early occurrence of the influenza A(H1N1)2009 epidemic impacted the RSV epidemic in Belgium. © Springer-Verlag 2011.	English	Scopus
2021	Löwensteyn, Y.N.; Mazur, N.I.; Nair, H.; Willemsen, J.E.; van Thiel, G.; Bont, L.; Garba, M.A.; Giwa, F.J.; Rasooly, M.H.; Shirpoor, A.; Azizyar, M.; Makalo, L.; Nyan, O.; Mohamed, A.; Osman, K.; Chapagain, R.H.; Bista, K.P.; Sharma, A.K.; Shrestha, P.; Goka, B.; Osman, K.; Obodai, E.; Mandi, H.; Esong, L.E.; Eposse, C.E.; Muando, V.; Mussá, T.; Said, Y.H.; Shoo, A.A.; Dor, V.J.; Gautier, J.; Abicher, L.	Describing global pediatric RSV disease at intensive care units in GAVI-eligible countries using molecular point-of-care diagnostics: the RSV GOLD-III study protocol	BMC Infectious Diseases	10.1186/s12879-021-06544-3	Background: Respiratory syncytial virus (RSV) infection is an important cause of hospitalization and death in young children. The majority of deaths (99%) occur in low- and lower-middle-income countries (LMICs). Vaccines against RSV infection are underway. To obtain access to RSV interventions, LMICs depend on support from Gavi, the Vaccine Alliance. To identify future vaccine target populations, information on children with severe RSV infection is required. However, there is a lack of individual patient-level clinical data on instances of life-threatening RSV infection in LMICs. The RSV GOLD III—ICU Network study aims to describe clinical, demographic and socioeconomic characteristics of children with life-threatening RSV infection in Gavi-eligible countries. Methods: The RSV GOLD-III—ICU Network study is an international, prospective, observational multicenter study and will be conducted in 10 Gavi-eligible countries at pediatric intensive care units and high-dependency units (PICUs/HDUs) during local viral respiratory seasons for 2 years. Children younger than 2 years of age with respiratory symptoms fulfilling the World Health Organization (WHO) “extended severe acute respiratory infection (SARI)” case definition will be tested for RSV using a molecular point-of-care (POC) diagnostic device. Patient characteristics will be collected through a questionnaire. Mortality rates of children admitted to the PICU and/or HDU will be calculated. Discussion: This multicenter descriptive study will provide a better understanding of the characteristics and mortality rates of children younger than 2 years with RSV infection admitted to the PICU/HDU in LMICs. These results will contribute to knowledge on global disease burden and awareness of RSV and will directly guide decision makers in their efforts to implement future RSV prevention strategies. Trial registration number: NL9519, May 27, 2021 © 2021, The Author(s).	English	Scopus

2021	Komoyo, G.F.; Yambiyo, B.M.; Manirakiza, A.; Gody, J.C.; Muller, C.P.; Hübschen, J.M.; Nakoune, E.; Snoeck, C.J.	Epidemiology and genetic characterization of respiratory syncytial virus in children with acute respiratory infections: Findings from the influenza sentinel surveillance network in Central African Republic, 2015 to 2018	Health Science Reports	10.1002/hsr2.298	Background and aims: Respiratory syncytial virus (RSV) is one of the main viral pathogens causing acute respiratory infections in children under 5 years of age but has seldom been studied in Central African Republic (CAF). Taking advantage of the national influenza surveillance network in CAF, this study aimed at providing the first insights into RSV prevalence and seasonality over 4 years of surveillance and the clinical manifestations of RSV in this population in CAF. Methods: A total of 3903 children under 5 years matching the influenza-like illness (ILI, 68.5%) or severe acute respiratory infection (SARI, 31.5%) case definitions were recruited from January 2015 to December 2018. The presence of RSV viral RNA in nasopharyngeal samples was assessed by RT-PCR, followed by RSV-A and RSV-B typing and Sanger sequencing on a subset of samples. Phylogenetic analyses were carried on partial G protein sequences. Associations between RSV and demographic or clinical manifestations were investigated by statistical analyses. Results: RSV prevalence was significantly higher in infants <6 months (13.4%), in hospitalized children (13.3% vs 5.5%) and in male patients (9.5% vs 6.4%). An overall prevalence of RSV of 8.0% in the period of 2015 to 2018 was shown, with significant annual (6.4%-10.6%) and seasonal (12.7% in rainy season vs 3.0% in dry season) fluctuations. While RSV seasons in 2015, 2016, and 2018 were relatively similar, 2017 showed deviations from the overall patterns with significantly higher RSV circulation and an outbreak peak 3 to 5 months earlier. Concomitant circulation of RSV-A and RSV-B with an alternating predominance of RSV-A and RSV-B strains and temporal RSV-A genotype replacement from NA1 to ON1 was observed. Conclusion: This study represents the first in-depth epidemiological analysis of RSV in CAF and provides first insights into RSV genetic diversity and seasonality in the country. © 2021 The Authors. Health Science Reports published by Wiley Periodicals LLC.	English	Scopus
2021	Rolsma, S.L.; Rankin, D.A.; Haddadin, Z.; Hamdan, L.; Rahman, H.K.; Faouri, S.; Shehabi, A.; Williams, J.V.; Khuri-Bulos, N.; Halasa, N.B.	Assessing the epidemiology and seasonality of influenza among children under two hospitalized in Amman, Jordan, 2010-2013	Influenza and other Respiratory Viruses	10.1111/irv.12813	Background: The disease burden of influenza-associated hospitalizations among children in Jordan is not well established. We aimed to characterize hospitalizations attributed to influenza in a pediatric population. Methods: We conducted a cross-sectional study from our viral surveillance cohort in children under 2 years hospitalized with acute respiratory symptoms and/or fever from March 2010 to March 2013. We collected demographic and clinical characteristics, and calculated the frequency of children who met the severe acute respiratory illness (SARI) criteria. Nasal specimens were tested using real-time reverse transcriptase polymerase chain reaction to detect influenza A, B, or C. Further subtyping for influenza A-positive isolates was conducted. Results: Of the 3168 children enrolled in our study, 119 (4%) were influenza-positive. Influenza types and subtypes varied by season but were predominantly detected between December and February. Codetection of multiple respiratory pathogens was identified in 58% of children with the majority occurring among those <6 months. Bronchopneumonia and rule-out sepsis were the most common admission diagnoses, with influenza A accounting for over 2/3 of children with a rule-out sepsis admission status. One-third of children under 6 months compared to 3/4 of children 6-23 months met the SARI criteria. Conclusions: Influenza was an important cause of acute respiratory illness in children under 2 years. Children <6 months had the highest burden of influenza-associated hospitalizations and were less likely to meet the SARI global surveillance case definition. Additional surveillance is needed in the Middle East to determine the true influenza burden on a global scale. © 2020 The Authors. Influenza and Other Respiratory Viruses Published by John Wiley & Sons Ltd.	English	Scopus
2020	Rahman, H.; Carter, I.; Basile, K.; Donovan, L.; Kumar, S.; Tran, T.; Ko, D.; Alderson, S.; Sivaruban, T.; Eden, J.-S.; Rockett, R.; O'Sullivan, M.V.; Sintchenko, V.; Chen, S.C.-A.; Maddocks, S.; Dwyer, D.E.; Kok, J.	Interpret with caution: An evaluation of the commercial AusDiagnostics versus in-house developed assays for the detection of SARS-CoV-2 virus	Journal of Clinical Virology	10.1016/j.jcv.2020.104374	Introduction: There is limited data on the analytical performance of commercial nucleic acid tests (NATs) for laboratory confirmation of COVID-19 infection. Methods: Nasopharyngeal, combined nose and throat swabs, nasopharyngeal aspirates and sputum was collected from persons with suspected SARS-CoV-2 infection, serial dilutions of SARS-CoV-2 viral cultures and synthetic positive controls (gBlocks, Integrated DNA Technologies) were tested using i) AusDiagnostics assay (AusDiagnostics Pty Ltd); ii) in-house developed assays targeting the E and RdRp genes; iii) multiplex PCR assay targeting endemic respiratory viruses. Discrepant SARS-CoV-2 results were resolved by testing the N, ORF1b, ORF1ab and M genes. Results: Of 52 clinical samples collected from 50 persons tested, respiratory viruses were detected in 22 samples (42 %), including SARS CoV-2 (n = 5), rhinovirus (n = 7), enterovirus (n = 5), influenza B (n = 4), hMPV (n = 5), influenza A (n = 2), PIV-2 (n = 1), RSV (n = 2), CoV-NL63 (n = 1) and CoV-229E (n = 1). SARS-CoV-2 was detected in four additional samples by the AusDiagnostics assay. Using the in-house assays as the "gold standard", the sensitivity, specificity, positive and negative predictive values of the AusDiagnostics assay was 100 %, 92.16 %, 55.56 % and 100 % respectively. The Ct values of the real-time in-house-developed PCR assay targeting the E gene was significantly lower than the corresponding RdRp gene assay when applied to clinical samples, viral culture and positive controls (mean 21.75 vs 28.1, p = 0.0031). Conclusions: The AusDiagnostics assay is not specific for the detection SARS-CoV-2. Any positive results should be confirmed using another NAT or sequencing. The case definition used to investigate persons with suspected COVID-19 infection is not specific. © 2020 Elsevier B.V.	English	Scopus
2020	Aamir, U.B.; Salman, M.; Nisar, N.; Badar, N.; Alam, M.M.; Ansari, J.; Zaidi, S.S.Z.	Molecular characterization of circulating respiratory syncytial virus genotypes in Pakistani children, 2010–2013	Journal of Infection and Public Health	10.1016/j.jiph.2019.05.014	Background: Data on the viral etiology of acute lower respiratory infections are scarce in Pakistan. Human respiratory syncytial virus (RSV) is an important cause of morbidity in children but no effective vaccine or antiviral therapy is currently available. As vaccines are expected to become available in the future, it is important to understand the epidemiology of locally prevalent RSV subtypes. This study aimed to define the molecular epidemiology of RSV (A and B) genotypes in Pakistani children under 5 years. Methods: World Health Organization case definitions for influenza-like illness (ILI) and severe acute respiratory illness (SARI) were used for case selection. Children under 5 years who presented with ILI or SARI at tertiary care hospitals from all provinces/regions, including the eight influenza sentinel sites, during October–April each year between 2010 and 2013 were enrolled. Demographic and clinical data of the children were recorded and nasopharyngeal/throat swabs taken for analysis. All samples were tested for RSV A and B using real-time polymerase chain reaction for non-influenza respiratory viruses. Specific oligonucleotide primers for RSV A and B were used for subtyping and sequencing of the G protein, followed by phylogenetic analysis. Results: A total of 1941 samples were included. RSV was detected in 472 (24%) children, with RSV A detected in 367 (78%) and RSV B in 105 (22%). The G protein of all RSV A strains clustered in the NA1/GA2 genotype while RSV B strains carried the signature 60 nucleotide duplication and were assigned to three BA genotypes: BA-9, BA-10 and the new BA-13 genotype. Conclusions: This study highlights the importance of RSV as a viral etiologic agent of acute respiratory infections in children in Pakistan, and the diversity of RSV viruses. Continued molecular surveillance for early detection of prevalent and newly emerging genotypes is needed to understand the epidemiology of RSV infections in Pakistan. © 2020 The Authors	English	Scopus
2021	van Summeren, J.J.G.T.; Rizzo, C.; Hooiveld, M.; Korevaar, J.C.; Hendriksen, J.M.T.; Dückers, M.L.A.; Loconsole, D.; Chironna, M.; Bangert, M.; Demont, C.; Meijer, A.; Caini, S.; Pandolfi, E.; Paget, J.	Evaluation of a standardised protocol to measure the disease burden of respiratory syncytial virus infection in young children in primary care	BMC Infectious Diseases	10.1186/s12879-021-06397-w	Background: A better understanding of the burden of respiratory syncytial virus (RSV) infections in primary care is needed for policymakers to make informed decisions regarding new preventive measures and treatments. The aim of this study was to develop and evaluate a protocol for the standardised measurement of the disease burden of RSV infection in primary care in children aged < 5 years. Methods: The standardised protocol was evaluated in Italy and the Netherlands during the 2019/20 winter. Children aged < 5 years who consulted their primary care physician, met the WHO acute respiratory infections (ARI) case definition, and had a laboratory confirmed positive test for RSV (RT-PCR) were included. RSV symptoms were collected at the time of swabbing. Health care use, duration of symptoms and socio-economic impact was measured 14 days after swabbing. Health related Quality of life (HRQoL) was measured using the parent-proxy report of the PedsQL™4.0 generic core scales (2–4 years) and PedsQL™4.0 infant scales (0–2 years) 30 days after swabbing. The standardised protocol was evaluated in terms of the feasibility of patient recruitment, data collection procedures and whether parents understood the questions. Results: Children were recruited via a network of paediatricians in Italy and a sentinel influenza surveillance network of general practitioners in the Netherlands. In Italy and the Netherlands, 293 and 152 children were swabbed respectively, 119 and 32 tested RSV positive; for 119 and 12 children the Day-14 questionnaire was completed and for 116 and 11 the Day-30 questionnaire. In Italy, 33% of the children had persistent symptoms after 14 days and in the Netherlands this figure was 67%. Parents had no problems completing questions concerning health care use, duration of symptoms and socio-economic impact, however, they had some difficulties scoring the HRQoL of their young children. Conclusion: RSV symptoms are common after 14 days, and therefore, measuring disease burden outcomes like health care use, duration of symptoms, and socio-economic impact is also recommended at Day-30. The standardised protocol is suitable to measure the clinical and socio-economic disease burden of RSV in young children in primary care. © 2021, The Author(s).	English	Scopus

2017	Horton, K.C.; Dueger, E.L.; Kandeel, A.; Abdallat, M.; El-Kholy, A.; Al-Awaidy, S.; Kohlani, A.H.; Amer, H.; ElKhal, A.L.; Said, M.; House, B.; Pimentel, G.; Talaat, M.	Viral etiology, seasonality and severity of hospitalized patients with severe acute respiratory infections in the Eastern Mediterranean Region, 2007-2014	PLoS ONE	10.1371/journal.pone.0180954	Introduction Little is known about the role of viral respiratory pathogens in the etiology, seasonality or severity of severe acute respiratory infections (SARI) in the Eastern Mediterranean Region. Methods Sentinel surveillance for SARI was conducted from December 2007 through February 2014 at 20 hospitals in Egypt, Jordan, Oman, Qatar and Yemen. Nasopharyngeal and oropharyngeal swabs were collected from hospitalized patients meeting SARI case definitions and were analyzed for infection with influenza, respiratory syncytial virus (RSV), adenovirus (AdV), human metapneumovirus (hMPV) and human parainfluenza virus types 13 (hPIV1-3). We analyzed surveillance data to calculate positivity rates for viral respiratory pathogens, describe the seasonality of those pathogens and determine which pathogens were responsible for more severe outcomes requiring ventilation and/or intensive care and/or resulting in death. Results At least one viral respiratory pathogen was detected in 8,753/28,508 (30.7%) samples tested for at least one pathogen and 3,497/9,315 (37.5%) of samples tested for all pathogens-influenza in 3,345/28,438 (11.8%), RSV in 3,942/24,503 (16.1%), AdV in 923/9,402 (9.8%), hMPV in 617/9,384 (6.6%), hPIV1 in 159/9,402 (1.7%), hPIV2 in 85/9,402 (0.9%) and hPIV3 in 365/9,402 (3.9%). Multiple pathogens were identified in 501/9,316 (5.4%) participants tested for all pathogens. Monthly variation, indicating seasonal differences in levels of infection, was observed for all pathogens. Participants with hMPV infections and participants less than five years of age were significantly less likely than participants not infected with hMPV and those older than five years of age, respectively, to experience a severe outcome, while participants with a pre-existing chronic disease were at increased risk of a severe outcome, compared to those with no reported pre-existing chronic disease. Conclusions Viral respiratory pathogens are common among SARI patients in the Eastern Mediterranean Region. Ongoing surveillance is important to monitor changes in the etiology, seasonality and severity of pathogens of interest. This is an open access article distributed under the terms of the Creative Commons Attribution License, which permits unrestricted use, distribution, and reproduction in any medium, provided the original author and source are credited.	English	Scopus
2015	Fulton, T.R.; Narayanan, D.; Bonhoeffer, J.; Ortiz, J.R.; Lambach, P.; Omer, S.B.	A systematic review of adverse events following immunization during pregnancy and the newborn period	Vaccine	10.1016/j.vaccine.2015.08.043	In 2013, the WHO Strategic Advisory Group of Experts on Immunization (SAGE) requested WHO to develop a process and a plan to move the maternal immunization agenda forward in support of an increased alignment of data safety evidence, public health needs, and regulatory processes. A key challenge identified was the continued need for harmonization of maternal adverse event following immunization (AEFI) research and surveillance efforts within developing and developed country contexts. We conducted a systematic review as a preliminary step in the development of standardized AEFI definitions for use in maternal and neonatal clinical trials, post-licensure surveillance, and other vaccine studies. We documented the current extent and nature of variability in AEFI definitions and adverse event reporting among 74 maternal immunization studies, which reported a total of 240 different types of adverse events. Forty-nine studies provided explicit AEFI case definitions describing 35 separate types of AEFIs. We identified variability in how AEFIs were determined to be present, in how AEFI definitions were applied, and in the ways that AEFIs were reported. Definitions for key maternal/neonatal AEFIs differed on four discrete attributes: overall level of detail, physiological and temporal boundaries and cut-offs, severity strata, and standards used. Our findings suggest that investigators may proactively address these inconsistencies through comprehensive and consistent reporting of AEFI definitions and outcomes in future publications. In addition, efforts to develop standardized AEFI definitions should generate definitions of sufficient detail and consistency of language to avoid the ambiguities we identified in reviewed articles, while remaining practically applicable given the constraints of low-resource contexts such as limited diagnostic capacity and high patient throughput. © 2015 The Authors.	English	Scopus
2022	Bimouhen, A.; Regragui, Z.; El Falaki, F.; Ihazmade, H.; Benkerroum, S.; Cherkaoui, I.; Rguig, A.; Ezzine, H.; Benamar, T.; Triki, S.; Bakri, Y.; Oumzil, H.	Viral aetiology of influenza-like illnesses and severe acute respiratory illnesses in Morocco, September 2014 to December 2016	Journal of Global Health	10.7189/JOGH.12.04062	Background There is a scarcity of information on the viral aetiology of influenza-like illness (ILI) and severe acute respiratory infection (SARI) among patients in Morocco. Methods From September 2014 to December 2016, we prospectively enrolled inpatients and outpatients from all age groups meeting the World Health Organization (WHO) case definition for ILI and SARI from 59 sentinel sites. The specimens were tested using real-time monoplex reverse-transcription polymerase chain reaction method for detecting 16 relevant respiratory viruses. Results At least one respiratory virus was detected in 1423 (70.8%) of 2009 specimens. Influenza viruses were the most common, detected in 612 (30.4%) of processed samples, followed by respiratory syncytial virus (RSV) in 359 (17.9%), human rhinovirus (HRV) in 263 (13.1%), adenovirus (HAdV) in 124 (6.2%), parainfluenza viruses (HPIV) in 107 (5.3%), coronaviruses (HCoV) in 94 (4.7%), human bocavirus (HBoV) in 92 (4.6%), and human metapneumovirus (HMPV) in 74 (3.7%). From 770 samples from children under 5 years old, RSV (288, 36.6%), influenza viruses (106, 13.8%), HRV (96, 12.5%) and HAdV (91, 11.8%) were most prevalent. Among 955 samples from adults, Influenza viruses (506, 53.0%), and HRV (167, 17.5%) were most often detected. Co-infections were found in 268 (18.8%) of 1423 positive specimens, and most (60.4%) were in children under 5 years of age. While influenza viruses, RSV, and HMPV had a defined period of circulation, the other viruses did not display clear seasonal patterns. Conclusions We found that RSV was predominant among SARI cases in Morocco, particularly in children under 5 years of age. Our results are in line with reported data from other parts of the world, stating that RSV is the leading cause of lower respiratory tract infections in infants and young children. © 2022 The Author(s)	English	Scopus
2019	Alchikh, M.; Conrad, T.; Hoppe, C.; Ma, X.; Broberg, E.; Penttinen, P.; Reiche, J.; Biere, B.; Schweiger, B.; Rath, B.	Are we missing respiratory viral infections in infants and children? Comparison of a hospital-based quality management system with standard of care	Clinical Microbiology and Infection	10.1016/j.cmi.2018.05.023	Objectives: Hospital-based surveillance of influenza and acute respiratory infections relies on International Classification of Diseases (ICD) codes and hospital laboratory reports (Standard-of-Care). It is unclear how many cases are missed with either method, i.e. remain undiagnosed/coded as influenza and other respiratory virus infections. Various influenza-like illness (ILI) definitions co-exist with little guidance on how to use them. We compared the diagnostic accuracy of standard surveillance methods with a prospective quality management (QM) programme at a Berlin children's hospital with the Robert Koch Institute. Methods: Independent from routine care, all patients fulfilling pre-defined ILI-criteria (QM-ILI) participated in the QM programme. A separate QM team conducted standardized clinical assessments and collected nasopharyngeal specimens for blinded real-time quantitative PCR for influenza A/B viruses, respiratory syncytial virus, adenovirus, rhinovirus and human metapneumovirus. Results: Among 6073 individuals with ILI qualifying for the QM programme, only 8.7% (528/6073) would have undergone virus diagnostics during Standard-of-Care. Surveillance based on ICD codes would have missed 61% (359/587) of influenza diagnoses. Of baseline ICD codes, 53.2% (2811/5282) were non-specific, most commonly J06 ('acute upper respiratory infection'). Comparison of stakeholder case definitions revealed that QM-ILI and the WHO ILI case definition showed the highest overall sensitivities (84%–97% and 45%–68%, respectively) and the CDC ILI definition had the highest sensitivity for influenza infections (36%, 95% CI 31.4–40.8 for influenza A and 48%, 95% CI 40.5–54.7 for influenza B). Conclusions: Disease-burden estimates and surveillance should account for the underreporting of cases in routine care. Future studies should explore the effect of ILI screening and surveillance in various age groups and settings. Diagnostic algorithms should be based on the WHO ILI case definition combined with targeted testing. © 2018 European Society of Clinical Microbiology and Infectious Diseases	English	Scopus
2022	Korsten, K.; Adriaenssens, N.; Coenen, S.; Butler, C.C.; Verheij, T.J.M.; Bont, L.J.; Wildenbeest, J.G.	World Health Organization Influenza-Like Illness Underestimates the Burden of Respiratory Syncytial Virus Infection in Community-Dwelling Older Adults	Journal of Infectious Diseases	10.1093/infdis/jiab452	Background. Respiratory syncytial virus (RSV) surveillance is heavily dependent on the influenza-like illness (ILI) case definition from the World Health Organization (WHO). Because ILI includes fever in its syndromic case definition, its ability to accurately identify acute respiratory tract infections (ARTI) caused by RSV in older adults is uncertain. Methods. The accuracy of the WHO ILI and a modified ILI (requiring only self-reported fever) case definitions in identifying patients with PCR-confirmed RSV-ARTI was evaluated in community-dwelling older adults (≥60 years) from the prospective European RESCEU cohort study. Results. Among 1040 participants, 750 ARTI episodes were analyzed including 36 confirmed RSV-ARTI. Due to a general lack of fever, sensitivity for RSV-ARTI was 33% for modified ILI and 11% for ILI. The area under the curve for both ILI definitions was 0.52 indicating poor discrimination for RSV. RSV-ARTI could not be distinguished from all other ARTI based on clinical symptoms. Conclusions. The use of ILI underestimated the occurrence of RSV-ARTI in community-dwelling older adults up to 9-fold (11% sensitivity). Because worldwide RSV surveillance depends largely on ILI, there is an urgent need for a better approach to measure the occurrence of RSV disease and the impact of future RSV vaccine introduction. Clinical Trials Registration. NCT03621930. © The Author(s) 2021. Published by Oxford University Press for the Infectious Diseases Society of America.	English	Scopus
2021	Zhang, Y.; Song, J.; Xu, W.	Attention should be paid to the detection and surveillance of human respiratory syncytial virus	National Medical Journal of China	10.3760/cma.j.cn112137-20210621-01400	Human respiratory syncytial virus (HRSV) is the main pathogen of severe lower respiratory tract infection in infants and young children. It seriously endangers children's health. In recent years, great breakthroughs have been made in the research and development of HRSV vaccines and antibody-based biological products. The research and development and use strategies are inseparable from the monitoring of HRSV prevalence and virus variation characteristics. The World Health Organization (WHO) pays great attentions to the surveillance of HRSV epidemiology and virus variation characteristics, but China lacks national level and multi-center HRSV surveillance data, the surveillance case definitions used by various laboratories are inconsistent, and the detection and surveillance methods of HRSV are not unified. Results from different laboratories are difficult to be compared and analyzed. Therefore, it is urgent to establish a nation-wide HRSV surveillance network in China, and to persistently monitor the epidemic characteristics and virus variation characteristics of HRSV by using standardized HRSV detection methods and surveillance guideline, so as to provide basic scientific data for the research and development, use and evaluation of monoclonal antibodies and vaccines. Copyright © 2021 by the Chinese Medical Association.	Chinese	Scopus

2019	Hatem, A.; Mohamed, S.; Abu Elhassan, U.E.; Ismael, E.A.M.; Rizk, M.S.; El-Kholy, A.; El-Harras, M.	Clinical characteristics and outcomes of patients with severe acute respiratory infections (SARI): Results from the Egyptian surveillance study 2010-2014	Multidisciplinary Respiratory Medicine	10.1186/s40248-019-0174-7	Background: Respiratory viral and atypical bacterial infections data in Egyptian patients are sparse. This study describes the clinical features and outcomes of patients with severe acute respiratory infections (SARI) in hospitalized patients in Egypt. Methods: SARI surveillance was implemented at Cairo University Hospital (CUH) during the period 2010-2014. All hospitalized patients meeting the WHO case definition for SARI were enrolled. Nasopharyngeal/oropharyngeal (NP/OP) swabs were collected and samples were tested using RT-PCR for influenza A, B, respiratory syncytial virus (RSV), human metapneumovirus (hMPV), parainfluenza virus (PIV 1,2,3,4), adenovirus, bocavirus, coronavirus, enterovirus, rhinovirus, and atypical bacteria. Data were analyzed to calculate positivity rates for viral pathogens and determine which pathogens related to severe outcomes or resulted in death. Results: Overall, 1,075/3,207 (33.5%) cases had a viral etiology, with a mean age of 5.74 (±13.87) years. The highest rates were reported for RSV (485 cases, 45.2%), PIV (125, 11.6%), and adenovirus (105, 9.8%). Children had a higher viral rate (981, 91.2%) compared to 94 (8.8%) cases in adults. Patients with identified viruses had significantly lower rates for ICU admission, hospital stay, mechanical ventilation, and overall mortality than those without identified viruses. No infections were independently associated with severe outcomes. Conclusions: Viral pathogens were encountered in one-third of hospitalized adult and pediatric Egyptian patients with SARI, while atypical bacteria had a minor role. Highest rates of viral infections were reported for RSV, PIV, and adenovirus. Viral infections had neither negative impacts on clinical features nor outcomes of patients with SARI in our locality. © 2019 The Author(s).	English	Scopus
2019	an der Heiden, M.; Buchholz, U.; Buda, S.	Estimation of influenza- and respiratory syncytial virus-attributable medically attended acute respiratory infections in Germany, 2010/11-2017/18	Influenza and other Respiratory Viruses	10.1111/irv.12666	Background: The burden of influenza in primary care is difficult to assess, since most patients with symptoms of a respiratory infection are not tested. The case definition of "medically attended acute respiratory infection" (MAARI) in the German physician sentinel is sensitive; however, it requires modelling techniques to derive estimates of disease attributable to influenza and respiratory syncytial virus (RSV). Objectives: The objective of this paper was to review and extend our previously published model in order to estimate the burden of RSV and the differential burden of the two influenza B lineages (Victoria, Yamagata) as well as both influenza A subtypes on primary care visits. Methods: Data on MAARI and virological results of respiratory samples (virological sentinel) were available from 2010/11 until 2017/18. We updated the previously published generalized additive regression model to include RSV. Results: We found that the proportion of MAARI due to RSV is substantial only in the 0-1- and 2-4-year-old age groups (0-1 years old: median 7.5%, range 4.0%-14.8%; 2-4 years old: median 6.5%, range 4.0%-10.3%); in the 0-1 years old age group, RSV leads in almost all seasons to a higher burden than any influenza type or subtype, but this is reversed in the age group 2-4 years old. Conclusions: We succeeded in rearranging our previously published model on MAARI to incorporate RSV as well as the two influenza B lineages (Victoria, Yamagata) in the time period 2010 to 2018. © 2019 The Authors. Influenza and Other Respiratory Viruses Published by John Wiley & Sons Ltd.	English	Scopus
2021	Chaw, P.S.; Hua, L.; Cunningham, S.; Campbell, H.; Mikolajczyk, R.; Nair, H.	Respiratory syncytial virus-associated acute lower respiratory infections in children with bronchopulmonary dysplasia: Systematic review and meta-analysis	Journal of Infectious Diseases	10.1093/INFDIS/JIZ492	Background. Respiratory syncytial virus (RSV) is among the most important causes of acute lower respiratory tract infection (ALRI) in young children. We assessed the severity of RSV-ALRI in children less than 5 years old with bronchopulmonary dysplasia (BPD). Methods. We searched for studies using EMBASE, Global Health, and MEDLINE. We assessed hospitalization risk, intensive care unit (ICU) admission, need for oxygen supplementation and mechanical ventilation, and in-hospital case fatality (hCFR) among children with BPD compared with those without (non-BPD). We compared the (1) length of hospital stay (LOS) and (2) duration of oxygen supplementation and mechanical ventilation between the groups. Results. Twenty-nine studies fulfilled our inclusion criteria. The case definition for BPD varied substantially in the included studies. Risks were higher among children with BPD compared with non-BPD: RSV hospitalization (odds ratio [OR], 2.6; 95% confidence interval [CI], 1.7-4.2; P < .001), ICU admission (OR, 2.9; 95% CI, 2.3-3.5; P < .001), need for oxygen supplementation (OR, 4.2; 95% CI, .5-33.7; P = .175) and mechanical ventilation (OR, 8.2; 95% CI, 7.6-8.9; P < .001), and hCFR (OR, 12.8; 95% CI, 9.4-17.3; P < .001). Median LOS (range) was 7.2 days (4-23) (BPD) compared with 2.5 days (1-30) (non-BPD). Median duration of oxygen supplementation (range) was 5.5 days (0-21) (BPD) compared with 2.0 days (0-26) (non-BPD). The duration of mechanical ventilation was more often longer (>6 days) in those with BPD compared with non-BPD (OR, 11.9; 95% CI, 1.4-100; P = .02). Conclusions. The risk of severe RSV disease is considerably higher among children with BPD. There is an urgent need to establish standardized BPD case definitions, review the RSV prophylaxis guidelines, and encourage more specific studies on RSV infection in BPD patients, including vaccine development and RSV-specific treatment. © 2020 Oxford University Press. All rights reserved.	English	Scopus
2020	Broor, S.; Campbell, H.; Hirve, S.; Hague, S.; Jackson, S.; Moen, A.; Nair, H.; Palekar, R.; Rajatonirina, S.; Smith, P.G.; Venter, M.; Wairagkar, N.; Zambon, M.; Ziegler, T.; Zhang, W.	Leveraging the Global Influenza Surveillance and Response System for global respiratory syncytial virus surveillance—opportunities and challenges	Influenza and other Respiratory Viruses	10.1111/irv.12672	Background: Respiratory syncytial virus (RSV)-associated acute lower respiratory infection is a common cause for hospitalization and hospital deaths in young children globally. There is urgent need to generate evidence to inform immunization policies when RSV vaccines become available. The WHO piloted a RSV surveillance strategy that leverages the existing capacities of the Global Influenza Surveillance and Response System (GISRS) to better understand RSV seasonality, high-risk groups, validate case definitions, and develop laboratory and surveillance standards for RSV. Methods: The RSV sentinel surveillance strategy was piloted in 14 countries. Patients across all age groups presenting to sentinel hospitals and clinics were screened all year-round using extended severe acute respiratory infection (SARI) and acute respiratory infection (ARI) case definitions for hospital and primary care settings, respectively. Respiratory specimens were tested for RSV at the National Influenza Centre (NIC) using standardized molecular diagnostics that had been validated by an External Quality Assurance program. The WHO FluMart data platform was adapted to receive case-based RSV data and visualize interactive visualization outputs. Results: Laboratory standards for detecting RSV by RT-PCR were developed. A review assessed the feasibility and the low incremental costs for RSV surveillance. Several challenges were addressed related to case definitions, sampling strategies, the need to focus surveillance on young children, and the data required for burden estimation. Conclusions: There was no evidence of any significant adverse impact on the functioning of GISRS which is primarily intended for virologic and epidemiological surveillance of influenza. © 2019 The World Health Organization. Influenza and Other Respiratory Viruses published by John Wiley & Sons Ltd.	English	Scopus
2020	Bourgeois, M.; Ausselet, N.; Gerard, V.; De Canniere, L.; Scius, N.; Michaux, I.; Huang, T.-D.; Bogaerts, P.; Vandamme, C.; Bihin, B.; Delaere, B.	Severe influenza/respiratory syncytial virus infections and hospital antimicrobial stewardship opportunities: Impact of a 4-year surveillance including molecular diagnosis	Infection Control and Hospital Epidemiology	10.1017/ice.2020.260	Objective: To assess the prevalence of influenza and respiratory syncytial virus (RSV) in adults hospitalized for a respiratory infection in the winter months and to evaluate the impact of a viral diagnosis on empirical antimicrobial management (antibiotics and antivirals).Design: Observational cohort study.Setting: Acute-care university hospital.Patients: The study included 963 adult patients hospitalized over a 4-year surveillance period.Methods: Annual surveillance timelines were defined according to epidemiological criteria related to the circulation of RSV and influenza viruses in the general population. Patients were screened following a severe acute respiratory infection (SARI) case definition at the emergency department and were enrolled for molecular assay targeting influenza/RSV viruses after oral informed consent. Epidemiological and clinical data were recorded prospectively, microbiological investigations, antimicrobial management, and outcome data were reviewed retrospectively.Results: An influenza or RSV virus was documented in 316 of 963 patients (33%). Optimization of antimicrobial management (AM) was achieved in 162 of 265 patients (61%) with a positive viral diagnosis and no bacterial infection at admission (AM treatment not initiated, n = 111; discontinued, n = 51). In contrast, only 128 of 462 patients (28%) with negative microbiological investigations did not have AM treatment initiated (n = 116) or had such treatment discontinued (n = 12). Early, targeted antiviral treatment was prescribed in 235 of 253 patients (93%) confirmed with influenza. Epidemiological, clinical, and outcome data were similar in both groups.Conclusion: Epidemiological surveillance associated with influenza/RSV molecular diagnosis in adults hospitalized for severe winter respiratory infections dramatically enhanced antimicrobial management. © 2020 Cambridge University Press. All rights reserved.	English	Scopus
2020	Abu Elhassan, U.E.; Mohamed, S.A.A.; Rizk, M.S.; Sherif, M.; El-Harras, M.	Outcomes of patients with severe acute respiratory infections (SARI) admitted to the intensive care unit: Results from the Egyptian surveillance study 2010-2014	Multidisciplinary Respiratory Medicine	10.4081/mrm.2020.465	Background: Few data exist about respiratory viral infections in Egyptian patients. Hereby we describe the outcomes of hospitalized Egyptian patients with severe acute respiratory infections (SARI) admitted to the ICU. Methods: A prospective study in which all hospitalized patients meeting the WHO case definition for SARI and admitted to the ICU, during the period 2010-2014, were enrolled. Samples were tested using RT-PCR for influenza A, B, respiratory syncytial virus (RSV), human metapneumovirus (hMPV), parainfluenza virus, adenovirus, bocavirus, enterovirus, and rhinovirus. Data were analyzed to study the clinical features of SARI-ICU patients and which pathogens are related to severe outcomes. Associated comorbidities were evaluated using Charlson Age-Comorbidity Index (CACI). Results: Out of 1,075 patients with SARI, 219 (20.3%) were admitted to the ICU. The highest rates were reported for RSV (37%). SARI-ICU patients had higher rates of hospital stay, pneumonia, respiratory failure, ARDS, and mortality. Multivariate logistic regression analysis identified associated respiratory disorders (p=0.001), radiological abnormalities (p=0.023), and longer hospital stay (p=0.005) as risk factors for severe outcomes. Conclusions: This surveillance study showed that 20% of hospitalized Egyptian patients with viral SARI needed ICU admission. SARI-ICU patients had higher rates of hospital stay, pneumonia, respiratory failure, ARDS, and mortality. Higher comorbidity index scores, radiological abnormalities, and longer hospital stay are risk factors for severe outcomes in SARI-ICU patients in our locality. © 2020 BioMed Central Ltd.. All rights reserved.	English	Scopus

2016	Modjarrad, K.; Giersing, B.; Kaslow, D.C.; Smith, P.G.; Moorthy, V.S.	WHO consultation on Respiratory Syncytial Virus Vaccine Development Report from a World Health Organization Meeting held on 23-24 March 2015	Vaccine	10.1016/j.vaccine.2015.05.093	Respiratory syncytial virus (RSV) is a globally prevalent cause of lower respiratory infection in neonates and infants. Despite its disease burden, a safe and effective RSV vaccine has remained elusive. In recent years, improved understanding of RSV biology and innovations in immunogen design has resulted in the advancement of multiple vaccine candidates into the clinical development pipeline. Given the growng tested, and the likelihood that an RSV vaccine willing number of vaccines in clinical trials, the rapid pace at which they are bei reach the commercial market in the next 5-10 years, consensus and guidance on clinical development pathways and licensure routes are needed now, before large-scale efficacy trials commence. In pursuit of this aim, the World Health Organization convened the first RSV vaccine consultation in 15 years on the 23rd and 24th of March, 2015 in Geneva, Switzerland. The meeting's primary objective was to provide guidance on clinical endpoints and development pathways for vaccine trials with a focus on considerations of low- and middle-income countries. Meeting participants reached consensus on candidate case definitions for RSV disease, considerations for clinical efficacy endpoints, and the clinical development pathway for active and passive immunization trials in maternal and pediatric populations. The strategic focus of this meeting was on the development of high quality, safe and efficacious RSV preventive interventions for global use and included: (1) maternal/passive immunization to prevent RSV disease in infants less than 6 months; (2) pediatric immunization to prevent RSV disease in infants and young children once protection afforded by maternal immunization wanes. © 2015 World Health Organization; licensee Elsevier.	English	Scopus
2022	Razanajatovo, N.H.; Andrianirina, Z.Z.; Andriatahina, T.; Guillebaud, J.; Harimanana, A.; Ratsima, E.H.; Rakotoariniaina, H.; Orelle, A.; Ratovoson, R.; Irinantenaina, J.; Rakotonanahary, D.A.; Ramparany, L.; Randrianirina, F.; Heraud, J.-M.; Richard, V.	Assessment of surveillance predictors for suspected respiratory syncytial virus, influenza and Streptococcus pneumoniae infections in children aged <5 years in Madagascar	IJID Regions	10.1016/j.ijregi.2021.12.003	Background: The lack of rapid, sensitive and affordable diagnostic tests that can distinguish a wide variety of respiratory pathogens at the point of care is an obstacle to the rapid implementation of control measures following events and epidemics. In addition, the absence of a standardized case definition to differentiate putative aetiologies is a challenge to assessing the burden of disease. This study aimed to identify the clinical spectrum of respiratory pathogens commonly associated with respiratory tract infections in the context of disease surveillance. Methods: Data obtained from prospective hospital-based severe acute respiratory infection surveillance among children aged <5 years from November 2010 to July 2013 were used in this study. Results: Intercostal recession and dyspnoea were predictive of respiratory syncytial virus (RSV) infection, whereas headache and chills were more often observed during influenza A infection. Male patients were at a higher risk for RSV infection than female patients. Productive cough, chills, sweating and weight loss were significantly associated with Streptococcus pneumoniae infection. The presence of fever did not necessarily indicate RSV infection. Conclusions: Combined with other examinations, this study shows the value of including the syndromic approach in the panel of diagnostic criteria for rapid identification of the risk of infectious diseases in areas where laboratory diagnostics are challenging. Given the current situation with coronavirus disease 2019, this approach may help decision makers to implement appropriate control measures. © 2021 The Authors	English	Scopus
2022	Koul, P.A.; Saha, S.; Kaul, K.A.; Mir, H.; Potdar, V.; Chadha, M.; Iuliano, D.; Lafond, K.E.; Lal, R.B.; Krishnan, A.	Respiratory syncytial virus among children hospitalized with severe acute respiratory infection in Kashmir, a temperate region in northern India	Journal of Global Health	10.7189/JOGH.12.04050	Background Severe acute respiratory infections (SARI) are a leading cause of hospitalizations in children, especially due to viral pathogens. We studied the prevalence of respiratory viruses among children aged <5 years hospitalized with severe acute respiratory infections (SARI) in Kashmir, India. Methods We conducted a prospective observational study in two tertiary care hospitals from October 2013 to September 2014, systematically enrolling two children aged <5 years with SARI per day. We defined SARI as history of fever or measured fever (≥38°C) and cough with onset in the last 7 days requiring hospitalization for children aged 3-59 months and as physician-diagnosed acute lower respiratory infection for children aged <3 months. Trained study staff screened children within 24 hours of hospitalization for SARI and collected clinical data and nasopharyngeal swabs from enrolled participants. We tested for respiratory syncytial virus (RSV) A and B, influenza viruses, rhinoviruses (HRV)/enteroviruses, adenovirus (AdV), bocavirus (BoV), human metapneumovirus (hMPV) A and B, coronaviruses (OC43, NL65, C229E), and parainfluenza viruses (PIV) 1, 2, 3 and 4 using standardized duplex real-time polymerase chain reaction. Results Among 4548 respiratory illness admissions screened from October 2013 to September 2014, 1026 met the SARI case definition, and 412 were enrolled (ages = 5 days to 58 months; median = 12 months). Among enrolees, 256 (62%) were positive for any virus; RSV was the most commonly detected (n = 118, 29%) followed by HRV/enteroviruses (n = 88, 21%), PIVs (n = 31, 8%), influenza viruses (n = 18, 4%), BoV (n = 15, 4%), coronaviruses (n = 16, 4%), AdV (n = 14, 3%), and hMPV (n = 9, 2%). Fifty-four children had evidence of virus co-detection. Influenza-associated SARI was more common among children aged 1-5 years (14/18, 78%) while most RSV detections occurred in children <12 months (83/118, 70%). Of the RSV viruses typed (n = 116), the majority were type B (94, 80%). Phylogenetic analysis of G gene of RSV showed circulation of the BA9 genotype with 60bp nucleotide duplication. Conclusions Respiratory viruses, especially RSV, contributed to a substantial proportion of SARI hospitalizations among children <5 years in north India. These data can help guide clinicians on appropriate treatment and prevention strategies. © 2022. The Author(s)	English	Scopus
2016	Campe, H.; Heinzinger, S.; Hartberger, C.; Sing, A.	Clinical symptoms cannot predict influenza infection during the 2013 influenza season in Bavaria, Germany	Epidemiology and Infection	10.1017/S0950268815002228	For influenza surveillance and diagnosis typical clinical symptoms are traditionally used to discriminate influenza virus infections from infections by other pathogens. During the 2013 influenza season we performed a multiplex assay for 16 different viruses in 665 swabs from patients with acute respiratory infections (ARIs) to display the variety of different pathogens causing ARI and to test the diagnostic value of both the commonly used case definitions [ARI, and influenza like illness (ILI)] as well as the clinical judgement of physicians, respectively, to achieve a laboratory-confirmed influenza diagnosis. Fourteen different viruses were identified as causing ARI/ILI. Influenza diagnosis based on clinical signs overestimated the number of laboratory-confirmed influenza cases and misclassified cases. Furthermore, ILI case definition and physicians agreed in only 287/651 (44%) cases with laboratory confirmation. Influenza case management has to be supported by laboratory confirmation to allow evidence-based decisions. Epidemiological syndromic surveillance data should be supported by laboratory confirmation for reasonable interpretation. Copyright © Cambridge University Press 2015.	English	Scopus
2006	Fuller, J.; Hanley, K.; Schultz, R.; Lewis, M.; Freed, N.E.; Ellis, M.; Ngauy, V.; Stoebner, R.; Ryan, M.; Russell, K.	Surveillance for febrile respiratory infections during Cobra Gold 2003	Military Medicine	10.7205/MILMED.171.5.357	The Naval Health Research Center conducted laboratory-based surveillance for febrile respiratory infections at the 2003 Cobra Gold Exercise in Thailand. Seventeen individuals met the case definition for febrile respiratory illness, and diagnostic specimens were obtained from 16. Laboratory testing identified influenza A for 44%; sequence analysis demonstrated that these were Fujian-like influenza strains, which represented the predominant strain found globally in 2003/2004. Other pathogens identified included coronavirus OC43, respiratory syncytial virus, and rhinovirus. Logistical challenges were overcome as laboratory-supported febrile respiratory illness surveillance was conducted during a military training exercise. With heightened concern over the potential for another global influenza pandemic, such surveillance could prove critical for the detection of emerging influenza and respiratory pathogen strains with potential for importation to the United States.	English	Scopus
2020	Lalani, K.; Yildirim, I.; Phadke, V.K.; Bednarczyk, R.A.; Omer, S.B.	Assessment and Validation of Syndromic Case Definitions for Respiratory Syncytial Virus Infections in Young Infants: A Latent Class Analysis	Pediatric Infectious Disease Journal	10.1097/INF.0000000000002468	Background: Respiratory syncytial virus (RSV) is a major cause of pediatric morbidity and mortality worldwide. Standardized case definitions that are applicable to variety of populations are critical for robust surveillance systems to guide decision-making regarding RSV control strategies including vaccine evaluation. Limited data exist on performance of RSV syndromic case definitions among young infants or in high-resource settings. Objective: The purpose of this study was to evaluate existing and potential syndromic case definitions for RSV among young infants in an urban, high-income setting using latent class analyses (LCA). Methods: We used data collected on infants <6 months of age tested for RSV as part of routine clinical care at Children's Healthcare of Atlanta between January 2010 and December 2015. We computed the sensitivity, specificity, positive and negative predictive values of clinical features, existing syndromic case definitions used by the World Health Organization (WHO) and alternative definitions we constructed using LCA to detect RSV infection. Results: Among 565 infants tested for RSV, 161 (28.5%) had laboratory-confirmed RSV infection. Among all case definitions evaluated, the definition developed through LCA (cough plus shortness of breath plus coryza plus wheeze plus poor feeding plus chest in-drawing) was the most specific (95.8%, 95% CI 93.8-97.8) and had the highest positive predictive value (51.4%, 95% CI, 34.9-68.0). WHO-acute respiratory infection (cough or sore throat or shortness of breath or coryza, plus a clinician's judgment that illness is due to infection) was the most sensitive (98.1%, 95% CI, 96.1-100.0; negative predictive value 96.3%, 95% CI 92.2-100.0). Conclusions: The WHO acute respiratory infection definition could be useful for initial screening for RSV among infants <6 months, whereas our alternative syndromic case definition may serve as the strongest confirmatory case definition in the same population. Appropriate case definitions will vary depending on the content and setting in which they are utilized. © 2020 Lippincott Williams and Wilkins. All rights reserved.	English	Scopus

2021	Jiang, W.; Wu, M.; Chen, S.; Li, A.; Wang, K.; Wang, Y.; Chen, Z.; Hao, C.; Shao, X.; Xu, J.	Virus Coinfection is a Predictor of Radiologically Confirmed Pneumonia in Children with Bordetella pertussis Infection	Infectious Diseases and Therapy	10.1007/s40121-020-00376-5	Introduction: This study aimed to prospectively investigate the burden of pertussis in southeast Chinese children hospitalized with lower respiratory tract infection (LRTI) during a pertussis outbreak and to compare the outcomes of Bordetella pertussis infection with or without virus coinfections. Methods: Children < 24 months of age hospitalized with LRTI were prospectively enrolled from January 2017 to December 2019. Demographic and clinical information were recorded, and respiratory tract samples were tested for the presence of B. pertussis and ten common viruses by polymerase chain reaction (PCR). Results: Bordetella pertussis PCR was positive in 6.1% (202/4287) of the patients. Only 146 (72.3%) B. pertussis infections met the Centers for Disease Control and Prevention case definition for pertussis. Among the 202 subjects with B. pertussis infections, 81 (40.1%) were coinfected with at least 1 respiratory virus, with human rhinovirus being the most commonly detected virus (25.7%). No differences in clinical severity were observed between children with single B. pertussis infection and those with virus coinfection [odds ratio (OR) 0.75; 95% confidence interval (CI) 0.39–1.44]. However, children with virus coinfection were significantly more likely to present with radiologically confirmed pneumonia than those with a single B. pertussis infection (OR 2.62; CI 1.39–4.91). Conclusions: Bordetella pertussis infection contributed to a high proportion of LRTI hospitalizations among southeast Chinese children. There were no significant differences in clinical severity between children with virus coinfection and single B. pertussis infection, although children coinfected with virus coinfection presented with pneumonia more frequently than those with single B. pertussis infection. © 2020, The Author(s).	English	Scopus
2014	Ratnamohan, V.M.; Taylor, J.; Zeng, F.; McPhie, K.; Blyth, C.C.; Adamson, S.; Kok, J.; Dwyer, D.E.	Pandemic clinical case definitions are non-specific: Multiple respiratory viruses circulating in the early phases of the 2009 influenza pandemic in New South Wales, Australia	Virology Journal	10.1186/1743-422X-11-113	Background: During the early phases of the 2009 pandemic, subjects with influenza-like illness only had laboratory testing specific for the new A(H1N1)pdm09 virus. Findings. Between 25th May and 7th June 2009, during the pandemic CONTAIN phase, A(H1N1)pdm09 virus was detected using nucleic acid tests in only 56 of 1466 (3.8%) samples meeting the clinical case definition required for A(H1N1)pdm09 testing. Two hundred and fifty-five randomly selected A(H1N1)pdm09 virus-negative samples were tested for other respiratory viruses using a real-time multiplex PCR assay. Of the 255 samples tested, 113 (44.3%) had other respiratory viruses detected: rhinoviruses 63.7%, seasonal influenza A 17.6%, respiratory syncytial virus 7.9%, human metapneumovirus 5.3%, parainfluenzaviruses 4.4%, influenza B virus 4.4%, and enteroviruses 0.8%. Viral co-infections were present in 4.3% of samples. Conclusions: In the very early stages of a new pandemic, limiting testing to only the novel virus will miss other clinically important co-circulating respiratory pathogens. © 2014 Ratnamohan et al.; licensee BioMed Central Ltd.	English	Scopus
2014	Kono, J.; Jonduo, M.H.; Omena, M.; Siba, P.M.; Horwood, P.F.	Viruses associated with influenza-like-illnesses in Papua New Guinea, 2010	Journal of Medical Virology	10.1002/jmv.23786	Influenza-like-illness can be caused by a wide range of respiratory viruses. The etiology of influenza-like-illness in developing countries such as Papua New Guinea is poorly understood. The etiological agents associated with influenza-like-illness were investigated retrospectively for 300 nasopharyngeal swabs received by the Papua New Guinea National Influenza Centre in 2010. Real-time PCR/RT-PCR methods were used for the detection of 13 respiratory viruses. Patients with influenza-like-illness were identified according to the World Health Organization case definition: sudden onset of fever (>38°C), with cough and/or sore throat, in the absence of other diagnoses. At least one viral respiratory pathogen was detected in 66.3% of the samples tested. Rhinoviruses (17.0%), influenza A (16.7%), and influenza B (12.7%) were the pathogens detected most frequently. Children <5 years of age presented with a significantly higher rate of at least one viral pathogen and a significantly higher rate of co-infections with multiple viruses, when compared to all other patients >5 years of age. Influenza B, adenovirus, and respiratory syncytial virus were all detected at significantly higher rates in children <5 years of age. This study confirmed that multiple respiratory viruses are circulating and contributing to the presentation of influenza-like-illness in Papua New Guinea. © 2013 Wiley Periodicals, Inc.	English	Scopus
2016	Soofie, N.; Nunes, M.C.; Kgagudi, P.; Van Niekerk, N.; Makgobo, T.; Agosti, Y.; Hwinya, C.; Pathirana, J.; Madhi, S.A.	The burden of pertussis hospitalization in HIV-exposed and HIV-unexposed south african infants	Clinical Infectious Diseases	10.1093/cid/ciw545	Background. There are limited data on pertussis in African children, including among human immunodeficiency virus (HIV)-exposed infants. We conducted population-based hospital surveillance to determine the incidence and clinical presentation of Bordetella pertussis-associated hospitalization in perinatal HIV-exposed and-unexposed infants. Methods. Children <12 months of age hospitalized with any sign or symptom of respiratory illness (including suspected sepsis or apnea in neonates) were enrolled from 1 January 2015 to 31 December 2015. Detailed clinical and demographic information was recorded and respiratory samples were tested by polymerase chain reaction (PCR). Results. The overall B. pertussis PCR positivity was 2.3% (42/1839), of which 86% (n = 36) occurred in infants <3 months of age. Bordetella pertussis was detected in 2.1% (n = 26/1257) of HIV-unexposed and 2.7% (n = 16/599) of HIV-exposed infants. The incidence (per 1000) of B. pertussis-associated hospitalization was 2.9 (95% confidence interval [CI], 1.8-4.5) and 1.9 (95% CI, 1.3-2.6) in HIV-exposed and HIV-unexposed infants, respectively (P = .09). The overall in-hospital case fatality ratio among the cases was 4.8% (2/42), both deaths of which occurred in HIV-exposed infants <3 months of age. Among cases, presence of cough ?14 days (20.5%) and paroxysmal coughing spells (33.3%) at diagnosis were uncommon. Only 16 (38%) B. pertussis-associated hospitalizations fulfilled the Centers for Diseases Control and Prevention case definition of definite pertussis. Conclusions. Bordetella pertussis contributed to a modest proportion of all-cause respiratory illness hospitalization among black-African children, with a trend for higher incidence among HIV-exposed than HIV-unexposed infants. Maternal vaccination of pregnant women should be considered to reduce the burden of pertussis hospitalization in this population. © The Author 2016.	English	Scopus
2022	Oskarsson, Y.; Haraldsson, A.; Oddsdottir, B.H.I.; Asgeirsdottir, T.L.; Thors, V.	Clinical and Socioeconomic Burden of Respiratory Syncytial Virus in Iceland	Pediatric Infectious Disease Journal	10.1097/INF.00000000000003640	Introduction: Respiratory syncytial virus (RSV), a very common pathogen, causes variable disease severity. In addition to considerable clinical burden on children, their families and healthcare facilities, RSV infections in children also carry significant direct and indirect socioeconomic burden. Methods: We analyzed data from 5 consecutive RSV seasons (2015-2020) and used virologically confirmed RSV infections and age <5 years as case definition. Clinical information was retrieved from electronic patient records. Costs were estimated by assuming an annual 30% attack rate and a combination of direct medical costs and calculations of societal costs of lost productivity. Results: A total of 716 children younger than 5 years of age had confirmed RSV infection of which 254 needed hospitalizations, most of whom were previously healthy. The median length of admission was 3.6 days and 13 patients needed intensive care. The hospital admission incidence rate was 2.5/1000 children/year, but 9.1 for children younger than 1 years of age. The total annual cost of RSV was estimated at €4.3 million, of which 10% was direct healthcare costs. Discussion: The clinical and socioeconomic disease burden of RSV in Iceland is substantial despite slightly lower hospital admission rates than other high-income countries. The prevention of RSV in young children, either through maternal or infant vaccination, has the potential to decrease both clinical and financial impact of the annual epidemics. © 2022 Lippincott Williams and Wilkins. All rights reserved.	English	Scopus
2018	Chittaganpitch, M.; Waicharoen, S.; Yingyong, T.; Praphasiri, P.; Sangkitporn, S.; Olsen, S.J.; Lindblade, K.A.	Viral etiologies of influenza-like illness and severe acute respiratory infections in Thailand	Influenza and other Respiratory Viruses	10.1111/irv.12554	Background: Information on the burden, characteristics and seasonality of non-influenza respiratory viruses is limited in tropical countries. Objectives: Describe the epidemiology of selected non-influenza respiratory viruses in Thailand between June 2010 and May 2014 using a sentinel surveillance platform established for influenza. Methods: Patients with influenza-like illness (ILI; history of fever or documented temperature ≥38°C, cough, not requiring hospitalization) or severe acute respiratory infection (SARI; history of fever or documented temperature ≥38°C, cough, onset <10 days, requiring hospitalization) were enrolled from 10 sites. Throat swabs were tested for influenza viruses, respiratory syncytial virus (RSV), metapneumovirus (MPV), parainfluenza viruses (PIV) 1-3, and adenoviruses by polymerase chain reaction (PCR) or real-time reverse transcriptase-PCR. Results: We screened 15 369 persons with acute respiratory infections and enrolled 8106 cases of ILI (5069 cases <15 years old) and 1754 cases of SARI (1404 cases <15 years old). Among ILI cases <15 years old, influenza viruses (1173, 23%), RSV (447, 9%), and adenoviruses (430, 8%) were the most frequently identified respiratory viruses tested, while for SARI cases <15 years old, RSV (196, 14%) influenza (157, 11%) and adenoviruses (90, 6%) were the most common. The RSV season significantly overlapped the larger influenza season from July to November in Thailand. Conclusions: The global expansion of influenza sentinel surveillance provides an opportunity to gather information on the characteristics of cases positive for non-influenza respiratory viruses, particularly seasonality, although adjustments to case definitions may be required. © 2018 The Authors. Influenza and Other Respiratory Viruses Published by John Wiley & Sons Ltd.	English	Scopus

2019	Rha, B.; Dahl, R.M.; Moyes, J.; Binder, A.M.; Tempia, S.; Walaza, S.; Bi, D.; Groome, M.J.; Variava, E.; Naby, F.; Kahn, K.; Treurnicht, F.; Cohen, A.L.; Gerber, S.I.; Madhi, S.A.; Cohen, C.	Performance of Surveillance Case Definitions in Detecting Respiratory Syncytial Virus Infection among Young Children Hospitalized with Severe Respiratory Illness - South Africa, 2009-2014	Journal of the Pediatric Infectious Diseases Society	10.1093/jpids/piy055	Background: Respiratory syncytial virus (RSV) is a leading cause of acute lower respiratory tract infection (ALRTI) in young children, but data on surveillance case definition performance in estimating burdens have been limited. Methods: We enrolled children aged <5 years hospitalized for ALRTI (or neonatal sepsis in young infants) through active prospective surveillance at 5 sentinel hospitals in South Africa and collected nasopharyngeal aspirates from them for RSV molecular diagnostic testing between 2009 and 2014. Clinical data were used to characterize RSV disease and retrospectively evaluate the performance of respiratory illness case definitions (including the World Health Organization definition for severe acute respiratory infection [SARI]) in identifying hospitalized children with laboratory-confirmed RSV according to age group (<3, 3-5, 6-11, 12-23, and 24-59 months). Results: Of 9969 hospitalized children, 2723 (27%) tested positive for RSV. Signs and symptoms in RSV-positive children varied according to age; fever was less likely to occur in children aged <3 months (57%; odds ratio [OR], 0.8 [95% CI, 0.7-0.9]) but more likely in those aged ≥12 months (82%; OR, 1.7-1.9) than RSV-negative children. The sensitivity (range, 55%-81%) and specificity (range, 27%-54%) of the SARI case definition to identify hospitalized RSV-positive children varied according to age; the lowest sensitivity was for infants aged <6 months. Using SARI as the case definition would have missed 36% of RSV-positive children aged <5 years and 49% of those aged <3 months; removing the fever requirement from the definition recovered most missed cases. Conclusion: Including fever in the SARI case definition lowers the sensitivity for RSV case detection among young children hospitalized with an ALRTI and likely underestimates its burden. © 2018 Published by Oxford University Press on behalf of The Journal of the Pediatric Infectious Diseases Society 2018.	English	Scopus
2011	Velasco, J.M.S.; Yoon, I.-K.; Mason, C.J.; Jarman, R.G.; Bodhidatta, L.; Klungthong, C.; Silapong, S.; Valderama, M.T.G.; Wongstitwilairoong, T.; Torres, A.G.; de Cecchis, D.P.; Pavlin, J.A.	Applications of PCR (real-time and masstag) and enzyme-linked immunosorbent assay in diagnosis of respiratory infections and diarrheal illness among deployed U.S. military personnel during exercise Balikatan 2009, Philippines	Military Medicine	10.7205/MILMED-D-11-00027	Laboratory-based surveillance for diarrheal and respiratory illness was conducted at the 2009 Republic of the Philippines-United States Balkatan exercise to determine the presence of specificpathogens endemic in the locations where the military exerci es were conducted. Ten stool and 6 respiratory specimens were obtained from individuals meeting case definitions for diarrhea or respiratory illness. Stool specimens were frozen in dry ice and remotely tested using enzyme-linked immunosorbent assay for Rotavirus, Astrovirus, Adenovirus, Entamoeba histolytica, Giardia, and Cryptosporidium and polymerase chain reaction for enterotoxigenic Escherichia coli, Campylobacter, Shigella, Vibrio, Salmonella, and Norovirus. Eight (4 for Campylobacter jejuni, 2 for Campylobacter coli, 1 for Norovirus genogroup II, and 1 for both Campylobacter coli and enterotoxigenic Escherichia coli) of 10 samples were positive for at least 1 enteric pathogen. MassTag polymerase chain reaction for influenza A and B, respiratory syncytial virus groups A and B, human coronavirus-229E and human coronavirus-OC43, human metapneumovirus, enterovirus, human parainfluenza viruses 2,3, and 4a, human adenovirus, Haemophilus influenzae, Neisseria meningitidis, Streptococcus pneumoniae, Legionella pneumonia, and Mycoplasma pneumonia was done on respiratory specimens. Out of 6 samples, 3 tested positive for H. influenzae; 1 tested positive for both H. influenzae and human parainfluenza virus 3; and 2 tested negative. Laboratory-based surveillance can be useful in determining etiologies of diarrheal and respiratory illness of deployed military personnel. © Association of Military Surgeons of the U.S. All rights reserved.	English	Scopus
2022	Rafferty, E.; Paulden, M.; Buchan, S.A.; Robinson, J.L.; Bettinger, J.A.; Kumar, M.; Svenson, L.W.; MacDonald, S.E.	Evaluating the Individual Healthcare Costs and Burden of Disease Associated with RSV Across Age Groups	PharmacoEconomics	10.1007/s40273-022-01142-w	Background: Respiratory syncytial virus (RSV) is a major cause of acute respiratory infection (ARI), with high morbidity and mortality worldwide. RSV costing and burden estimates can highlight the potential benefits of future vaccination programs and are essential for economic evaluations. Objective: We aimed to determine RSV healthcare costs across age groups and the overall disease burden of medically attended RSV in Canada. Methods: We conducted a retrospective case–control study to estimate the attributable healthcare costs per RSV case in Alberta. We used two case definitions to capture diversity in case severity: laboratory-confirmed RSV and ARI attributable to RSV. Matching occurred on five criteria: (1) age, (2) urban/rural status, (3) sex, (4) prematurity and (5) Charlson Comorbidity Index score. We calculated the age-specific burden of medically attended RSV in Canada from 2010 to 2019 by multiplying the weekly age-specific incidence of medically attended ARI with the RSV positivity rate. Results: Costs per laboratory-confirmed RSV case were (in Canadian dollars [CAD], year 2020 values) $CAD12,713 and 40,028 in the first 30 and 365 days following diagnosis, respectively, whereas a case of ARI potentially attributable to RSV cost $CAD316 and 915, in 30 and 365 days, respectively. Older (aged ≥ 65 years) and younger (aged < 90 days) age groups had the highest case costs. The average medically attended RSV incidence rate across nine seasons was 1743 cases per 100,000 people per year. Conclusions: RSV is a common and expensive infection at the extremes of life, and the development of immunization programs targeting older and younger ages may be important for the reduction of RSV burden and cost. © 2022, The Author(s).	English	Scopus
2021	Yamasaki, L.; Moi, M.L.	Complexities in case definition of sars-cov-2 reinfection: Clinical evidence and implications in covid-19 surveillance and diagnosis	Pathogens	10.3390/pathogens10101262	Reinfection cases have been reported in some countries with clinical symptoms ranging from mild to severe. In addition to clinical diagnosis, virus genome sequence from the first and second infection has to be confirmed to either belong to separate clades or had significant mutations for the confirmation of SARS-CoV-2 reinfection. While phylogenetic analysis with paired specimens offers the strongest evidence for reinfection, there remains concerns on the definition of SARS-CoV-2 reinfection, for reasons including accessibility to paired-samples and technical challenges in phylogenetic analysis. In light of the emergence of new SARS-CoV-2 variants that are associated with increased transmissibility and immune-escape further understanding of COVID-19 protective im-munity, real-time surveillance directed at identifying COVID-19 transmission patterns, transmissi-bility of emerging variants and clinical implications of reinfection would be important in addressing the challenges in definition of COVID-19 reinfection and understanding the true disease burden. © 2021 by the authors. Licensee MDPI, Basel, Switzerland.	English	Scopus
2020	Vanderburg, S.; Wijayaratne, G.; Danthanarayana, N.; Jayamaha, J.; Piyasiri, B.; Halloluwa, C.; Sheng, T.; Amarasena, S.; Kurukulasooriya, R.; Nicholson, B.P.; Peiris, J.S.M.; Gray, G.C.; Gunasena, S.; Nagahawatte, A.; Bodinayake, C.K.; Woods, C.W.; Devasiri, V.; Tillekeratne, L.G.	Outbreak of severe acute respiratory infection in Southern Province, Sri Lanka in 2018: A cross-sectional study	BMJ Open	10.1136/bmjopen-2020-040612	Objectives To determine aetiology of illness among children and adults presenting during outbreak of severe respiratory illness in Southern Province, Sri Lanka, in 2018. Design Prospective, cross-sectional study. Setting 1600-bed, public, tertiary care hospital in Southern Province, Sri Lanka. Participants 410 consecutive patients, including 371 children and 39 adults, who were admitted with suspected viral pneumonia (passive surveillance) or who met case definition for acute respiratory illness (active surveillance) in May to June 2018. Results We found that cocirculation of influenza A (22.6% of cases), respiratory syncytial virus (27.8%) and adenovirus (AdV) (30.7%; type B3) was responsible for the outbreak. Mortality was noted in 4.5% of paediatric cases identified during active surveillance. Virus type and viral coinfection were not significantly associated with mortality. Conclusions This is the first report of intense cocirculation of multiple respiratory viruses as a cause of an outbreak of severe acute respiratory illness in Sri Lanka, and the first time that AdV has been documented as a cause of a respiratory outbreak in the country. Our results emphasise the need for continued vigilance in surveying for known and emerging respiratory viruses in the tropics. ©	English	Scopus
2017	Chughtai, A.A.; Wang, Q.; Dung, T.C.; Macintyre, C.R.	The presence of fever in adults with influenza and other viral respiratory infections	Epidemiology and Infection	10.1017/S0950268816002181	SUMMARY We compared the rates of fever in adult subjects with laboratory-confirmed influenza and other respiratory viruses and examined the factors that predict fever in adults. Symptom data on 158 healthcare workers (HCWs) with a laboratory-confirmed respiratory virus infection were collected using standardized data collection forms from three separate studies. Overall, the rate of fever in confirmed viral respiratory infections in adult HCWs was 23·4% (37/158). Rates varied by virus: human rhinovirus (25·3%, 19/75), influenza A virus (30%, 3/10), coronavirus (28·6%, 2/7), human metapneumovirus (28·6%, 2/7), respiratory syncytial virus (14·3%, 4/28) and parainfluenza virus (8·3%, 1/12). Smoking [relative risk (RR) 4·65, 95% confidence interval (CI) 1·33-16·25] and co-infection with two or more viruses (RR 4·19, 95% CI 1·21-14·52) were significant predictors of fever. Fever is less common in adults with confirmed viral respiratory infections, including influenza, than described in children. More than 75% of adults with a viral respiratory infection do not have fever, which is an important finding for clinical triage of adult patients with respiratory infections. The accepted definition of 'influenza-like illness' includes fever and may be insensitive for surveillance when high case-finding is required. A more sensitive case definition could be used to identify adult cases, particularly in event of an emerging viral infection. Copyright © Cambridge University Press 2016Â This is an Open Access article, distributed under the terms of the Creative Commons Attribution licence (http://creativecommons.org/licenses/by/4.0/), which permits unrestricted re-use, distribution, and reproduction in any medium, provided the original work is properly cited..	English	Scopus

2012	Kammerer, P.E.; Montiel, S.; Kriner, P.; Bojorquez, I.; Bejarano Ramirez, V.; Vazquez-Erlbeck, M.; Azziz-Baumgartner, E.; Blair, P.J.	Influenza-like illness surveillance on the California-Mexico border, 2004-2009	Influenza and other Respiratory Viruses	10.1111/j.1750-2659.2011.00316.x	Background Since 2004, the Naval Health Research Center, with San Diego and Imperial counties, has collaborated with the US Centers for Disease Control and Prevention to conduct respiratory disease surveillance in the US-Mexico border region. In 2007, the Secretariat of Health, Mexico and the Institute of Public Health of Baja California joined the collaboration. Objectives The identification of circulating respiratory pathogens in respiratory specimens from patients with influenza-like illness (ILI). Methods Demographic, symptom information and respiratory swabs were collected from enrollees who met the case definition for ILI. Specimens underwent PCR testing and culture in virology and bacteriology. Results From 2004 through 2009, 1855 persons were sampled. Overall, 36% of the participants had a pathogen identified. The most frequent pathogen was influenza (25%), with those aged 6-15years the most frequently affected. In April 2009, a young female participant from Imperial County, California, was among the first documented cases of 2009 H1N1. Additional pathogens included influenza B, adenovirus, parainfluenza virus, respiratory syncytial virus, enterovirus, herpes simplex virus, Streptococcus pneumoniae, and Streptococcus pyogenes. Conclusions The US-Mexico border is one of the busiest in the world, with a large number of daily crossings. Due to its traffic, this area is an ideal location for surveillance sites. We identified a pathogen in 36% of the specimens tested, with influenza A the most common pathogen. A number of other viral and bacterial respiratory pathogens were identified. An understanding of the incidence of respiratory pathogens in border populations is useful for development of regional vaccination and disease prevention responses. © 2011 Blackwell Publishing Ltd.	English	Scopus
2012	Neu, N.; Plaskett, T.; Hutcheon, G.; Murray, M.; Southwick, K.L.; Saiman, L.	Epidemiology of human metapneumovirus in a pediatric long-term care facility	Infection Control and Hospital Epidemiology	10.1086/665727	Background. Viral respiratory pathogens cause outbreaks in pediatric long-term care facilities (LTCFs), but few studies have used viral diagnostic testing to identify the causative pathogens. We describe the use of such testing during a prolonged period of respiratory illness and elucidate the epidemiology of human metapneumovirus (hMPV) at our LTCF. design. Retrospective study of influenza-like illness (ILI). setting. A 136-bed pediatric LTCF from January 1 through April 30, 2010. methods. The ILI case definition included fever, cough, change in oropharyngeal secretions, increase in oxygen requirement, and/or wheezing. results. During the study period, 69 episodes of ILI occurred in 61 (41%) of 150 residents. A viral pathogen was detected in 27 (39%) of the episodes, including respiratory syncytial virus (RSV) (n = 3 n = 2), influenza A virus (not typed;), parainfluenza virus (n = 2), adenovirus (n = 1), and hMPV (n = 19). Twenty-seven of the residents with ILI (44%) required transfer to acute care hospitals (mean length of hospitalization, 12 days; range, 3-47 days). Residents with tracheostomies were more likely to have ILI (adjusted odds ratio [OR], 3.99 [95% confidence interval {CI}, 1.87-8.53]; P=.0004). The mortality rate for residents with ILI was 1.6%. Residents with hMPV were younger (P=.03), more likely to be transferred to an acute care facility (OR, 3.73 [95% CI, 1.17-11.95]; P=.02), and less likely to have a tracheostomy (adjusted OR, 0.19 [95% CI, 0.047-0.757]; P=.02). discussion. Diverse pathogens, most notably hMPV, caused ILI in our pediatric LTCF during a prolonged period of time. Viral testing was helpful in characterizing the epidemiology of ILI in this population. © 2012 by The Society for Healthcare Epidemiology of America. All rights reserved.	English	Scopus
2021	Koskela, U.; Helve, O.; Sarvikivi, E.; Helminen, M.; Nieminen, T.; Peltola, V.; Renko, M.; Saxén, H.; Pasma, H.; Pokka, T.; Honkila, M.; Tapiainen, T.	Multi-inflammatory syndrome and Kawasaki disease in children during the COVID-19 pandemic: A nationwide register-based study and time series analysis	Acta Paediatrica, International Journal of Paediatrics	10.1111/apa.16051	Aim: We investigated whether the ongoing COVID-19 pandemic was associated with the occurrence of Kawasaki disease or with multi-inflammatory syndrome in children (MIS-C). Methods: This national Finnish register-based study was based on laboratory-confirmed severe acute respiratory syndrome coronavirus 2 (SARS-CoV-2) infections, MIS-C and Kawasaki disease cases. We performed a time series analysis on the occurrence of Kawasaki disease in 2016–2020. Results: In 2020, there were 5170 laboratory-confirmed COVID-19 cases in children under 18 years of age and five fulfilled the MIS-C case definition. The occurrence of MIS-C was 0.97 per 1000 (95% confidence interval: 0.31-2.26) laboratory-confirmed SARS-CoV-2 infections in children. Our time series analysis showed that Kawasaki disease cases decreased during the COVID-19 pandemic. The seasonally adjusted incidence rate ratio was 0.49 (95% confidence interval: 0.32-0.74) when it was compared to pre-pandemic levels. This coincided with a reduced occurrence of respiratory infections, due to social distancing in the population. Conclusion: This nationwide register-based study found that MIS-C was a rare complication of the SARS-CoV-2 infection. The occurrence of Kawasaki disease and respiratory infections decreased during the pandemic. This suggests that transmissible microbes may play an important role in Kawasaki disease and social distancing may have a protective effect. © 2021 The Authors. Acta Paediatrica published by John Wiley & Sons Ltd on behalf of Foundation Acta Paediatrica	English	Scopus
2021	Vasconcelos, M.K.; Loens, K.; Sigfrid, L.; Iosifidis, E.; Epalza, C.; Donà, D.; Matheeussen, V.; Papachristou, S.; Roilides, E.; Gijon, M.; Rojo, P.; Minotti, C.; Da Dalt, L.; Islam, S.; Jarvis, J.; Syggelou, A.; Tsolia, M.; Nyang'wa, M.N.; Keers, S.; Renk, H.; Gemmel, A.-L.; D'Amore, C.; Atti, M.C.D.; Sánchez, C.R.-T.; Martinón-Torres, F.; Burokiene, S.; Goetghebuer, T.; Spoulou, V.; Riordan, A.; Calvo, C.; Gkentzi, D.; Hufnagel, M.; Openshaw, P.J.; De Jong, M.D.; Koopmans, M.; Goossens, H.; Ieven, M.; Fraaij, P.L.A.; Giaquinto, C.; Bielicki, J.A.; Horby, P.; Sharland, M.	Aetiology of acute respiratory infection in preschool children requiring hospitalisation in Europe-results from the PED-MERMAIDS multicentre case-control study	BMJ Open Respiratory Research	10.1136/bmjresp-2021-000887	Background Both pathogenic bacteria and viruses are frequently detected in the nasopharynx (NP) of children in the absence of acute respiratory infection (ARI) symptoms. The aim of this study was to estimate the aetiological fractions for ARI hospitalisation in children for respiratory syncytial virus (RSV) and influenza virus and to determine whether detection of specific respiratory pathogens on NP samples was associated with ARI hospitalisation. Methods 349 children up to 5 years of age hospitalised for ARI (following a symptom-based case definition) and 306 hospital controls were prospectively enrolled in 16 centres across seven European Union countries between 2016 and 2019. Admission day NP swabs were analysed by multiplex PCR for 25 targets. Results RSV was the leading single cause of ARI hospitalisations, with an overall population attributable fraction (PAF) of 33.4% and high seasonality as well as preponderance in younger children. Detection of RSV on NP swabs was strongly associated with ARI hospitalisation (OR adjusted for age and season: 20.6, 95% CI: 9.4 to 45.3). Detection of three other viral pathogens showed strong associations with ARI hospitalisation: influenza viruses had an adjusted OR of 6.1 (95% CI: 2.5 to 14.9), parainfluenza viruses (PIVs) an adjusted OR of 4.6 (95% CI: 1.8 to 11.3) and metapneumoviruses an adjusted OR of 4.5 (95% CI: 1.3 to 16.1). Influenza viruses had a PAF of 7.9%, PIVs of 6.5% and metapneumoviruses of 3.0%. In contrast, most other pathogens were found in similar proportions in cases and controls, including Streptococcus pneumoniae, which was weakly associated with case status, and endemic coronaviruses. Conclusion RSV is the predominant cause of ARI hospitalisations in young children in Europe and its detection, as well as detection of influenza virus, PIV or metapneumovirus, on NP swabs can establish aetiology with high probability. PAFs for RSV and influenza virus are highly seasonal and age dependent. © 2016 Georg Thieme Verlag. All rights reserved.	English	Scopus
2019	Omer, S.B.; Bednarczyk, R.; Kazi, M.; Beryl Guterman, L.; Aziz, F.; Allen, K.E.; Yildirim, I.; Asad Ali, S.	Assessment and Validation of Syndromic Case Definitions for Respiratory Syncytial Virus Testing in a Low Resource Population	Pediatric Infectious Disease Journal	10.1097/INF.0000000000002159	Standardized case definitions are needed in decision-making regarding respiratory syncytial virus control strategies, including vaccine evaluation. A syndromic case definition comprising of "wheeze or apnea or cyanosis" could be useful for community-based surveillance of moderate respiratory syncytial virus infection among young infants particularly in resource-limited settings. However, this definition showed modest specificity (29.2%-49.6%), indicating that community-based surveillance may need augmentation with other data. © 2018 Wolters Kluwer Health, Inc. All rights reserved.	English	Scopus
2007	De Souza Luna, L.K.; Panning, M.; Grywna, K.; Pfefferle, S.; Drosten, C.	Spectrum of viruses and atypical bacteria in intercontinental air travelers with symptoms of acute respiratory infection	Journal of Infectious Diseases	10.1086/511432	Respiratory infections after air travel are frequent, but epidemiological data are incomplete. Using sensitive polymerase chain reactions, we studied the spectrum of atypical bacteria and respiratory viruses in travelers fulfilling the case definition of severe acute respiratory syndrome. A pathogen was identified in 67 travelers (43.2%). Influenza and parainfluenza viruses were most prevalent, at 14.2% and 15.5%, respectively. Prevalences of adenoviruses, human metapneumovirus, coronaviruses, and rhinoviruses ranged between 2.6% and 4.8%. Human bocavirus, respiratory syncytial virus, and Legionella, Mycoplasma, and Chlamydophila species were absent or appeared at frequencies of <1%. To our knowledge, these are the first specific baseline data for the mentioned agents in the context of air travel. © 2007 by the Infectious Diseases Society of America. All rights reserved.	English	Scopus

2011	Smit, P.M.; Limper, M.; van Gorp, E.C.M.; Smits, P.H.M.; Beijnen, J.H.; Brandjes, D.P.M.; Mulder, J.W.	Adult outpatient experience of the 2009 H1N1 pandemic: Clinical course, pathogens, and evaluation of case definitions	Journal of Infection	10.1016/j.jinf.2011.03.005	Objectives: The aim was to describe causative agents and clinical characteristics in adult outpatients with upper airway symptoms during the 2009 H1N1 pandemic and to evaluate case definitions that are used in clinical practice. Methods: From August through December 2009, 964 symptomatic adult outpatients were included. RT-PCR was used to detect the following pathogens: influenza A (H1N1) and B, parainfluenza 1-4, adenovirus, respiratory syncytial virus, human rhinovirus, human metapneumovirus, human coronavirus (OC43, 229E, NL63), Chlamydia pneumoniae, Mycoplasma pneumoniae and Legionella species. The Dutch GHOR, American CDC and WHO, and British HPA case definitions were evaluated. Results: A respiratory pathogen was detected in 41% of tested patient samples; influenza A (H1N1) and human rhinovirus were both detected in 16%. Clinical presentation of influenza cases was significantly more serious when compared to rhinovirus or negative-tested cases. Test characteristics were almost similar for all 4 case definitions, with an average sensitivity of 66%, specificity of 70%, positive predictive value of 34% and negative predictive value of 90%. Conclusions: Influenza A (H1N1) and human rhinovirus were the major pathogens responsible for respiratory disease. The 2009 H1N1 pandemic in Amsterdam followed a mild course. Test characteristics of 4 different clinical case definitions seemed comparable but rather useless. © 2011 The British Infection Association.	English	Scopus
2022	Ramay, B.M.; Jara, J.; Moreno, M.P.; Lupo, P.; Serrano, C.; Alvis, J.P.; Arriola, C.S.; Veguilla, V.; Kaydos-Daniels, S.C.	Self-medication and ILI etiologies among individuals presenting at pharmacies with influenza-like illness: Guatemala City, 2018 influenza season	BMC Public Health	10.1186/s12889-022-13962-8	Objectives: We aimed to characterize the proportion of clients presenting to community pharmacies with influenza-like illness (ILI) and the severity of their illness; the proportion with detectable influenza A, influenza B, and other pathogens (i.e., parainfluenza I, II, and III, adenovirus, respiratory syncytial virus, human metapneumovirus); and to describe their self-medication practices. Methods: A cross-sectional study was conducted in six pharmacies in Guatemala City. Study personnel collected nasopharyngeal and oropharyngeal swabs from participants who met the ILI case definition and who were self-medicating for the current episode. Participants were tested for influenza A and B and other pathogens using real-time RT-PCR. Participants' ILI-associated self-medication practices were documented using a questionnaire. Results: Of all patients entering the pharmacy during peak hours who responded to a screening survey (n = 18,016) 6% (n = 1029) self-reported ILI symptoms, of which 45% (n = 470/1029) met the study case definition of ILI. Thirty-one percent (148/470) met inclusion criteria, of which 87% (130/148) accepted participation and were enrolled in the study. Among 130 participants, nearly half tested positive for viral infection (n = 55, 42.3%) and belonged to groups at low risk for complications from influenza. The prevalence of influenza A was 29% (n = 35). Thirteen percent of the study population (n = 17) tested positive for a respiratory virus other than influenza. Sixty-four percent of participants (n = 83) reported interest in receiving influenza vaccination if it were to become available in the pharmacy. Medications purchased included symptom-relieving multi-ingredient cold medications (n = 43/100, 43%), nonsteroidal anti-inflammatory drugs (n = 23, 23%), and antibiotics (n = 16, 16%). Antibiotic use was essentially equal among antibiotic users regardless of viral status. The broad-spectrum antibiotics ceftriaxone and azithromycin were the most common antibiotics purchased. Conclusions: During a typical influenza season, a relatively low proportion of all pharmacy visitors were experiencing influenza symptoms. A high proportion of clients presenting to pharmacies with ILI tested positive for a respiratory virus. Programs that guide appropriate use of antibiotics in this population are needed and become increasingly important during pandemics caused by respiratory viral pathogens. © 2022, The Author(s).	English	Scopus
2015	Saha, S.; Pandey, B.G.; Choudekar, A.; Krishnan, A.; Gerber, S.I.; Rai, S.K.; Singh, P.; Chadha, M.; Lal, R.B.; Broor, S.	Evaluation of case definitions for estimation of respiratory syncytial virus associated hospitalizations among children in a rural community of northern India	Journal of Global Health	10.7189/jogh.05.020419	Background The burden estimation studies for respiratory syncytial virus (RSV) have been based on varied case definitions, including case-definitions designed for influenza surveillance systems. We used all medical admissions among children aged 0-59 months to study the effect of case definitions on estimation of RSV-associated hospitalizations rates. Methods The hospital-based daily surveillance enrolled children aged 0-59 months admitted with acute medical conditions from July 2009-December 2012, from a well-defined rural population in Ballabgarh in northern India. All study participants were examined and nasal and throat swabs taken for testing by real-time polymerase chain reaction (RT-PCR) for RSV and influenza virus. Clinical data were used to retrospectively evaluate World Health Organization (WHO) case definitions (2011) commonly used for surveillance of respiratory pathogens, ie, acute respiratory illness (WHO-ARI), severe ARI (SARI) and influenza-like illness (ILI), for determination of RSV-associated hospitalization. RSV-associated hospitalization rates adjusted for admissions at non-study hospitals were calculated. Findings Out of 505 children enrolled, 82 (16.2%) tested positive for RSV. Annual incidence rates of RSV-associated hospitalization per 1000 children were highest among infants aged 0-5 months (15.2; 95% confidence interval (CI) 8.3-26.8), followed by ages 6-23 months (5.3, 95% CI 3.2-8.7) and lowest among children 24-59 months (0.5, 95% CI 0.1-1.5). The RSV positive children were more likely to have signs of respiratory distress like wheeze, chest in-drawing, tachypnea, and crepitation compared to RSV-negative based on bivariate comparisons. Other less commonly seen signs of respiratory distress, ie, nasal flaring, grunting, accessory muscle usage were also significantly associated with being RSV positive. Compared to the estimated RSV hospitalization rate based on all medical hospitalizations, the WHO-ARI case definition captured 86% of the total incidence, while case definitions requiring fever like ILI and SARI underestimated the incidence by 50-80%. Conclusions: Our study suggests that RSV is a substantial cause of hospitalization among children aged lt;24months especially those aged lt;6 months. The WHO-ARI case definition appeared to be the most suitable screening definition for RSV surveillance because of its high sensitivity.	English	Scopus
2015	Penttinen, P.; Pebody, R.	Influenza case definitions – optimising sensitivity and specificity	Eurosurveillance	10.2807/1560-7917.ES2015.20.22.21148		English	Scopus
2020	Subissi, L.; Bossuyt, N.; Reynders, M.; Gerard, M.; Dauby, N.; Bourgeois, M.; Delaere, B.; Quoilin, S.; Van Gucht, S.; Thomas, I.; Barbezange, C.	Capturing respiratory syncytial virus season in Belgium using the influenza severe acute respiratory infection surveillance network, season 2018/19	Eurosurveillance	10.2807/1560-7917.ES.2020.25.39.1900627	Background: Respiratory syncytial virus (RSV) is a common cause of severe respiratory illness in young children (< 5 years old) and older adults (≥ 65 years old) leading the World Health Organization (WHO) to recommend the implementation of a dedicated surveillance in countries. Aim: We tested the capacity of the severe acute respiratory infection (SARI) hospital network to contribute to RSV surveillance in Belgium. Methods: During the 2018/19 influenza season, we started the SARI surveillance for influenza in Belgium in week 40, earlier than in the past, to follow RSV activity, which usually precedes influenza virus circulation. While the WHO SARI case definition for influenza normally used by the SARI hospital network was employed, flexibility over the fever criterion was allowed, so patients without fever but meeting the other case definition criteria could be included in the surveillance. Results: Between weeks 40 2018 and 2 2019, we received 508 samples from SARI patients. We found an overall RSV detection rate of 62.4% (317/508), with rates varying depending on the age group: 77.6% in children aged < 5 years (253/326) and 34.4% in adults aged ≥ 65 years (44/128). Over 90% of the RSV-positive samples also positive for another tested respiratory virus (80/85) were from children aged < 5 years. Differences were also noted between age groups for symptoms, comorbidities and complications. Conclusion: With only marginal modifications in the case definition and the period of surveillance, the Belgian SARI network would be able to substantially contribute to RSV surveillance and burden evaluation in children and older adults, the two groups of particular interest for WHO. © 2020 European Centre for Disease Prevention and Control (ECDC). All rights reserved.	English	Scopus
2012	Feikin, D.R.; Njenga, M.K.; Bigogo, G.; Aura, B.; Aol, G.; Audi, A.; Jagero, G.; Muluare, P.O.; Gikunju, S.; Nderitu, L.; Balish, A.; Winchell, J.; Schneider, E.; Erdman, D.; Oberste, M.S.; Katz, M.A.; Breiman, R.F.	Etiology and incidence of viral and bacterial acute respiratory illness among older children and adults in rural western kenya, 2007-2010	PLoS ONE	10.1371/journal.pone.0043656	Background: Few comprehensive data exist on disease incidence for specific etiologies of acute respiratory illness (ARI) in older children and adults in Africa. Methodology/Principal Findings: From March 1, 2007, to February 28, 2010, among a surveillance population of 21,420 persons >5 years old in rural western Kenya, we collected blood for culture and malaria smears, nasopharyngeal and oropharyngeal swabs for quantitative real-time PCR for ten viruses and three atypical bacteria, and urine for pneumococcal antigen testing on outpatients and inpatients meeting a ARI case definition (cough or difficulty breathing or chest pain and temperature >38.0°C or oxygen saturation <90% or hospitalization). We also collected swabs from asymptomatic controls, from which we calculated pathogen-attributable fractions, adjusting for age, season, and HIV-status, in logistic regression. We calculated incidence by pathogen, adjusting for health-seeking for ARI and pathogen-attributable fractions. Among 3,406 ARI patients >5 years old (adjusted annual incidence 12.0 per 100 person-years), influenza A virus was the most common virus (22% overall; 11% inpatients, 27% outpatients) and Streptococcus pneumoniae was the most common bacteria (16% overall; 23% inpatients, 14% outpatients), yielding annual incidences of 2.6 and 1.7 episodes per 100 person-years, respectively. Influenza A virus, influenza B virus, respiratory syncytial virus (RSV) and human metapneumovirus were more prevalent in swabs among cases (22%, 6%, 8% and 5%, respectively) than controls. Adenovirus, parainfluenza viruses, rhinovirus/enterovirus, parechovirus, and Mycoplasma pneumoniae were not more prevalent among cases than controls. Pneumococcus and non-typhi Salmonella were more prevalent among HIV-infected adults, but prevalence of viruses was similar among HIV-infected and HIV-negative individuals. ARI incidence was highest during peak malaria season. Conclusions/Signficance: Vaccination against influenza and pneumococcus (by potential herd immunity from childhood vaccination or of HIV-infected adults) might prevent much of the substantial ARI incidence among persons <5 years old in similar rural African settings.	English	Scopus

2020	Wilmont, S.; Neu, N.; Hill-Ricciuti, A.; Alba, L.; Prill, M.M.; Whitaker, B.; Garg, S.; Stone, N.D.; Lu, X.; Kim, L.; Gerber, S.I.; Larson, E.; Saiman, L.	Active surveillance for acute respiratory infections among pediatric long-term care facility staff	American Journal of Infection Control	10.1016/j.ajic.2020.06.190	Background: Transmission of respiratory viruses between staff and residents of pediatric long-term care facilities (pLTCFs) can occur. We assessed the feasibility of using text or email messages to perform surveillance for acute respiratory infections (ARIs) among staff. Methods: From December 7, 2016 to May 7, 2017, 50 staff participants from 2 pLTCFs received weekly text or email requests to report the presence or absence of ARI symptoms. Those who fulfilled the ARI case definition (≥2 symptoms) had respiratory specimens collected to detect viruses by reverse transcriptase polymerase chain reaction assays. Pre- and postsurveillance respiratory specimens were collected to assess subclinical viral shedding. Results: The response rate to weekly electronic messages was 93%. Twenty-one ARIs reported from 20 (40%) participants fulfilled the case definition. Respiratory viruses were detected in 29% (5/17) of specimens collected at symptom onset (influenza B, respiratory syncytial virus, coronavirus [CoV] 229E, rhinovirus [RV], and dual detection of CoV OC43 and bocavirus). Four participants had positive presurveillance (4 RV), and 6 had positive postsurveillance specimens (3 RV, 2 CoV NL63, and 1 adenovirus). Conclusions: Electronic messaging to conduct ARI surveillance among pLTCF staff was feasible. © 2020	English	Scopus
2019	Akimkin, V.G.; Zakharova, Yu.A.; Igonina, E.P.; Bolgarova, E.V.	NOSOCOMIAL RESPIRATORY VIRAL INFECTIONS: STATE OF THE PROBLEMS	Zhurnal Mikrobiologii Epidemiologii i Immunobiologii	10.36233/0372-9311-2019-5-50-61	We scanned the PubMed search database for literature on the incidence of nosocomial respiratory viral infections (NRVI) published over a ten-year period. Necessity to apply the standard case definition and the laboratory panel based on the multiplex polymerase chain reaction in frequency assessment was established. In general, predominance of rhinoviruses in the etiological structure was detected. Rationale was given for introduction of nonspecific epidemic prevention activities against a broad spectrum of other respiratory pathogens including high-priority respiratory syncytial viruses, metapneumoviruses, adenoviruses, influenza and parainfluenza viruses and coronaviruses. Bocaviruses and mimiviruses were designated as rare species. The biological diversity of the pathogens causing NRVI calls for active promotion of molecular genetics techniques in the work of the laboratory services of health facilities to perform quality etiological diagnosis, design relevant antiviral therapy regimens and effective prevention programs whose implementation will lead to significant reduction in the spread risk of these infections and the treatment costs. © 2019, Central Research Institute for Epidemiology. All rights reserved.	Russian	Scopus
2019	Chavez, D.; Gonzales-Armayo, V.; Mendoza, E.; Palekar, R.; Rivera, R.; Rodriguez, A.; Salazar, C.; Veizaga, A.; Añez, A.	Estimation of influenza and respiratory syncytial virus hospitalizations using sentinel surveillance data—La Paz, Bolivia. 2012–2017	Influenza and other Respiratory Viruses	10.1111/irv.12663	Objective: The objective was to estimate the number of hospitalizations associated with influenza and RSV using data from severe acute respiratory infection (SARI) sentinel surveillance from El Alto-La Paz. Bolivia. Methods: All persons who met the case definition for SARI at one sentinel hospital had a clinical sample collected and analyzed by rRT-PCR for influenza and by indirect immunofluorescence for RSV. The SARI-influenza and SARI-RSV case counts were stratified by six age groups. The proportion of cases captured in the sentinel hospital in relation to the non-sentinel hospitals of area was multiplied by the age-specific census population, to build the denominators. The annual incidence and a 95% confidence interval (CI) were estimated. Results: During 2012-2017, n = 2606 SARI cases were reported (average incidence 120/100 000 inhabitants [95% CI: 116-124]); the average incidence of influenza-associated SARI hospitalization was 15.3/100 000 (95% CI: 14.1-16.7), and the average incidence of RSV-associated SARI hospitalization was 9/100 000 inhabitants (95% CI: 8.1-10.1). The highest incidence of influenza was among those less than one year of age (average 174.7/100 000 [range: 89.1-299.5]), followed by those one to four years of age (average 51.8/100 000 [range: 19.8-115.4]) and then those 65 years of age and older (average 47.7/100 000 [range: 18.8-117]). For RSV, the highest incidence was highest among those less than one year of age (231/100 000 [range: 119.9-322.9]). Conclusion: Influenza and RSV represent major causes of hospitalization in La Paz, Bolivia—with the highest burden among children under one year of age. Our estimates support current prevention strategies in this age group. © 2019 The Authors. Influenza and Other Respiratory Viruses Published by John Wiley & Sons Ltd.	English	Scopus
2020	Klink, T.; Rankin, D.A.; Piya, B.; Spieker, A.J.; Faouri, S.; Shehabi, A.; Williams, J.V.; Khuri-Bulos, N.; Halasa, N.B.	Evaluating the diagnostic accuracy of the WHO severe acute respiratory infection (SARI) criteria in Middle Eastern children under two years over three respiratory seasons	PLoS ONE	10.1371/journal.pone.0232188	Objective The World Health Organization created the Severe Acute Respiratory Infection (SARI) criteria in 2011 to monitor influenza (flu)-related hospitalization. Many studies have since used the SARI case definition as inclusion criteria for surveillance studies. We sought to determine the sensitivity, specificity, positive predictive value, and negative predictive value of the SARI criteria for detecting ten different respiratory viruses in a Middle Eastern pediatric cohort. Materials and methods The data for this study comes from a prospective acute respiratory surveillance study of hospitalized children <2 years in Amman, Jordan from March 16, 2010 to March 31, 2013. Participants were recruited if they had a fever and/or respiratory symptoms. Nasal and throat swabs were obtained and tested by real-time RT-PCR for eleven viruses. Subjects meeting SARI criteria were determined post-hoc. Sensitivity, specificity, positive predictive value, and negative predictive value of the SARI case definition for detecting ten different viruses were calculated and results were stratified by age. Results Of the 3,175 patients enrolled, 3,164 were eligible for this study, with a median age of 3.5 months, 60.4% male, and 82% virus-positive (44% RSV and 3.8% flu). The sensitivity and specificity of the SARI criteria for detecting virus-positive patients were 44% and 77.9%, respectively. Sensitivity of SARI criteria for any virus was lowest in children <3 months at 22.4%. Removing fever as a criterion improved the sensitivity by 65.3% for detecting RSV in children <3 months; whereas when cough was removed, the sensitivity improved by 45.5% for detecting flu in same age group. Conclusions The SARI criteria have poor sensitivity for detecting RSV, flu, and other respiratory viruses —particularly in children <3 months. Researchers and policy makers should use caution if using the criteria to estimate burden of disease in children. © 2020 Klink et al. This is an open access article distributed under the terms of the Creative Commons Attribution License, which permits unrestricted use, distribution, and reproduction in any medium, provided the original author and source are credited.	English	Scopus
2016	Chen, R.T.; Moro, P.L.; Bauwens, J.; Bonhoeffer, J.	Obstetrical and neonatal case definitions for immunization safety data	Vaccine	10.1016/j.vaccine.2016.08.026		English	Scopus
2020	Resch, B.; Puchas, C.; Resch, E.; Urlesberger, B.	Epidemiology of Respiratory Syncytial Virus-related Hospitalizations and the Influence of Viral Coinfections in Southern Austria in a 7-year Period	Pediatric Infectious Disease Journal	10.1097/INF.0000000000002494	Objective: The aim of this study was to determine the respiratory syncytial virus (RSV) epidemiology and to analyze the influence of risk factors and coinfections over the last years. Methods: Retrospectively all infants, children and adolescents hospitalized due to respiratory disease with positive RSV test [hospitalized for RSV infection (RSV-H)] between January 1, 2009, and December 31, 2015, at a tertiary care center in the southern part of Austria were included for analysis. Patients were all identified by a search via International Classification of Diseases and Related Health Problems, 10th Edition codes, and all medical data were collected from the local electronic databases called openMedocs. RSV tests had to prove true infection case definition. Results: During a 7-year study period, 745 infants, children and adolescents exhibited RSV-H. Main diagnosis was bronchiolitis (70%). Nearly half of all cases (44%) were born during the first half of the RSV season (November-January), and seasonal peak of RSV-H was in January. Predominant underlying condition was history of prematurity in 15% followed by neurologic impairment (3.5%) and hemodynamically significant congenital heart disease (2.95%). Age ≤2 months and underlying conditions/morbidities were associated with more severe disease. The majority of cases (96%) had an age below 24 months, and 91% below 12 months. Viral coinfection (most common influenza virus, adenovirus and rhinovirus) was diagnosed in 37 cases (5%) resulting in a more severe course of disease. Main risk factors of coinfection were siblings and crowding. Mortality was 0.27% (2/745). Both children had coinfection with influenza A virus and were multihandicapped (15 and 20 years of age, respectively). Conclusions: Prematurity and underlying morbidities play a marked role in RSV-H. Viral coinfections aggravated disease with death in 2 multihandicapped adolescents. © 2020 Lippincott Williams and Wilkins. All rights reserved.	English	Scopus
2016	Wansaula, Z.; Olsen, S.J.; Casal, M.G.; Golenko, C.; Erhart, L.M.; Kammerer, P.; Whitfield, N.; Mccotter, O.Z.	Surveillance for severe acute respiratory infections in Southern Arizona, 2010-2014	Influenza and other Respiratory Viruses	10.1111/irv.12360	Background: The Binational Border Infectious Disease Surveillance program began surveillance for severe acute respiratory infections (SARI) on the US-Mexico border in 2009. Here, we describe patients in Southern Arizona. Methods: Patients admitted to five acute care hospitals that met the SARI case definition (temperature ≥37·8°C or reported fever or chills with history of cough, sore throat, or shortness of breath in a hospitalized person) were enrolled. Staff completed a standard form and collected a nasopharyngeal swab which was tested for selected respiratory viruses by reverse transcription polymerase chain reaction. Results: From October 2010-September 2014, we enrolled 332 SARI patients. Fifty-two percent were male and 48% were white non-Hispanic. The median age was 63 years (47% ≥65 years and 5·2% <5 years). During hospitalization, 51 of 230 (22%) patients required intubation, 120 of 297 (40%) were admitted to intensive care unit, and 28 of 278 (10%) died. Influenza vaccination was 56%. Of 309 cases tested, 49 (16%) were positive for influenza viruses, 25 (8·1%) for human metapneumovirus, 20 (6·5%) for parainfluenza viruses, 16 (5·2%) for coronavirus, 11 (3·6%) for respiratory syncytial virus, 10 (3·2%) for rhinovirus, 4 (1·3%) for rhinovirus/enterovirus, 3 (1·0%) for enteroviruses, and 3 (1·0%) for adenovirus. Among the 49 influenza-positive specimens, 76% were influenza A (19 H3N2, 17 H1N1pdm09, and 1 not subtyped), and 24% were influenza B. Conclusion: Influenza viruses were a frequent cause of SARI in hospitalized patients in Southern Arizona. Monitoring respiratory illness in border populations will help better understand the etiologies. Improving influenza vaccination coverage may help prevent some SARI cases. © 2016 John Wiley & Sons Ltd..	English	Scopus

2019	Sáez-López, E.; Pechirra, P.; Costa, I.; Cristóvão, P.; Conde, P.; Machado, A.; Rodrigues, A.P.; Guiomar, R.	Performance of surveillance case definitions for respiratory syncytial virus infections through the sentinel influenza surveillance system, Portugal, 2010 to 2018	Eurosurveillance	10.2807/1560-7917.ES.2019.24.45.1900140	Background: Well-established influenza surveillance systems (ISS) can be used for respiratory syncytial virus (RSV) surveillance. In Portugal, RSV cases are detected through the ISS using the European Union (EU) influenza-like illness (ILI) case definition. Aim: To investigate clinical predictors for RSV infection and how three case definitions (EU ILI, a modified EU acute respiratory infection, and one respiratory symptom) performed in detecting RSV infections in Portugal. Methods: This observational retrospective study used epidemiological and laboratory surveillance data (October 2010–May 2018). Associations between clinical characteristics and RSV detection were analysed using logistic regression. Accuracy of case definitions was assessed through sensitivity, specificity, and area under the receiver operating characteristic curve (AUC). A 0.05 significance level was accepted. Results: The study involved 6,523 persons, including 190 (2.9%) RSV cases. Among 183 cases with age information, RSV infection was significantly more frequent among individuals < 5 years (n = 23; 12.6%) and ≥ 65 years (n = 45; 24.6%) compared with other age groups (p < 0.0001). Cough (odds ratio (OR): 2.4; 95% confidence interval (CI): 1.2–6.5) was the best RSV-infection predictor considering all age groups, while shortness of breath was particularly associated with RSV-positivity among ≤ 14 year olds (OR: 6.7; 95% CI: 2.6–17.4 for 0–4 year olds and OR: 6.7; 95% CI: 1.5–28.8 for 5–14 year olds). Systemic symptoms were significantly associated with RSV-negative and influenza-positive cases. None of the case definitions were suitable to detect RSV infections (AUC = 0.51). Conclusion: To avoid underestimating the RSV disease burden, RSV surveillance within the Portuguese sentinel ISS would require a more sensitive case definition than ILI and, even a different case definition according to age. © 2019 European Centre for Disease Prevention and Control (ECDC). All rights reserved.	English	Scopus
2008	Beilei, N.; Carraro, E.; Perosa, A.; Watanabe, A.; Arruda, E.; Granato, C.	Acute respiratory infection and influenza-like illness viral etiologies in Brazilian adults	Journal of Medical Virology	10.1002/jmv.21295	Influenza-like illness (ILI) definitions have been used worldwide for influenza surveillance. These different case definitions can vary with regard to sensitivity and predictive values for laboratory confirmed influenza. The literature has indicated the inclusion of other viruses may be the cause of these variable results. The objective of the study was to evaluate ILI national sentinel criteria and viral etiologies in adults diagnosed with acute respiratory infection (ARI) and/or ILI from 2001 to 2003 in Sao Paulo, Brazil. Clinical and laboratory evaluations were observed from 420 adults and collected on a daily basis from outpatient care units at University Hospital. The ILI definition included: fever plus at least one respiratory symptom (cough and/or sore throat) and one constitutional symptom (headache, malaise, myalgia, sweat or chills, or fatigue). DFA and RT-PCR for influenza, parainfluenza, respiratory syncytial virus, adenovirus, enterovirus, coronavirus, rhinovirus, and metapneumovirus were performed on nasal washes and 61.8% resulted positive. The respiratory viruses detected most often were influenza and rhinovirus. ILI was reported for 240/420 patients (57.1%), with influenza and rhinovirus etiologies accounting for 30.9% and 19.6%, respectively. Rhinovirus peak activity was concurrent with the influenza season. These findings highlight the implications of other viruses in ILI etiology and suggest that during the influenza season, this clinical overlap must be considered in the diagnosis and clinical management of patients. © 2008 Wiley-Liss, Inc.	English	Scopus
2016	Atwell, J.E.; Geoghegan, S.; Karron, R.A.; Polack, F.P.	Clinical predictors of critical lower respiratory tract illness due to respiratory syncytial virus in infants and children: Data to inform case definitions for efficacy trials	Journal of Infectious Diseases	10.1093/infdis/jiw447	We analyzed data from 524 Argentinean infants hospitalized with lower respiratory tract illness (LRTI) due to respiratory syncytial virus (RSV) to inform selection of clinical end points for RSV vaccine efficacy trials. Cases of LRTI due to RSV that required a mask, continuous or bilevel positive airway pressure, or mechanical ventilation were classified as critical. Oxygen saturation of =90%, tachypnea, and tachycardia were each associated with an increased odds of critical LRTI due to RSV (adjusted odds ratios [ORs], 2.30 [95% confidence interval {CI}, 1.26-4.24; P =.007], 2.22 [95% CI, 1.19-4.16; P =.012], and 2.35 [95% CI, 1.22-4.50; P =.010], respectively). The odds of critical LRTI due to RSV increased substantially (OR, 8.57; 95% CI, 2.19-73.5; P =.001) among individuals with ≥2 indicators. Lower chest wall indrawing was not associated with critical disease. © 2016 The Author.	English	Scopus
2016	Nyawanda, B.O.; Mott, J.A.; Njuguna, H.N.; Mayieka, L.; Khagayi, S.; Onkoba, R.; Makokha, C.; Otieno, N.A.; Bigogo, G.M.; Katz, M.A.; Feikin, D.R.; Verani, J.R.	Evaluation of case definitions to detect respiratory syncytial virus infection in hospitalized children below 5 years in Rural Western Kenya, 2009-2013	BMC Infectious Diseases	10.1186/s12879-016-1532-0	Background: In order to better understand respiratory syncytial virus (RSV) epidemiology and burden in tropical Africa, optimal case definitions for detection of RSV cases need to be identified. Methods: We used data collected between September 2009 - August 2013 from children aged <5years hospitalized with acute respiratory Illness at Siaya County Referral Hospital. We evaluated the sensitivity, specificity, positive predictive value (PPV) and negative predictive value (NPV) of individual signs, symptoms and standard respiratory disease case definitions (severe acute respiratory illness [SARI]; hospitalized influenza-like illness [hILI]; integrated management of childhood illness [IMCI] pneumonia) to detect laboratory-confirmed RSV infection. We also evaluated an alternative case definition of cough or difficulty breathing plus hypoxia, in-drawing, or wheeze. Results: Among 4714 children hospitalized with ARI, 3810 (81%) were tested for RSV; and 470 (12%) were positive. Among individual signs and symptoms, cough alone had the highest sensitivity to detect laboratory-confirmed RSV [96%, 95% CI (95-98)]. Hypoxia, wheezing, stridor, nasal flaring and chest wall in-drawing had sensitivities ranging from 8 to 31%, but had specificities >75%. Of the standard respiratory case definitions, SARI had the highest sensitivity [83%, 95% CI (79-86)] whereas IMCI severe pneumonia had the highest specificity [91%, 95% CI (90-92)]. The alternative case definition (cough or difficulty breathing plus hypoxia, in-drawing, or wheeze) had a sensitivity of [55%, 95% CI (50-59)] and a specificity of [60%, 95% CI (59-62)]. The PPV for all case definitions and individual signs/symptoms ranged from 11 to 20% while the negative predictive values were >87%. When we stratified by age < 1 year and 1- < 5years, difficulty breathing, severe pneumonia and the alternative case definition were more sensitive in children aged <1year [70% vs. 54%, p < 0.01], [19% vs. 11%, p = 0.01] and [66% vs. 43%, p < 0.01] respectively, while non-severe pneumonia was more sensitive [14% vs. 26%, p < 0.01] among children aged 1- < 5years. Conclusion: The sensitivity and specificity of different commonly used case definitions for detecting laboratory-confirmed RSV cases varied widely, while the positive predictive value was consistently low. Optimal choice of case definition will depend upon study context and research objectives. © 2016 Nyawanda et al.	English	Scopus
2019	La Vincente, S.F.; Von Mollendorf, C.; Ulziibayar, M.; Satzke, C.; Dashtseren, L.; Fox, K.K.; Dunne, E.M.; Nguyen, C.D.; De Campo, J.; De Campo, M.; Thomson, H.; Surenkhand, G.; Demberelsuren, S.; Bujinlkham, S.; Do, L.A.H.; Narangerel, D.; Cherian, T.; Mungun, T.; Mulholland, E.K.	Evaluation of a phased pneumococcal conjugate vaccine introduction in Mongolia using enhanced pneumonia surveillance and community carriage surveys: A study protocol for a prospective observational study and lessons learned	BMC Public Health	10.1186/s12889-019-6639-y	Background: Streptococcus pneumoniae causes substantial morbidity and mortality among children. The introduction of pneumococcal conjugate vaccines (PCV) has the potential to dramatically reduce disease burden. As with any vaccine, it is important to evaluate PCV impact, to help guide decision-making and resource-allocation. Measuring PCV impact can be complex, particularly to measure impact on one of the most common and significant diseases caused by the pneumococcus, namely pneumonia. Here we outline the protocol developed to evaluate the impact of 13-valent PCV (PCV13) on childhood pneumonia in Mongolia, and a number of lessons learned in implementing the evaluation that may be helpful to other countries seeking to undertake pneumonia surveillance. Methods: From 2016 PCV13 was introduced in a phased manner into the routine immunisation programme with some catch-up by the Government of Mongolia. We designed an evaluation to measure vaccine impact in children aged 2-59 months with hospitalised radiological pneumonia as a primary outcome, with secondary objectives to measure impact on clinically-defined pneumonia, nasopharyngeal carriage of S. pneumoniae among pneumonia patients and in the community, and severe respiratory infection associated with RSV and/or influenza. We enhanced an existing hospital-based pneumonia surveillance system by incorporating additional study components (nasopharyngeal swabbing using standard methods, C-reactive protein, risk factor assessment) and strengthening clinical practices, such as radiology as well as monitoring and training. We conducted cross-sectional community carriage surveys to provide data on impact on carriage among healthy children. Discussion: Establishing a robust surveillance system is an important component of monitoring the impact of PCV within a country. The enhanced surveillance system in Mongolia will facilitate assessment of PCV13 impact on pneumonia, with radiological confirmed disease as the primary outcome. Key lessons arising from this evaluation have included the importance of establishing a core group of in-country staff to be responsible for surveillance activities and to work closely with this team; to be aware of external factors that could potentially influence disease burden estimates; to be flexible in data collection processes to respond to changing circumstances and lastly to ensure a consistent application of the pneumonia surveillance case definition throughout the study period. © 2019 The Author(s).	English	Scopus

2022	Davis, W.; Duque, J.; Huang, Q.S.; Olson, N.; Grant, C.C.; Newbern, E.C.; Thompson, M.; Waite, B.; Prasad, N.; Trenholme, A.; Azziz-Baumgartner, E.	Sensitivity and specificity of surveillance case definitions in detection of influenza and respiratory syncytial virus among hospitalized patients, New Zealand, 2012–2016	Journal of Infection	10.1016/j.jinf.2021.12.012	Background: The WHO is exploring the value of adding RSV testing to existing influenza surveillance systems to inform RSV control programs. We evaluate the usefulness of four commonly used influenza surveillance case-definitions for influenza and RSV surveillance. Methods: SHIVERS, a multi-institutional collaboration, conducted surveillance for influenza and RSV in four New Zealand hospitals. Nurses reviewed admission logs, enrolled patients with suspected acute respiratory infections (ARI), and obtained nasopharyngeal swabs for RT-PCR. We compared the performance characteristics for identifying laboratory-confirmed influenza and RSV severe acute respiratory infection (SARI), defined as persons admitted with measured or reported fever and cough within 10 days of illness, to three other case definitions: 1. reported fever and cough or shortness of breath, 2. cough and shortness of breath, or 3. cough. Results: During April-September 2012–2016, SHIVERS identified 16,055 admissions with ARI; of 6374 cases consented and tested for influenza or RSV, 5437 (85%) had SARI and 937 (15%) did not. SARI had the highest specificity in detecting influenza (40.6%) and RSV (40.8%) but the lowest sensitivity (influenza 78.8%, RSV 60.3%) among patients of all ages. Cough or shortness of breath had the highest sensitivity (influenza 99.3%, RSV 99.9%) but the lowest specificity (influenza 1.6%, RSV 1.9%). SARI sensitivity among children aged <3 months was 60.8% for influenza and 43.6% for RSV–both lower than in other age groups. Conclusions: While SARI had the highest specificity, its sensitivity was limited, especially among children aged <3 months. Cough or shortness of breath was the most sensitive. © 2021	English	Scopus
2014	Emukule, G.O.; Khagayi, S.; McMorrow, M.L.; Ochola, R.; Otieno, N.; Widdowson, M.-A.; Ochieng, M.; Feikin, D.R.; Katz, M.A.; Mott, J.A.	The burden of influenza and rsv among inpatients and outpatients in rural western Kenya, 2009-2012	PLoS ONE	10.1371/journal.pone.0105543	Background: In Kenya, detailed data on the age-specific burden of influenza and RSV are essential to inform use of limited vaccination and treatment resources. Methods: We analyzed surveillance data from August 2009 to July 2012 for hospitalized severe acute respiratory illness (SARI) and outpatient influenza-like illness (ILI) at two health facilities in western Kenya to estimate the burden of influenza and respiratory syncytial virus (RSV). Incidence rates were estimated by dividing the number of cases with laboratory-confirmed virus infections by the mid-year population. Rates were adjusted for healthcare-seeking behavior, and to account for patients who met the SARI/ILI case definitions but were not tested. Results: The average annual incidence of influenza-associated SARI hospitalization per 1,000 persons was 2.7 (95% CI 1.8-3.9) among children <5 years and 0.3 (95% CI 0.2-0.4) among persons ≥5 years; for RSV-associated SARI hospitalization, it was 5.2 (95% CI 4.0-6.8) among children ,5 years and 0.1 (95% CI 0.0-0.2) among persons ≥5 years. The incidence of influenza-associated medically-attended ILI per 1,000 was 24.0 (95% CI 16.6-34.7) among children <5 years and 3.8 (95% CI 2.6-5.7) among persons ≥5 years. The incidence of RSV-associated medically-attended ILI was 24.6 (95% CI 17.0-35.4) among children <5 years and 0.8 (95% CI 0.3-1.9) among persons ≥5 years. Conclusions: Influenza and RSV both exact an important burden in children. This highlights the possible value of influenza vaccines, and future RSV vaccines, for Kenyan children. © 2014 Emukule et al.	English	Scopus
2022	von Mollendorf, C.; Berger, D.; Gwee, A.; Duke, T.; Graham, S.M.; Russell, F.M.; Mulholland, E.K.	Aetiology of childhood pneumonia in lowand middle-income countries in the era of vaccination: a systematic review	Journal of Global Health	10.7189/JOGH.12.10009	Background This systematic review aimed to describe common aetiologies of severe and non-severe community acquired pneumonia among children aged 1 month to 9 years in low and middle-income countries. Methods We searched the MEDLINE, EMBASE, and PubMed online databases for studies published from January 2010 to August 30, 2020. We included studies on acute community-acquired pneumonia or acute lower respiratory tract infection with ≥1 year of continuous data collection; clear consistent case definition for pneumonia; >1 specimen type (except empyema studies where only pleural fluid was required); testing for >1 pathogen including both viruses and bacteria. Two researchers reviewed the studies independently. Results were presented as a narrative summary. Quality of evidence was assessed with the Quality Assessment Tool for Quantitative Studies. The study was registered on PROSPERO [CRD42020206830]. Results We screened 5184 records; 1305 duplicates were removed. The remaining 3879 titles and abstracts were screened. Of these, 557 articles were identified for full-text review, and 55 met the inclusion criteria – 10 case-control studies, three post-mortem studies, 11 surveillance studies, eight cohort studies, five cross-sectional studies, 12 studies with another design and six studies that included patients with pleural effusions or empyema. Studies which described disease by severity showed higher bacterial detection (Streptococcus pneumoniae, Staphylococcus aureus) in severe vs non-severe cases. The most common virus causing severe disease was respiratory syncytial virus (RSV). Pathogens varied by age, with RSV and adenovirus more common in younger children. Influenza and atypical bacteria were more common in children 5-14 years than younger children. Malnourished and HIV-infected children had higher rates of pneumonia due to bacteria or tuberculosis. Conclusions Several viral and bacterial pathogens were identified as important targets for prevention and treatment. Bacterial pathogens remain an important cause of moderate to severe disease, particularly in children with comorbidities despite widespread PCV and Hib vaccination. © World Health Organization [2021]. Licensee (International Society of Global Health) This is an open access article distributed under the terms of the Creative Commons Attribution IGO License (http://creativecommons.org/licenses/by/3.0/igo/legalcode), which permits unrestricted use, distribution, and reproduction in any medium, provided the original work is properly cited. In any reproduction of this article there should not be any suggestion that WHO or this article endorse any specific organisation or products. The use of the WHO logo is not permitted.	English	Scopus
2020	Hirve, S.; Crawford, N.; Palekar, R.; Zhang, W.; Bancej, C.; Barr, I.; Baumeister, E.; Broor, S.; Burmaa, A.; Campbell, H.; Caetano, B.; Chadha, M.; Chittaganpitch, M.; Coulibaly, D.; Darmaa, B.; Ellis, J.; Fahim, M.; Fasce, R.; Herve, K.; Jackson, S.; Pisareva, M.; Moyes, J.; Naguib, A.; Nair, H.; Pebody, R.; Potdar, V.; Rajatonirina, S.; Siqueira, M.; Smith, P.G.; Smorodintseva, E.; Sotomayor, V.; Treurnicht, F.; Tivane, A.; Venter, M.; Wairagkar, N.; Zambon, M.	Clinical characteristics, predictors, and performance of case definition—Interim results from the WHO global respiratory syncytial virus surveillance pilot	Influenza and other Respiratory Viruses	10.1111/irv.12688	Background: The lack of a uniform surveillance case definition poses a challenge to characterize the epidemiology, clinical features, and disease burden of the respiratory syncytial virus (RSV). Global standards for RSV surveillance will inform immunization policy when RSV vaccines become available. Methods: The WHO RSV surveillance pilot leverages the capacities of the Global Influenza Surveillance and Response System (GISRS). Hospitalized and non-hospitalized medically attended patients of any age were tested for RSV using standardized molecular diagnostics throughout the year in fourteen countries. An extended severe acute respiratory infection (extended SARI) or an acute respiratory infection (ARI) case definition was used that did not require fever as a criterion. Results: Amongst 21 221 patients tested for RSV between January 2017 and September 2018, 15 428 (73%) were hospital admissions. Amongst hospitalized RSV-positive patients, 50% were aged <6 months and 88% <2 years. The percentage of patients testing positive for RSV was 37% in children <6 months and 25% in those aged 6 months to 2 years. Patients with fever were less likely to be RSV positive compared to those without fever (OR 0.74; 95% CI: 0.63-0.86). For infants <6 months, 29% of RSV ARI cases did not have fever. Conclusion: Requiring fever in a case definition for RSV lowers the sensitivity to detect cases in young children. Countries should consider ways to leverage the GISRS platform to implement RSV surveillance with an augmented case definition amongst the young pediatric population. © 2020 The Authors. Influenza and Other Respiratory Viruses Published by John Wiley & Sons Ltd.	English	Scopus
2022	Hoang, U.; Button, E.; Armstrong, M.; Okusi, C.; Ellis, J.; Zambon, M.; Anand, S.; Delanerolle, G.; Hobbs, F.D.R.; van Summeren, J.; Paget, J.; de Lusignan, S.	Assessing the Clinical and Socioeconomic Burden of Respiratory Syncytial Virus in Children Aged Under 5 Years in Primary Care: Protocol for a Prospective Cohort Study in England and Report on the Adaptations of the Study to the COVID-19 Pandemic	JMIR Research Protocols	10.2196/38026	Background: Respiratory syncytial virus (RSV) commonly causes lower respiratory tract infections and hospitalization in children. In 2019-2020, the Europe-wide RSV ComNet standardized study protocol was developed to measure the clinical and socioeconomic disease burden of RSV infections among children aged <5 years in primary care. RSV has a recognized seasonality in England. Objective: We aimed to describe (1) the adaptations of the RSV ComNet standardized study protocol for England and (2) the challenges of conducting the study during the COVID-19 pandemic. Methods: This study was conducted by the Oxford-Royal College of General Practitioners Research and Surveillance Centre-the English national primary care sentinel network. We invited all (N=248) general practices within the network that undertook virology sampling to participate in the study by recruiting eligible patients (registered population: n=3,056,583). Children aged <5 years with the following case definition of RSV infection were included in the study: those consulting a health care practitioner in primary care with symptoms meeting the World Health Organization's definition of acute respiratory illness or influenza-like illness who have laboratory-confirmed RSV infection. The parents/guardians of these cases were asked to complete 2 previously validated questionnaires (14 and 30 days postsampling). A sample size of at least 100 RSV-positive cases is required to estimate the percentage of children that consult in primary care who need hospitalization. Assuming a swab positivity rate of 20% in children aged <5 years, we estimated that 500 swabs are required. We adapted our method for the pandemic by extending sampling planned for winter 2020-2021 to a rolling data collection, allowing verbal consent and introducing home swabbing because of increased web-based consultations during the COVID-19 pandemic. Results: The preliminary results of the data collection between International Organization for Standardization (ISO) weeks 1-41 in 2021 are described. There was no RSV detected in the winter of 2020-2021 through the study. The first positive RSV swab collected through the sentinel network in England was collected in ISO week 17 and then every week since ISO week 25. In total, 16 (N=248, 6.5%) of the virology-sampling practices volunteered to participate; these were high-sampling practices collecting the majority of eligible swabs across the sentinel network-200 (43.8%) out of 457 swabs, of which 54 (N=200, 27%) were positive for RSV. Conclusions: Measures to control the COVID-19 pandemic meant there was no circulating RSV last winter; however, RSV has circulated out of season, as detected by the sentinel network. The sentinel network practices have collected 40% (200/500) of the required samples, and 27% (54/200) were RSV positive. We have demonstrated the feasibility of implementing a European-standardized RSV disease burden study protocol in England during a pandemic, and we now need to recruit to this adapted protocol. © 2022 JMIR Publications. All rights reserved.	English	Scopus

2010	Pierangeli, A.; Scagnolari, C.; Gentile, M.; Spina, M.T.; Iudicello, A.; Bertazzoni, G.; Antonelli, G.	Virological diagnosis of respiratory virus infection in patients attending an emergency department during the influenza season	Clinical Microbiology and Infection	10.1111/j.1469-0691.2009.03119.x	To investigate the burden of influenza-like illness (ILI), patients attending an emergency department during the influenza season were tested for several common respiratory viruses, using PCR-based methods. Influenza A viruses were detected in 25 of 103 recruited patients (24%), rhinoviruses in 15%, and respiratory syncytial virus in only one. The data suggest that triage criteria based on ILI case definitions would not contain the spread of the influenza virus during pandemic alerts and could lead to unnecessary isolation of patients with other infections. Application of broader triage criteria followed by timely molecular diagnosis could be effective in preventing new respiratory agent transmission. © 2010 The Authors. Journal Compilation © 2010 European Society of Clinical Microbiology and Infectious Diseases.	English	Scopus
2017	Amini, R.; Gilca, R.; Douville-Fradet, M.; Boulianne, N.; De Serres, G.	Evaluation of the new world health organization case definition of severe acute respiratory infection for influenza surveillance during the peak weeks of two influenza seasons in Quebec, Canada	Journal of the Pediatric Infectious Diseases Society	10.1093/jpids/piw044	During the peak of the 2012-2013 and 2014-2015 influenza seasons in Quebec, Canada, the sensitivity of the new World Health Organization (WHO) case definition of severe acute respiratory infection (SARI) in < 5-year-old children was 65% for polymerase chain reaction-confirmed influenza and 79% for other respiratory viruses (ORVs), whereas its specificity and positive predictive value were approximately 2- and 4-fold lower for influenza than ORVs (25% vs 40% and 18% vs 76%, respectively). The use of the WHO SARI definition for influenza surveillance in children should be interpreted with caution according to the specific surveillance goals. © The Author 2017. Published by Oxford University Press on behalf of The Journal of the Pediatric Infectious Diseases Society. All rights reserved.	English	Scopus
2009	Gaspard, P.; Mosnier, A.; Cohen, J.-M.; Gunther, D.; Roth, C.; Eschbach, E.; Stoll-Keller, F.; Gayet, S.	Immunoassay rapid test for influenza diagnosis in institutions for elderly people: A four-year surveillance with the Grog Géronto-Alsace	Pathologie Biologie	10.1016/j.patbio.2008.08.007	Objective: Outbreaks of acute respiratory infections (ARI) are common in institutions for elderly people. The objective of our study was the assessment of immunoassay rapid test used for influenza diagnosis in institutions for elderly people. Methodology: Prospective surveillance for ARI was conducted in 11 institutions in Alsace over a four-year period. Clinical case definitions are used to identify the infected residents. For the identification of influenza virus, nasopharyngeal swabs are obtained and rapid tests (immunoassay) are performed. Results: Influenza virus was identified with immunoassay rapid test. Then, prophylaxis according to the Conseil supérieur d'hygiène publique de France guidelines was implemented. Nevertheless, the use of the rapid test was not frequent in the individual institution and the information recorded at the Grog Géronto-Alsace level could be use to inform the institutions when it is important to perform these rapid tests. Conclusion: Ours findings show the value of the rapid test used in the influenza surveillance and how the networks could help to improve their uses. © 2008 Elsevier Masson SAS. All rights reserved.	French	Scopus
2022	Boender, T.S.; Cai, W.; Schranz, M.; Kocher, T.; Wagner, B.; Ullrich, A.; Buda, S.; Zöllner, R.; Greiner, F.; Diercke, M.; Grabenhenrich, L.	Using routine emergency department data for syndromic surveillance of acute respiratory illness, Germany, week 10 2017 until week 10 2021	Eurosurveillance	10.2807/1560-7917.ES.2022.27.27.2100865	Background: The COVID-19 pandemic expanded the need for timely information on acute respiratory illness at population level. Aim: We explored the potential of routine emergency department data for syndromic surveillance of acute respiratory illness in Germany. Methods: We used routine attendance data from emergency departments, which continuously transferred data between week102017 and 102021, with ICD-10 codes available for>75% of attendances. Case definitions for acute respiratory infection (ARI), severe acute respiratory infection (SARI), influenza-like illness (ILI), respiratory syncytial virus infection (RSV) and COVID-19 were based on a combination of ICD-10 codes, and/or chief complaints, sometimes combined with information on hospitalisation and age. Results: We included 1,372,958 attendances from eight emergency departments. The number of attendances dropped in March 2020 during the first COVID-19 pandemic wave, increased during summer, and declined again during the resurge of COVID-19 cases in autumn and winter of 2020/21. A pattern of seasonality of respiratory infections could be observed. By using different case definitions (i.e. for ARI, SARI, ILI, RSV) both the annual influenza seasons in the years 2017–2020 and the dynamics of the COVID-19 pandemic in 2020/21 were apparent. The absence of the 2020/21 influenza season was visible, parallel to the resurge of COVID-19 cases. SARI among ARI cases peaked in April–May 2020 (17%) and November 2020–January 2021 (14%). Conclusion: Syndromic surveillance using routine emergency department data can potentially be used to monitor the trends, timing, duration, magnitude and severity of illness caused by respiratory viruses, including both influenza viruses and SARS-CoV-2. © 2022 European Centre for Disease Prevention and Control (ECDC). All rights reserved.	English	Scopus
2018	Schanzer, D.L.; Saboui, M.; Lee, L.; Nwosu, A.; Bancej, C.	Burden of influenza, respiratory syncytial virus, and other respiratory viruses and the completeness of respiratory viral identification among respiratory inpatients, Canada, 2003-2014	Influenza and other Respiratory Viruses	10.1111/irv.12497	Background: A regression-based study design has commonly been used to estimate the influenza burden; however, these estimates are not timely and many countries lack sufficient virological data. Alternative approaches that would permit a timelier assessment of the burden, including a sentinel surveillance approach recommended by the World Health Organization (WHO), have been proposed. We aimed to estimate the hospitalization burden attributable to influenza, respiratory syncytial virus (RSV), and other respiratory viruses (ORV) and to assess both the completeness of viral identification among respiratory inpatients in Canada and the implications of adopting other approaches. Methods: Respiratory inpatient records were extracted from the Canadian Discharge Abstract Database from 2003 to 2014. A regression model was used to estimate excess respiratory hospitalizations attributable to influenza, RSV, and ORV by age group and diagnostic category and compare these estimates with the number with a respiratory viral identification. Results: An estimated 33 (95% CI: 29, 38), 27 (95% CI: 22, 33), and 27 (95% CI: 18, 36) hospitalizations per 100 000 population per year were attributed to influenza, RSV, and ORV, respectively. An influenza virus was identified in an estimated 78% (95% CI: 75, 81) and 17% (95% CI: 15, 21) of respiratory hospitalizations attributed to influenza for children and adults, respectively, and 75% of influenza-attributed hospitalizations had an ARI diagnosis. Conclusions: Hospitalization rates with respiratory viral identification still underestimate the burden. Approaches based on acute respiratory case definitions will likely underestimate the burden as well, although each proposed method should be compared with regression-based estimates of influenza-attributed burden as a way of assessing their validity. © 2017 The Authors. Influenza and Other Respiratory Viruses. Published by John Wiley & Sons Ltd.	English	Scopus
2012	Ahmed, J.A.; Katz, M.A.; Auko, E.; Njenga, M.K.; Weinberg, M.; Kapella, B.K.; Burke, H.; Nyoka, R.; Gichangi, A.; Waiboci, L.W.; Mahamud, A.; Qassim, M.; Swai, B.; Wagacha, B.; Mutonga, D.; Nguhi, M.; Breiman, R.F.; Eidex, R.B.	Epidemiology of respiratory viral infections in two long-term refugee camps in Kenya, 2007-2010	BMC Infectious Diseases	10.1186/1471-2334-12-7	Background: Refugees are at risk for poor outcomes from acute respiratory infections (ARI) because of overcrowding, suboptimal living conditions, and malnutrition. We implemented surveillance for respiratory viruses in Dadaab and Kakuma refugee camps in Kenya to characterize their role in the epidemiology of ARI among refugees.Methods: From 1 September 2007 through 31 August 2010, we obtained nasopharyngeal (NP) and oropharyngeal (OP) specimens from patients with influenza-like illness (ILI) or severe acute respiratory infections (SARI) and tested them by RT-PCR for adenovirus (AdV), respiratory syncytial virus (RSV), human metapneumovirus (hMPV), parainfluenza viruses (PIV), and influenza A and B viruses. Definitions for ILI and SARI were adapted from those of the World Health Organization. Proportions of cases associated with viral aetiology were calculated by camp and by clinical case definition. In addition, for children < 5 years only, crude estimates of rates due to SARI per 1000 were obtained.Results: We tested specimens from 1815 ILI and 4449 SARI patients (median age = 1 year). Proportion positive for virus were AdV, 21.7%; RSV, 12.5%; hMPV, 5.7%; PIV, 9.4%; influenza A, 9.7%; and influenza B, 2.6%; 49.8% were positive for at least one virus. The annual rate of SARI hospitalisation for 2007-2010 was 57 per 1000 children per year. Virus-positive hospitalisation rates were 14 for AdV; 9 for RSV; 6 for PIV; 4 for hMPV; 5 for influenza A; and 1 for influenza B. The rate of SARI hospitalisation was highest in children < 1 year old (156 per 1000 child-years). The ratio of rates for children < 1 year and 1 to < 5 years old was 3.7:1 for AdV, 5.5:1 for RSV, 4.4:1 for PIV, 5.1:1 for hMPV, 3.2:1 for influenza A, and 2.2:1 for influenza B. While SARI hospitalisation rates peaked from November to February in Dadaab, no distinct seasonality was observed in Kakuma.Conclusions: Respiratory viral infections, particularly RSV and AdV, were associated with high rates of illness and make up a substantial portion of respiratory infection in these two refugee settings. © 2012 Ahmed et al; licensee BioMed Central Ltd.	English	Scopus

2017	Feikin, D.R.; Hammitt, L.L.; Murdoch, D.R.; O'Brien, K.L.; Scott, J.A.G.	The enduring challenge of determining pneumonia etiology in children: Considerations for future research priorities	Clinical Infectious Diseases	10.1093/cid/cix143	Pneumonia kills more children each year worldwide than any other disease. Nonetheless, accurately determining the causes of childhood pneumonia has remained elusive. Over the past century, the focus of pneumonia etiology research has shifted from studies of lung aspirates and postmortem specimens intent on identifying pneumococcal disease to studies of multiple specimen types distant from the lung that are tested for multiple pathogens. Some major challenges facing modern pneumonia etiology studies include the use of nonspecific and variable case definitions, poor access to pathologic lung tissue and to specimens from fatal cases, poor diagnostic accuracy of assays (especially when testing nonpulmonary specimens), and the interpretation of results when multiple pathogens are detected in a given individual. The future of childhood pneumonia etiology research will likely require integrating data from complementary approaches, including applications of advanced molecular diagnostics and vaccine probe studies, as well as a renewed emphasis on lung aspirates from radiologically confirmed pneumonia and postmortem examinations. © The Author 2017.	English	Scopus
2014	Radin, J.M.; Hawksworth, A.W.; Kammerer, P.E.; Balansay, M.; Raman, R.; Lindsay, S.P.; Brice, G.T.	Epidemiology of pathogen-specific respiratory infections among three US populations	PLoS ONE	10.1371/journal.pone.0114871	Background: Diagnostic tests for respiratory infections can be costly and time-consuming. Improved characterization of specific respiratory pathogens by identifying frequent signs, symptoms and demographic characteristics, along with improving our understanding of coinfection rates and seasonality, may improve treatment and prevention measures. Methods: Febrile respiratory illness (FRI) and severe acute respiratory infection (SARI) surveillance was conducted from October 2011 through March 2013 among three US populations: civilians near the US-Mexico border, Department of Defense (DoD) beneficiaries, and military recruits. Clinical and demographic questionnaire data and respiratory swabs were collected from participants, tested by PCR for nine different respiratory pathogens and summarized. Age stratified characteristics of civilians positive for influenza and recruits positive for rhinovirus were compared to other and no/unknown pathogen. Seasonality and coinfection rates were also described. Results: A total of 1444 patients met the FRI or SARI case definition and were enrolled in this study. Influenza signs and symptoms varied across age groups of civilians. Recruits with rhinovirus had higher percentages of pneumonia, cough, shortness of breath, congestion, cough, less fever and longer time to seeking care and were more likely to be male compared to those in the no/unknown pathogen group. Coinfections were found in 6% of all FRI/SARI cases tested and were most frequently seen among children and with rhinovirus infections. Clear seasonal trends were identified for influenza, rhinovirus, and respiratory syncytial virus. Conclusions: The age-stratified clinical characteristics associated with influenza suggest that age-specific case definitions may improve influenza surveillance and identification. Improving identification of rhinoviruses, the most frequent respiratory infection among recruits, may be useful for separating out contagious individuals, especially when larger outbreaks occur. Overall, describing the epidemiology of pathogen specific respiratory diseases can help improve clinical diagnoses, establish baselines of infection, identify outbreaks, and help prioritize the development of new vaccines and treatments. © 2014, Public Library of Science. All rights reserved.	English	Scopus
2022	Martinón-Torres, F.; Carmo, M.; Platero, L.; Drago, G.; López-Belmonte, J.L.; Bangert, M.; Díez-Domingo, J.; Garcés-Sánchez, M.	Clinical and economic burden of respiratory syncytial virus in Spanish children: the BARI study	BMC Infectious Diseases	10.1186/s12879-022-07745-0	Respiratory syncytial virus (RSV) infection is a major cause of morbidity in children. However, its disease burden remains poorly understood, particularly outside of the hospital setting. Our study aimed to estimate the burden of medically attended acute lower respiratory infection (ALRI) cases potentially related to RSV in Spanish children. Longitudinal data from September 2017 to June 2018 of 51,292 children aged < 5 years old from the National Healthcare System (NHS) of two Spanish regions were used. Three case definitions were considered: (a) RSV-specific; (b) RSV-specific and unspecified acute bronchiolitis (RSV-specific and Bronchiolitis), and; (c) RSV-specific and unspecified ALRI (RSV-specific and ALRI). A total of 3460 medically attended ALRI cases potentially due to RSV were identified, of which 257 (7.4%), 164 (4.7%), and 3039 (87.8%) coded with RSV-specific, unspecific bronchiolitis, and unspecific ALRI codes, respectively. Medically attended RSV-specific and ALRI cases per 1000 children was 134.4 in the first year of life, 119.4 in the second, and 35.3 between 2 and 5 years old. Most cases were observed in otherwise healthy children (93.1%). Mean direct healthcare cost per medically attended RSV-specific and ALRI case was €1753 in the first year of life, €896 in the second, and €683 between 2 and 5 years old. Hospitalization was the main driver of these costs, accounting for 55.6%, 38.0% and 33.4%, in each respective age group. In RSV-specific cases, mean direct healthcare cost per medically attended case was higher, mostly due to hospitalization: €3362 in the first year of life (72.9% from hospitalizations), €3252 in the second (72.1%), and €3514 between 2 and 5 years old (74.2%). These findings suggest that hospitalization data alone will underestimate the RSV infections requiring medical care, as will relying only on RSV-specific codes. RSV testing and codification must be improved and preventive solutions adopted, to protect all infants, particularly during the first year of life. © 2022, The Author(s).	English	Scopus
2021	Teirlinck, A.C.; Broberg, E.K.; Berg, A.S.; Campbell, H.; Reeves, R.M.; Carnahan, A.; Lina, B.; Pakarna, G.; Bøås, H.; Nohynek, H.; Emborg, H.-D.; Nair, H.; Reiche, J.; Oliva, J.A.; O'Gorman, J.; Paget, J.; Szymanski, K.; Danis, K.; Socan, M.; Gijon, M.; Rapp, M.; Havlíčková, M.; Trebbien, R.; Guiomar, R.; Hirve, S.S.; Buda, S.; van der Werf, S.; Meijer, A.; Fischer, T.K.	Recommendations for respiratory syncytial virus surveillance at the national level	European Respiratory Journal	10.1183/13993003.03766-2020	Respiratory syncytial virus (RSV) is a common cause of acute lower respiratory tract infections and hospitalisations among young children and is globally responsible for many deaths in young children, especially in infants aged <6 months. Furthermore, RSV is a common cause of severe respiratory disease and hospitalisation among older adults. The development of new candidate vaccines and monoclonal antibodies highlights the need for reliable surveillance of RSV. In the European Union (EU), no up-to-date general recommendations on RSV surveillance are currently available. Based on outcomes of a workshop with 29 European experts in the field of RSV virology, epidemiology and public health, we provide recommendations for developing a feasible and sustainable national surveillance strategy for RSV that will enable harmonisation and data comparison at the European level. We discuss three surveillance components: active sentinel community surveillance, active sentinel hospital surveillance and passive laboratory surveillance, using the EU acute respiratory infection and World Health Organization (WHO) extended severe acute respiratory infection case definitions. Furthermore, we recommend the use of quantitative reverse transcriptase PCR-based assays as the standard detection method for RSV and virus genetic characterisation, if possible, to monitor genetic evolution. These guidelines provide a basis for good quality, feasible and affordable surveillance of RSV. Harmonisation of surveillance standards at the European and global level will contribute to the wider availability of national level RSV surveillance data for regional and global analysis, and for estimation of RSV burden and the impact of future immunisation programmes. © The authors 2021.	English	Scopus
2007	Bellei, N.; Carraro, E.; Perosa, A.; Granato, C.	Patterns of influenza infections among different risk groups in Brazil	Brazilian Journal of Infectious Diseases	10.1590/S1413-86702007000400005	Influenza virus infections are associated with high morbidity and mortality. Influenza activity varies worldwide, and regional detection is influenced by geographic conditions, demograihic and patient-risk factors. We assessed influenza activity and patterns of seasonality during three consecutive years (2001-2003) in three risk groups in São Paulo city. Four-hundred-twelve outpatients with acute respiratory infection were subjected to epidemiological, clinical and laboratory investigations; these included community population (N=140), health-care workers (N=203), and renal-transplanted patients (N=69). Nasal wash samples were tested by direct fluorescent assay for influenza, parainfluenza, adenovirus, and respiratory syncytial virus. Overall Influenza positivity was 21%, and a progressive decline was observed in all groups over time. Influenza A and B co-circulated at the same time in 2001 and 2002, but not in 2003. Low influenza-vaccination rates (19%) were reported by health-care workers. Unexpected low levels of etiological agents were detected in renal-transplanted patients, and infected cases were less symptomatic than immunocompetent patients. Based on this study, we conclude that health-care worker-immunization programs should be implemented and the clinical patterns of infected influenza patients should be used as a guide for better case-definition criteria for adequate influenza surveillance, particularly for renal-transplant patients. © 2007 by The Brazilian Journal of Infectious Diseases and Contexto Publishing. All rights reserved.	English	Scopus
